# Three-dimensional protonic conductivity in porous organic cage solids

**DOI:** 10.1038/ncomms12750

**Published:** 2016-09-13

**Authors:** Ming Liu, Linjiang Chen, Scott Lewis, Samantha Y. Chong, Marc A. Little, Tom Hasell, Iain M. Aldous, Craig M. Brown, Martin W. Smith, Carole A. Morrison, Laurence J. Hardwick, Andrew I. Cooper

**Affiliations:** 1 Department of Chemistry and Centre for Materials Discovery, University of Liverpool, Crown Street, Liverpool L69 7ZD, UK; 2 Center for Neutron Research, National Institute of Standards and Technology, Gaithersburg, Maryland 20899, USA; 3 Defence Science and Technology Laboratory, Porton Down, Salisbury SP4 0JQ, UK; 4 School of Chemistry, University of Edinburgh, King's Buildings, David Brewster Road, Edinburgh EH9 3FJ, UK

## Abstract

Proton conduction is a fundamental process in biology and in devices such as proton exchange membrane fuel cells. To maximize proton conduction, three-dimensional conduction pathways are preferred over one-dimensional pathways, which prevent conduction in two dimensions. Many crystalline porous solids to date show one-dimensional proton conduction. Here we report porous molecular cages with proton conductivities (up to 10^−3^ S cm^−1^ at high relative humidity) that compete with extended metal-organic frameworks. The structure of the organic cage imposes a conduction pathway that is necessarily three-dimensional. The cage molecules also promote proton transfer by confining the water molecules while being sufficiently flexible to allow hydrogen bond reorganization. The proton conduction is explained at the molecular level through a combination of proton conductivity measurements, crystallography, molecular simulations and quasi-elastic neutron scattering. These results provide a starting point for high-temperature, anhydrous proton conductors through inclusion of guests other than water in the cage pores.

## Abstract (web summary)

Proton conduction is a fundamental process for fuel cell development, but three-dimensional proton conduction in crystalline porous solids is rare. Here, the authors report organic molecular cages in which the structure imposes three-dimensional proton conductivity competing with metal-organic frameworks.

## Graphical abstract

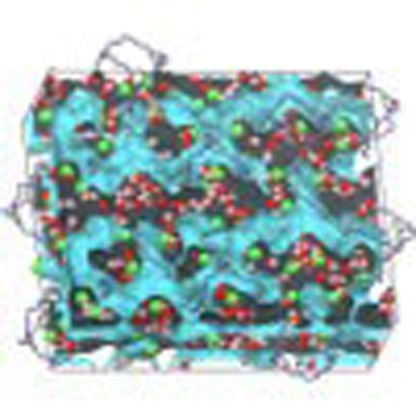

Proton exchange membrane fuel cells (PEMFCs) are an important clean energy platform. The performance-limiting component in PEMFCs is often the proton exchange membrane (PEM), which facilitates fast and selective proton transport^1^,^2^. The most common PEM materials are sulfonated fluoropolymers, such as Nafion^3^. Inspired by the need for more effective PEMs, the structural and chemical features that enhance proton conduction have been studied for wide range of materials^4^,^5^,^6^,^7^. Porous solids such as metal-organic frameworks (MOFs)^8^,^9^ or covalent organic frameworks^10^ have been a particular focus because the proton conduction properties can be fine-tuned by controlling crystallinity, porosity and chemical functionality. Unlike semi-crystalline or amorphous polymers, the well-defined pore networks in crystalline solids make them ideal as model compounds for the study of proton transport pathways and conduction mechanisms^9^,^11^. Porous organic molecules^12^,^13^ are an emerging class of porous solids that have unique properties, such as solution processability^14^,^15^,^16^. Like MOFs and covalent organic frameworks, the pore size and the pore topology can be precisely controlled. For example, porous organic cage molecules can be directed to adopt 3D pore topologies^17^,^18^, which therefore enhances mass transport properties.

In principle, the rational design of architecture in crystalline porous molecules allows us to tune proton conductivity and improve our understanding of proton conduction mechanisms, as relevant to both materials science and biology^19^. However, there are few examples of proton conduction in porous organic molecular solids. Kim *et al*.^20^ showed that the proton conductivity of cucurbituril-based materials is a result of an extensive hydrogen-bonding network formed by water and acid molecules in one-dimensional (1D) channels. This gave highly anisotropic conductivities of up to 4.3 × 10^−2^ S cm^−1^ along the 1D channel axis but only 5.0 × 10^−6^ S cm^−1^ perpendicular to this axis (98 % relative humidity (RH), 298 K). Müllen *et al*.^21^,^22^ studied a series of non-porous phosphonic acids, which were π-stacked into three-dimensional (3D) columns. These materials exhibited high proton conductivities of up to 2.5 × 10^−2^ S cm^−1^ (room temperature, 95% RH) in the case of hexakis(*p*-phosphonatophenyl)benzene.

One limitation of proton conduction in MOFs is the tendency for directional proton transport, which in turn arises from the low-dimension pore structures in most frameworks tested^23^,^24^. Even in the few 3D proton-conducting MOFs that are known, the protons were found to be transported in 1D channels in most cases^25^,^26^,^27^. 3D proton transport is more favourable for application in PEMs^28^,^29^, and hence there have been attempts to enhance proton mobility in MOFs by introducing defects or by decreasing the crystallinity^29^,^30^,^31^.

Here we present an alternative strategy, which is to develop crystalline porous molecular solids where the proton transport occurs in 3D pathway by virtue of the native channel structure and topology. We demonstrate this concept for a range of crystalline porous organic cages (Fig. 1). For a neutral imine cage, **CC3** (ref. 32) (Fig. 1a), the proton conductivity is relatively low under humid conditions, despite the hydrated 3D diamondoid pore network in the material (Fig. 1c). However, when a related amine cage, **RCC1** (ref. 33), (Fig. 1b) was transformed into its crystalline hydrated salt (H_12_**RCC1)**^12+^·12Cl^−^·4(H_2_O) (**1**, Fig. 1d), the proton conduction was improved by a factor of over 150. Indeed, the proton conductivity of **1** is comparable to pelletized proton-conducting MOFs^8^,^9^. This was rationalized using both computer simulations and quasi-elastic neutron scattering (QENS) to elucidate the proton transport mechanism. We also explain the influence of the counter anions in the protonated cage salts (Fig. 1b,d,e), which act to ‘gate' the proton conduction.

## Results

### Conductivity of CC3

**Figure 1:**
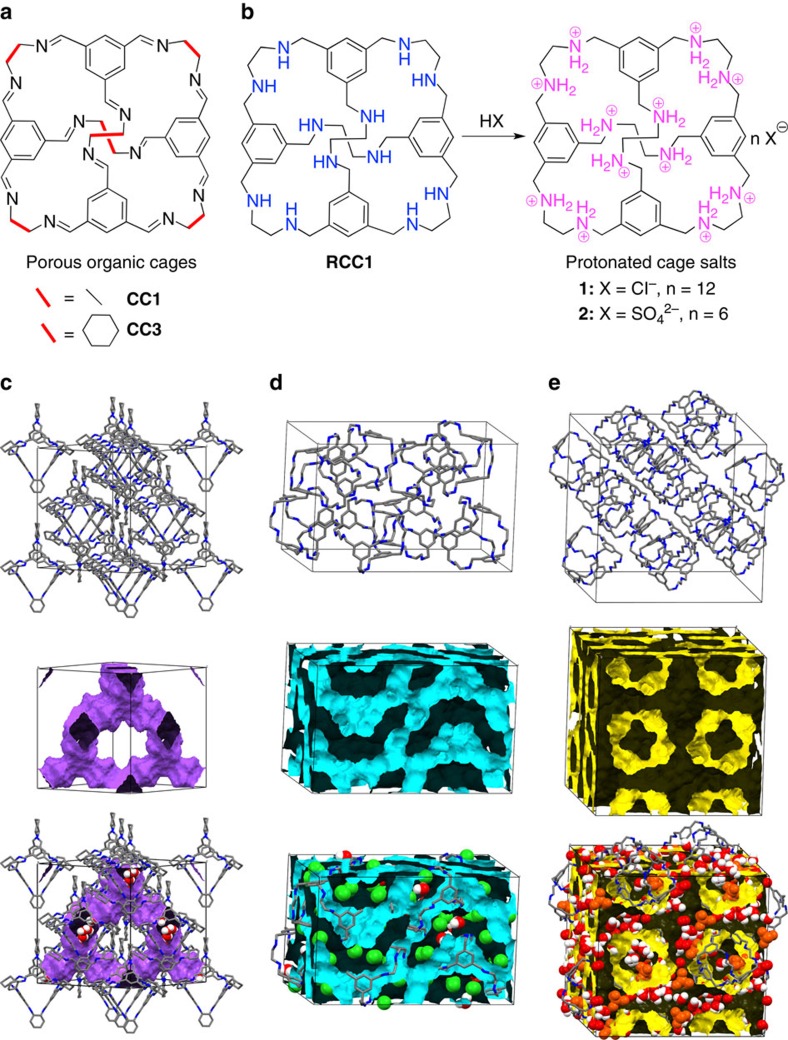
Molecular proton conductors based on neutral organic cage molecules and protonated cage salts. (**a**) Chemical structure of neutral porous organic cages **CC1** and **CC3**. (**b**) Preparation of cage salt materials (H_12_**RCC1**)^12+^·12Cl^−^ (**1**) and (H_12_**RCC1**)^12+^·6(SO_4_)^2−^ (**2**) by reaction of **RCC1** with mineral acids. (**c**) Hydrated 3D diamondoid pore network in crystalline **CC3**. (**d**,**e**) The 3D interconnected pores in **1** and **2**, respectively, have narrow bottlenecks and these pore channels are filled with H_2_O molecules and counter anions.

**Figure 2:**
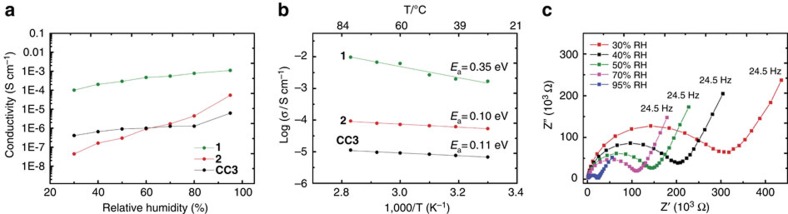
Proton conductivity and electrochemical data for porous organic cage materials. (**a**) Proton conductivities for salts **1** and **2**, and for neutral **CC3** at 303 K as a function of relative humidity. (**b**) Arrhenius plots showing the activation energies of the cage materials tested at 95% RH between 303–353 K. (**c**) Nyquist plots showing the impedance of **CC3** at 303 K with varying relative humidity (RH) between 1 MHz–24.5 Hz.

The neutral, crystalline cage solid **CC3** can reversibly adsorb up to 20.1 wt% water, which equates to approximately 12 H_2_O molecules per cage^34^. These H_2_O molecules can be located by single-crystal X-ray diffraction (SC-XRD; Fig. 1c), but their displacement parameters indicate that they are mobile in the 3D interconnected pore network, and hence could introduce proton conductivity, as for Nafion^11^ and water-mediated proton-conducting MOFs^8^,^9^. Conductivity measurements, using compacted pellets of powdered crystalline **CC3** at 303 K, revealed that the proton conductivity increased with RH in the range 30–95 % (Fig. 2c), with a maximum value of 6.4 × 10^−6^ S cm^−1^ at 95 % RH (Supplementary Figs 1–5). This is close to the proton conductivity of cucurbit[6]uril (CB[6]·H_2_O) under similar conditions (6.6 × 10^−6^ S cm^−1^)^20^, and ∼640 times higher than bulk water. The activation energy for **CC3** calculated from the Arrhenius plot at 98 % RH was 0.11 eV, which is lower than the cucurbituril material (0.31–0.56 eV)^20^. This low activation energy suggests a Grotthuss mechanism (activation energies 0.1–0.4 eV), where a hydronium ion reorients and passes its proton to a neighbouring water molecule through a hydrogen bond^1^. The relatively low activation energy can be explained by the confined environment imposed on the water arrays/chains^35^. Also, the 3D interconnected pores in **CC3** are beneficial for proton transport in comparison to the 1D proton transport pathways found in many MOFs.

### Structure and conductivity of 1

Encouraged by the proton conduction in neutral **CC3**, we investigated a series of protonated cages. Crystallization of an amine cage (**RCC1**; the reduced form of **CC1** (ref. 32), Fig. 1b) from dilute aqueous HCl solution afforded a cage salt, **1**. The solvated SC-XRD structure of **1** was refined with *P*4_1_ symmetry, with two (H_12_**RCC1)**^12+^ molecules in the asymmetric unit (Supplementary Data 1, Supplementary Figs 6 and 7). The 24 chloride anions are charge balanced by protonation of the 12 **RCC1** amine groups. In **1**, the (H_12_**RCC1)**^12+^ organic cations pack around fourfold screw axes parallel to the crystallographic *c* axis (Supplementary Fig. 7), and are held in this helical arrangement via a 3D hydrogen-bonded network with the chloride anions and the H_2_O molecules (Supplementary Figs 7b and d). Diffuse electron density in the (H_12_**RCC1)**^12+^ cage cavities was assigned as partially occupied H_2_O. There is no evidence of chloride anions occupying the cage cavities, which is central to the resulting proton conduction mechanism. A number of the chloride anions and H_2_O molecules were disordered over multiple positions and are clearly mobile in the structure, even at 100 K. PXRD data indicates that the same crystalline phase is retained after proton conductivity measurements (Supplementary Figs 8–10, Supplementary Table 1).

Cage salt **1** shows a high proton conductivity of ∼1.0 × 10^−4^ S cm^−1^ at low relative humidity (30 % RH; Fig. 2c), which is comparable to the performance of as-received Nafion (Sigma-Aldrich, Nafion 117; Supplementary Fig. 11). The conductivity of **1** gradually increases with RH, up to maximum value of 1.1 × 10^−3^ S cm^−1^ at 95% RH and 303 K (Supplementary Figs 12–20). This approaches the highest proton conductivities found in MOFs^8^. The Arrhenius plot at RH 95 % for **1** (Fig. 2b) yielded an activation energy of 0.35 eV.

### Atomistic simulations of proton transport in 1

**Figure 3:**
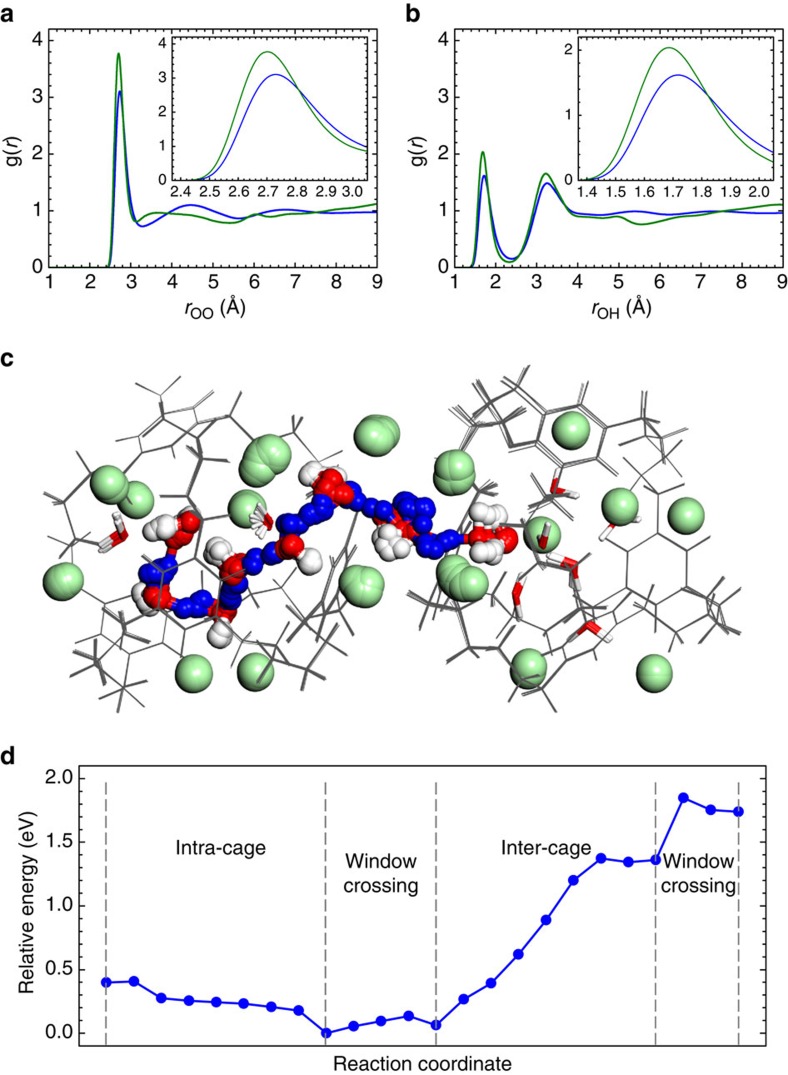
Atomistic simulations explaining the mechanism for proton transport in 1. (**a**,**b**) Radial distribution functions (RDFs) indicate that water molecules are confined in the cage solid; (**a**) oxygen–oxygen and (**b**) oxygen–hydrogen pairs between water molecules in **1** at 95% RH (green) and in bulk H_2_O at 1 bar (blue), as obtained from classical molecular dynamics simulations (298 K); the insets show a magnification of the first RDF peaks to show the shift that occurs when H_2_O is confined in **1**. (**c**,**d**) A minimum-energy pathway (MEP) for proton migration between two neighboring cages in **1**, simulated using first-principles density functional theory coupled with the climbing-image nudged elastic band (CI-NEB) method. (**c**) An overlay of all of the CI-NEB images (i.e., the various molecular configurations along the MEP); cage molecules are in grey, chloride ions in green, oxygen in red, and hydrogen in white or blue (the protons directly involved in the migration are coloured blue). (**d**) The potential energy profile for the MEP illustrated in **c**.

We used atomistic simulations to build a molecular level picture of the proton conduction mechanism in **1** and its structural analogues (Supplementary Figs 21–30). Broadly speaking, two environments exist in **1** that can accommodate water: the pores inside the cage molecules (the intrinsic pores) and the channels running in-between the cages (the extrinsic pores). The chloride ions, located just outside the cage window, form a gateway connecting these two kinds of pores. At 95% RH, molecular simulations suggest that water clusters are formed inside the cage cavities (consistent with X-ray data), while hydrogen-bonded chains of water molecules exist in the extrinsic pores (Supplementary Fig. 21). The water molecules adsorbed in **1** experience modest confinement compared to bulk water, leading to increased effective interactions between neighboring water molecules and moderately enhanced peaks in the radial distribution functions (Fig. 3a,b; Supplementary Fig. 22). Shortened H_2_O–H_2_O distances help to initiate fast intermolecular proton transfer events^36^.

The water molecules in neutral **CC3** are significantly more structured than those in **1** or in bulk water (Supplementary Fig. 23). Although strong hydrogen bonds favour fast intermolecular proton transfer, hydrogen-bond reorganization also requires bond breaking and bond forming, and it is often the rate-limiting step in the Grotthuss mechanism. This reorganization can be suppressed by the reduced dynamics in highly structured water, hence reducing the long-range mobility of protons. We propose that water structuring explains why **CC3** shows only a modest improvement in proton conductivity over bulk water, and a much lower conductivity than **1**. A well-balanced combination of order and disorder^24^, allowing both fast intermolecular proton hopping and easy solvent reorganization, is desirable for high proton conduction.

A simulation of the proton migration in **1** is shown in Fig. 3c,d, performed using first-principles density functional theory coupled with the climbing-image nudged elastic band method^37^. Proton transfer through the water cluster confined inside a cage cavity proceeds via Grotthuss diffusion in a barrier-less manner (Fig. 3d). The cage molecules play an important role in promoting fast intra-cage proton transfer. The cages confine the water, which promotes fast migration of protons. However, the cages are also intrinsically flexible, allowing facile hydrogen-bond reorganization, which is pivotal for facilitating long-range proton migration. Hence, this material achieves the benefits of ‘soft confinement' without unduly constraining hydrogen bond reorganization. The simulations also suggest that protons cross a cage window by hopping between the water molecules at the two sides of the window, associated with small energy barriers (*ca.* 0.2 eV, Fig. 3d).

Proton transport in the extrinsic void space in **1** should vary with the level of hydration, since low extrinsic water content leads to hydrogen-bond networks that are not formally interconnected. In such cases, translational diffusion of aqueous cations (for example, H_3_O^+^, H_5_O_2_^+^ and so on) is required to advance long-range proton migration. Indeed, diffusion of a hydronium ion over a short distance in the extrinsic void was observed along the MEP shown in Fig. 3c (Supplementary Fig. 26). This resembles the vehicular mechanism and is characterized by an energy barrier of 1.0 eV in the MEP (Fig. 3d). Diffusion of the larger Zundel and Eigen cations was not observed, consistent with the small dimensions of the extrinsic pores in **1**.

### Structure and conductivity of 2

**Figure 4:**
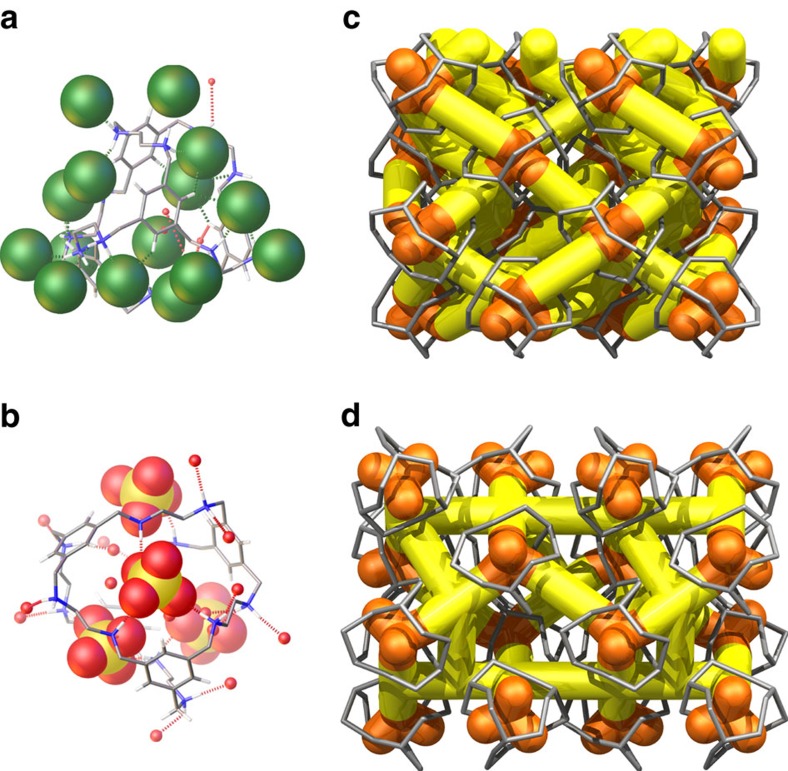
The single-crystal structures of cage salts 1 and 2 showing 3D channel structures. The H_12_RCC1^12+^ cage molecules are surrounded by Cl^−^ anions (green space-filling representation) and H_2_O molecules (red spheres) in **1** (**a**), and (SO_4_)^2−^ anions (yellow and red space-filling representation) and H_2_O molecules (red spheres) in **2** (**b**). Graphical representation of interconnected 3D networks of hydrogen-bonded anions, and H_2_O molecules in **1** and **2**: These 3D networks pass though the intrinsic cage cavities (orange) and the extrinsic voids between the cages (yellow), shown for a 4 × 4 cage array (cages in grey; anions omitted) in **1** (**c**), and **2** (**d**).

To investigate the influence of the anion in **1**, tetrahedral (SO_4_)^2−^ anions were introduced with a much larger radius than the spherical chloride anions (2.90 versus 1.67 Å). Crystallization of **RCC1** from dilute H_2_SO_4_ (aq.) afforded salt **2**. The SC-XRD structure of **2** was refined with *Fdd*2 symmetry as (H_12_**RCC1)**^12+^·6(SO_4_)^2−^·27.25(H_2_O) (Supplementary Data 2). Sulfate anions occupy the cage windows, and to some extent the cage cavity, and 4–5 ordered H_2_O molecules were located in the intrinsic cage cavity. The (SO_4_)^2−^ anions and H_2_O form a 3D hydrogen-bonded network (Fig. 4), and the flexible cage windows hydrogen bond to the sulfate anions, significantly altering the conformation adopted by the (H_12_**RCC1)**^12+^ molecule (Supplementary Figs 31–33). The water molecules in **2** were well resolved in the structures measured at 100 K and at 293 K (Supplementary Data 3), while in **1**, the water positions were poorly resolved, even at 100 K, suggesting that water is more dynamic in **1** than in **2**.

Unlike **1**, the crystal structure of **2** transforms on changing temperature or water content (Supplementary Figs 34–44). PXRD indicates that the single-crystal structure is representative of the fully hydrated bulk material at 295 K (Supplementary Figs 34 and 35). A closely related structure, likely to be formed as a result of some water loss, is observed for samples of **2** prepared for proton conductivity measurements (Supplementary Figs 42 and 43). This phase is stable at the temperature at which we performed the variable humidity conductivity measurements (Supplementary Table 2 and Supplementary Figs 42–44), and the structure of the pellet is unchanged from the original phase after conductivity measurements (Supplementary Figs 45 and 46).

The measured proton conductivity of **2** also increases with RH in the range 30–95% RH (Supplementary Figs 47–49). However, at RH 30% (303.15 K), the conductivity of **2** was only 3.2 × 10^−8^ S cm^−1^, which is more than 3,000 times lower than **1** under the same conditions. The conductivity for **2** increased rapidly to 6.1 × 10^−5^ S cm^−1^ at 95% RH, but this is still about 20 times lower than for **1** under the same conditions. The conductivity for **2** over this humidity range rises by almost a factor of 2,000, while the equivalent increase for **1** is only a factor of 10, suggesting a more pronounced effect of change in water content with humidity for **2** in comparison with **1** (see further discussion below). On the other hand, the activation energy determined for **2** from the Arrhenius plot (0.10 eV; Fig. 2b) is lower than for **1** and close to the value of neutral **CC3**.

**Figure 5:**
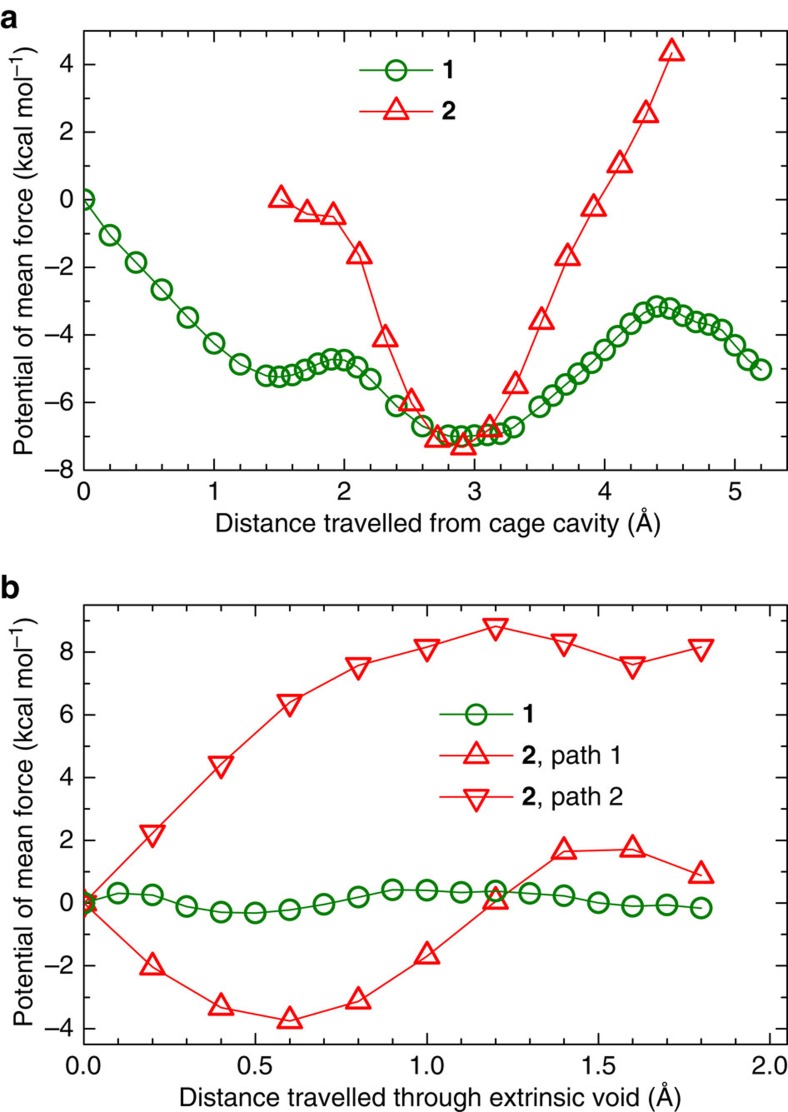
Potential of mean force profiles of a water molecule diffusing in 1 and 2. The potential of mean force (PMF) profiles were plotted as a function of the reaction coordinate, which is the distance between the centre of mass (COM) of the water molecule and (**a**) the COM of the cage or (**b**) the COM of a cage window; the PMF at the starting position was arbitrarily set to zero. The results were obtained using solid-state classical molecular simulations.

Potential of mean force calculations for a single water molecule diffusing in solid-state **1** and **2** revealed that the water dynamics are markedly different in the two structures. In both **1** and **2**, it is energetically favourable for the water molecule in the intrinsic void to move toward a cage window, owing to the strong attractions with the anions (Cl^−^ or (SO_4_)^2−^) sitting at the window. However, it is considerably more difficult for this water molecule to traverse the window in **2** than in **1**; the window-crossing event corresponds to the reaction coordinate varying between *ca.* 3.5 Å and *ca.* 4.5 Å (Fig. 5a). This is because the cage windows in **1** are gated by the smaller, monovalent Cl^−^ ions, while the windows in **2** are gated by the larger, divalent (SO_4_)^2−^ ions. Similarly, the diffusion of water in the extrinsic voids requires significantly larger activation in **2** than in **1** (Fig. 5b). These differences in water mobility are consistent with the relative order of the water molecules in the crystal structures of **1** and **2**: the water positions are well resolved in **2** at 293 K, but are poorly resolved for **1**, even at 100 K.

## Discussion

These simulations rationalize the different proton conductivities measured for **1**, **2** and **CC3**. At low humidity levels, all three materials are poorly hydrated. The adsorbed water molecules in **2** are locally organized around the doubly-charged (SO_4_)^2−^ ions, leading to considerably restricted diffusive motions of water. This explains the higher proton conductivities observed for neutral **CC3** up to 60% RH, which does not impose similar restrictions on the translational diffusion of water. The increase in conductivity with relative humidity is most significant for **2** (Fig. 2a), where the undesirable localization of adsorbed water at low hydration levels is increasingly compensated by the extended hydrogen-bond network that is formed. In keeping with this, the activation energy for proton transfer in **2** at 95% RH is low (0.10 eV, Fig. 2b), indicating that Grotthuss diffusion is the predominant mechanism. The higher activation energy calculated for **1** suggests that a degree of translational diffusion of proton carriers (for example H_3_O^+^) is required to facilitate long-range proton conduction.

Unlike **CC3**, both **1** and **2** have strong ionic character, and the anions are pivotal in maintaining the crystal packing and in facilitating proton conduction. Both Cl^−^ and (SO_4_)^2−^ ions are powerful hydrogen-bond acceptors, and acidic protons and proton holes (OH^−^) can be generated through dissociation of H_2_O when these anions are hydrated. Even without the dissociation of H_2_O, the elongated O–H bond in the O_water_–H_water_···anion hydrogen-bond complex will free up the oxygen atom of H_2_O to accept extra protons. Hence, the incorporation of charged ions into otherwise neutral porous cages increases the concentration of protons and/or proton carriers, thus increasing the protonic conductivity.

**Figure 6:**
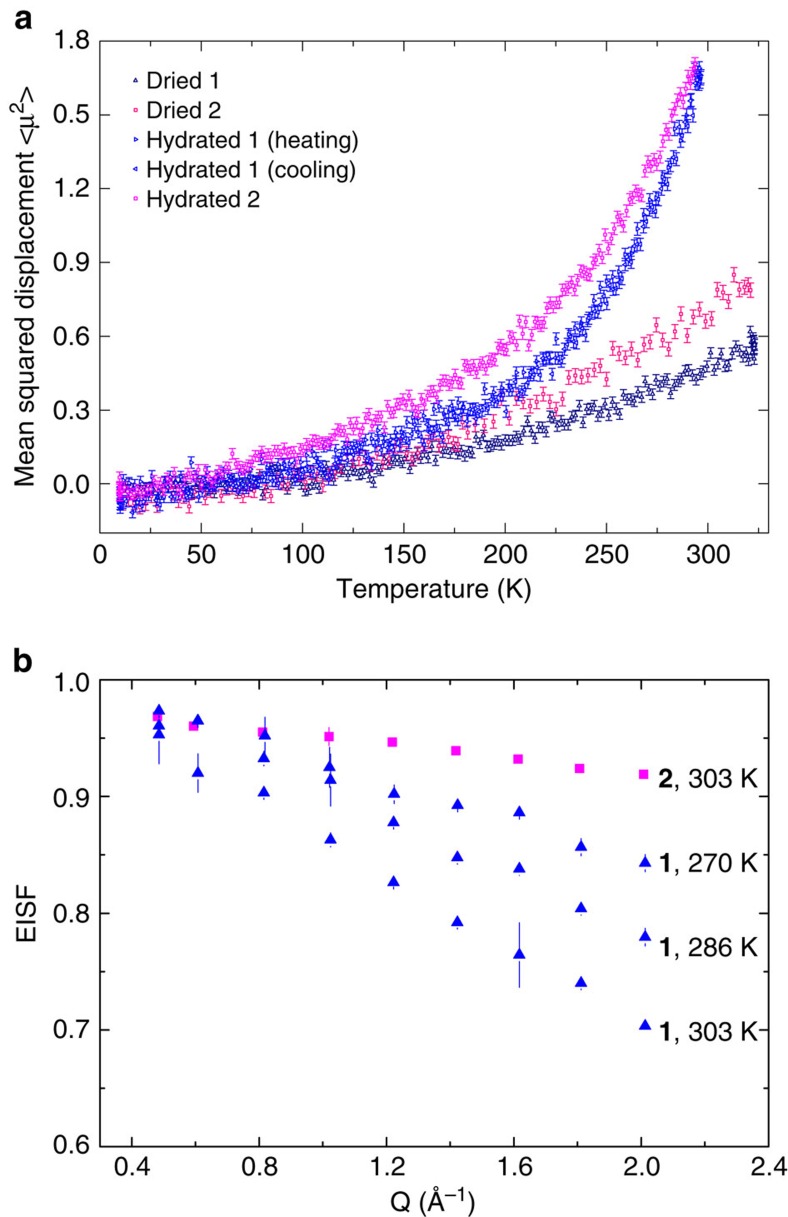
Quasi-elastic neutron scattering measurements for cage salts 1 and 2. (**a**) Mean squared displacements with associated experimental error bars derived from the temperature-dependent normalized elastic scattering intensities of **1** and **2** from fixed window scan using the high-flux backscattering spectrometer (HFBS). (**b**) Elastic incoherent structure factor (EISF) of hydrated **1** (270, 286 and 303 K) and hydrated **2** (303 K) from the data measured on the Disk Chopper Spectrometer (DCS). Error bars indicate uncertainties derived from fitting the elastic and inelastic contributions to the experimental QENS intensities.

QENS can probe the dynamics of bulk water and confined water^38^,^39^ and provide experimental support for proton transport mechanisms proposed by simulations (Supplementary Figs 50–58). Fixed window scans collected on the High-Flux Backscattering (HFBS) instrument at the NIST Center for Neutron Research (Fig. 6a) indicate the temperature at which proton diffusive motions in the structure matches the timescale that can be measured by the instrument. The elastic scattering of the dried samples of **1** and **2** showed a near-linear temperature dependence (10–323 K), indicating that the movement of protons in the system remains essentially harmonic throughout. The hydrated samples of **1** and **2** show an increase in displacement at ∼200 K, which relates to the onset of diffusive motions; that is, rotation or translation of water molecules in the structure above this temperature. However, no significant quasi-elastic scattering was observed using HFBS between 200 and 303 K, possibly because the dynamics in these samples are too rapid for the instrument to measure (HFBS timescale 10^−9^–10^−8^ s).

By contrast, data collected on the Disk Chopper Spectrometer (DCS; timescale 10^−12^–10^−10^ s) shows quasi-elastic scattering at temperatures above 220 K that is distinguishable from the resolution function of the instrument measured at 50 K (Supplementary Fig. 50). The Elastic Incoherent Structure Factor derived from the Q-dependent spectra of hydrated **1** and **2** (Fig. 6b, Supplementary Fig. 56) shows that the quasi-elastic scattering in hydrated **1** is more pronounced than for hydrated **2**. This implies that a significant number of protons are more mobile in **1**, which is consistent with the more disordered water molecules in the crystal structure of **1**. The use of two Lorentzian functions (narrow and broad) significantly improved the fit of spectra at T≥270, which is indicative of at least two diffusive behaviours in the system. The extracted line widths of the narrow function (the Lorentzian HWHM, Γ(Q)) did not show pronounced Q^2^-dependence (Supplementary Fig. 53). This is characteristic of proton motions of a localized nature, which is generally related to the Grotthuss mechanism involving only reorientation of hydronium ions^38^. On the other hand, for the broad Lorentzian component (Supplementary Fig. 54), the HWHM at low Q^2^ shows an approximately linear trend following Fick's law. Departure from Fickian behaviour was observed at higher Q^2^, suggesting a jump diffusion process^40^, consistent with the vehicle mechanism for the proton transport between two neighboring cages proposed by our simulations. The co-existence of two mechanisms of proton conduction in **1**, inferred from the activation energy and suggested independently by computational simulations, is thus supported by these QENS data.

In summary, porous organic cages show potential as proton-conducting materials with figures of merit that compete with more widely-studied porous solids, such as MOFs. Unlike MOFs, however, these molecular cages can be processed as solutions in certain organic solvents, which might give advantages in terms of device fabrication for PEMFCs—for example, to prepare thin films^14^,^15^ or composite materials such as Nafion membranes containing molecular cage additives.^14^ The 3D interconnected pore network in cage salt **1** will not restrict protons to diffuse directionally, which has been rarely seen in extended framework materials. Moreover, the ‘soft confinement' benefits observed in **1** may be a more general feature of porous molecular cages, which tend to be quite flexible^41^. Our first study focuses on hydrated materials, but given the large number of small molecule guests that can be accommodated in molecular cages^42^,^43^,^44^, then porous molecular solids should also be useful for anhydrous proton conduction at higher temperatures. For example, cage hosts might be used to direct secondary organic proton carriers into 3D proton conduction topologies.

## Methods

### Synthesis of 1

**RCC1** (500 mg, 0.612 mmol) was dissolved in CHCl_3_ (10 ml) by stirring. Hydrogen chloride (in dioxane, 2.30 ml, 9.18 mmol) was added dropwise. White precipitate appeared and the reaction mixture was stirred for a further 2 h at room temperature. The precipitate was collected by filtration then washed by CHCl_3_ (3 × 20 ml). **1** (crude yield=550 mg, 71.6 %) was obtained as a white solid after being dried under vacuum at 90 °C. mp: decomposes > 220 °C; ^1^H NMR (400 MHz, D_2_O) δ 7.68 (s, 12H, -ArH), 4.41 (s, 24H, -ArCH_2_), 3.52 p.p.m. (s, 24H, −NCH_2_); ^13^C NMR (100 MHz, CDCl_3_): δ 132.7, 132.1, 50.6, 42.8 p.p.m. HRMS (ES/APC+) calc. for **RCC1**, C_48_H_72_N_12_ [M+H]^+^ 817.6076, found 817.6076. Elemental analysis calcd (%) for (H_12_**RCC1**)^12+^·12Cl^−^·4H_2_O : C 43.45, H 6.99, N 12.67, Cl 32.07; found: C 43.10, H 6.85, N 12.47, Cl 31.90. IR (KBr pellet, ν) 3379 (m), 2955 (m), 2737 (s), 2420 (w), 1582 (w), 1445 (s), 1180 (m), 1032 (m), 893 (m), 779 (m), 712 (m), 509 (m) cm^−1^.

### Synthesis of 2

H_2_SO_4_ aqueous solution (1 M, 1.46 ml) was added to **RCC1** (200 mg, 0.245 mmol) in H_2_O (5 ml) with stirring. White precipitate appears and the reaction mixture was stirred for a further 1 hour at room temperature. The precipitate was collected by filtration and recrystallized in H_2_O. **2** (crude yield=302 mg, 87.7%) was obtained as a colourless block crystals. mp: decomposes >210 °C; ^1^H NMR (400 MHz, D_2_O,) δ 7.70 (s, 12H, −ArH), 4.35 (s, 24H, −ArCH_2_), 3.50 (s, 24H, -NCH_2_) ppm; ^13^C NMR (100 MHz, D_2_O): δ 133.5, 131.8, 51.0, 43.8 p.p.m. HRMS (ES/APC+) calc. for **RCC1**, C_48_H_72_N_12_ [M+H]^+^ 817.6076, found 817.6057. Elemental analysis calculated (%) for (H_12_**RCC1**)^12+^)·6(SO_4_)^2−^·21.5H_2_O: C 32.15, H 7.14, N 9.37, S 10.73; found: C 32.14, H 6.83, N 9.36, S 10.59. IR (KBr pellet, ν) 3348 (w), 2987 (m), 2667 (w), 2453 (w), 1616 (m), 1464 (w), 1041 (s), 970 (w), 789 (w), 719 (w), 608 (s) cm^−1^.

For ^1^H NMR, ^13^C NMR spectra, TGA plots, water isotherms and scanning electron microscopy images of compounds **1** and **2** (Supplementary Figs 59–66). For the general information of materials and the analytical methods, please see Supplementary Methods.

### Impedance spectroscopy

For proton conduction measurements, Samples were weighed using an analytical balance and subsequently ground to a fine powder using a pestle and mortar. The pellets were dried overnight under vacuum at 363.15 K.

A T-shaped Teflon Swagelok cell was assembled sandwiching the pellets between two platinum foil (blocking electrodes). The assembled Swagelok cell was connected to an EC Labs Biologic VMP3 potentiostat using banana plug cables. 2 probe (quasi four probe) electrochemical impedance spectroscopy was measured using a sinusoidal perturbation of 100 mV over the frequency range 100–1 MHz. To investigate the effect of humidification and temperature, a Memmertt Celsius humidity chamber was used. Impedance measurements were taken between 30–95% relative humidity and 303–383 K. For the humidity investigation, an equilibration time of 4 h was required between taking measurements in order for water sorption to stabilize.

### Single-crystal X-ray diffraction

Single-crystal X-ray data for (H_12_**RCC1**)^12+^·12Cl^−^·4(H_2_O) (**1**) was measured at beamline I19, Diamond Light Source, Didcot, UK using silicon double crystal monochromated radiation (*λ*=0.6889 Å)^45^. Single-crystal X-ray data sets for (H_12_**RCC1**)^12+^·6(SO_4_)^2−^ (**2**) were measured on a Rigaku MicroMax-007 HF rotating anode diffractometer (Mo-Kα radiation, λ=0.71073 Å, Kappa 4-circle goniometer, Rigaku Saturn724+ detector). Empirical absorption corrections using equivalent reflections were performed with the program SADABS^46^. Structures were solved with SHELXD^47^, or by direct methods using SHELXS^47^, and reined by full-matrix least squares on |*F|*^2^ by SHELXL^45^, interfaced through the programme OLEX2 (ref. 48). Unless stated, all non-H atoms were refined anisotropically and H atoms were fixed in geometrically estimated positions refined using the riding model.

Crystal data for (H_12_**RCC1)**^12+^·12Cl^−^·4(H_2_O) (**1**); CCDC entry 1,452,674. Formula C_48_H_90_N_12_Cl_12_O_4_; *M*=1,324.73 g mol^−1^; tetragonal space group *P*4_1_, colourless crystal; *a*=20.153(6) Å, *c*=31.892(9) Å; *V*=12952(8) Å^3^; *ρ*=1.359 g cm^−3^; *μ*=0.509 mm^−3^; *F* (000)=5,584; crystal size=0.21 × 0.20 × 0.17 mm; *T*=100(2) K; 182,229 reflections measured (0.62<*θ*<24.84°), 24,589 unique (*R*_int_=0.0613), 23,143 (*I* > 2σ(*I*)); *R*_1_=0.0728 for observed and *R*_1_=0.0774 for all reflections; w*R*_2_=0.1872 for all reflections; max/min difference electron density=1.507 and −0.401 ėÅ^−3^; data/restraints/parameters=24,589/85/1,469; GOF=1.091. Flack parameter 0.23(2). The structure was refined with the twin law 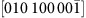 and the BASF parameter refined to 0.496(2).

Crystal data for (H_12_**RCC1)**^12+^·6(SO_4_)^2−^·27.25(H_2_O) (**2**); CCDC entry 1,452,672. Formula C_48_H_138.50_N_12_O_51.25_S_6_; *M*=1,896.56 g mol^−1^; orthorhombic space group *F*dd2, colourless crystal; *a*=32.757(2) Å, *b*=34.249(2) Å, *c*=32.016(3) Å; *V*=34877(4) Å^3^; *ρ*=1.445 g cm^−3^; *μ*=0.263 mm^−3^; *F* (000)=16264; crystal size=0.17 × 0.13 × 0.12 mm; *T*=100(2) K; 117,747 reflections measured (1.999<*θ*<29.128°), 23450 unique (*R*_int_=0.0600), 22,347 (*I*>2σ(*I*)); *R*_1_=0.0660 for observed and *R*_1_=0.0684 for all reflections; *wR*_2_=0.1854 for all reflections; max/min difference electron density=1.231 and −0.626 ėÅ^−3^; data/restraints/parameters=23,450/131/1,302; GOF=1.040. Flack parameter 0.115(14).

### Computer simulations

Proton mobility in **1** was investigated computationally by means of first-principles density functional theory (DFT), combined with the climbing-image nudged elastic band method^37^, using the CP2K package (https://www.cp2k.org). All DFT calculations made use of the Becke–Lee–Yang–Parr (BLYP)^49^,^50^ exchange–correlation functional with semi-empirical dispersion corrections to the energies and gradients from the DFT-D3 method^51^. The combination of BLYP and a correction for dispersion offers a satisfactory model for describing the density, structure and dynamics of water^52^. The MOLOPT basis sets of the double-ζ quality were used^53^, together with the Goedecker–Teter–Hutter pseudopotentials^54^,^55^; the charge-density cutoff for the auxiliary plane-wave expansions was set to 350 Ry. During each SCF cycle, the electronic structure was explicitly minimized to a tolerance of 10^−7^ Hartree. To probe proton transfer in **1** under aqueous conditions, we first identified thermodynamically favourable adsorption sites for water with the aid of classical simulations. Based on snapshots thus generated for 95 % RH at 298.15 K, climbing-image nudged elastic band calculations were then performed to identify and characterize minimum-energy pathways connecting possible proton sites. Classical, force-field-based molecular dynamics and Monte Carlo simulations were used to study the dynamics of water in **1**, **2**, **CC3**, and bulk; computational details are presented in the Supplementary Note 1.

### Powder X-ray diffraction

PXRD data were collected in transmission mode on loose powder samples held on thin Mylar film in aluminium well plates on a Panalytical X'Pert PRO MPD equipped with a high throughput screening (HTS) XYZ stage, X-ray focusing mirror and PIXcel detector, using Cu *Kα* radiation. Data were measured over the range 4–50° in ∼0.013° steps over 60 min. Laboratory PXRD data were collected from samples contained in borosilicate glass capillaries in transmission geometry on a Panalytical Empyrean diffractometer producing Cu *Kα* radiation and equipped with an X-ray focussing mirror. Data were collected using a PIXcel 3D detector in 1D scanning mode. For variable temperature PXRD measurements, the temperature of the capillary was controlled using an Oxford Cryosystems 700 Series Cryostream Plus. Patterns were indexed and lattice parameters extracted by Le Bail fitting in TOPAS Academic^56^.

### QENS study

The neutron scattering data was collected at the Center for Neutron Research (NCNR) of the National Institute of Standards and Technology (NIST, USA), using HFBS and DCS. For both HFBS and DCS, samples of hydrated **1**, and **2** were placed in an aluminium foil pouch (of thickness sufficient to maintain a 10 % scatterer) and rolled in to an annulus and placed inside an aluminium cell filled with helium and sealed. Temperature was maintained inside a closed-cycle refrigerator equipped with a Lakeshore temperature controller to better than 0.2 K variation over time. QENS measurements using an instrument configured for the highest neutron flux at a wavelength of 5.0 Å, with detectors masked that contained Bragg peaks, and grouped in momentum transfer (*Q*) with 0.2 Å^−1^ bins, allows for an accessible *Q* range of 0.27 Å^−1^ to 2.27 Å^−1^ with an elastic energy resolution of approximately 110 meV. The *Q*-dependent spectra collected with a wavelength of 6 Å (Supplementary Fig. 51) were fitted using Dave^57^ to a phenomenological proton diffusion model giving rise to a Lorentzian function and an elastic delta function all convoluted with the resolution function.

### Data availability

The X-ray crystallographic data for the structures reported in this Article have been deposited at the Cambridge Crystallographic Data Centre, under deposition numbers 1452672–1452674. These data files can be obtained free of charge via www.ccdc.cam.ac.uk/data_request/cif. Supplementary data files that support the findings of this study are available from the corresponding author on request.

## Additional information

**How to cite this article:** Liu, M. *et al*. Three-dimensional protonic conductivity in porous organic cage solids. *Nat. Commun.* 7:12750 doi: 10.1038/ncomms12750 (2016).

## Supplementary Material

Supplementary Figures, Tables, Note, Methods and References Supplementary Figures 1-66, Supplementary Tables 1 & 2, Supplementary Note 1, Supplementary Methods and Supplementary References.

Supplementary Data 1 Crystallographic information for 1 at 100 K

Supplementary Data 2 Crystallographic information for 2 at 100 K

Supplementary Data 3 Crystallographic information for 2 at 293 K
