# Supplementary material for: Three-dimensional protonic conductivity in porous organic cage solids
Source: Nat Commun. 2016 Sep 13;7:12750. doi: 10.1038/ncomms12750 (PMC5027280; doi:10.1038/ncomms12750)
Supplement: Supplementary Figures, Tables, Note, Methods and References — Supplementary Figures 1-66, Supplementary Tables 1 & 2, Supplementary Note 1, Supplementary Methods and Supplementary References. [file ncomms12750-s1.pdf]

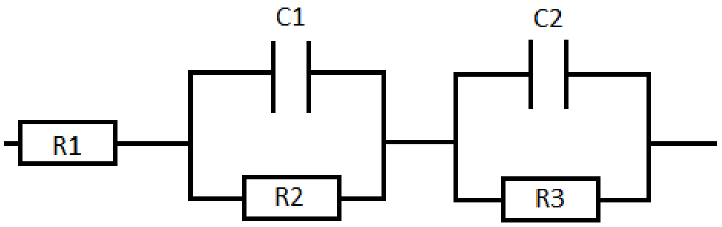

Supplementary Figure 1. Equivalent circuit model used to fit the impedance data in this study.

R1 is the contact resistance between the sample pellet and the electrode, R2 is the bulk resistance of the sample and R3 is the resistance attributed to grain boundary effects and occurs at low frequencies. C1 and C2 are capacitive processes and each RC parallel circuit corresponds to two semi-circular arcs in the Nyquist plots. The impedance for a given frequency for this circuit model is given by:

$$Z(f) = R1 + \frac{R2}{1 + j2\pi f R2C2} + \frac{R3}{1 + j2\pi f R3C3} \qquad (1)$$

The impedance spectra show a high frequency arc corresponding to the bulk (electrolyte resistance). Extrapolation of the arc to the X-axis gives the charge transfer resistance which is used to calculate the ionic conductivity using the formula $\sigma = \frac{l}{RA}$ where σ is the ionic conductivity (S cm^{-1}), l is the distance between the electrodes (cm) and A is the cross sectional area of the sample pellet (cm^2). Measurement in the direction of thickness leads to a smaller cell constant than in the plane but accounts for greater accuracy due to the anisotropy of the cage structure. The low frequency tail is largely capacitive, due to the accumulation of charge on the electrodes as a result of the polarisation potential. Analysis of the Bode plots confirmed this was not a diffusive process, which would correspond to a Warburg type impedance element. Instead, the model circuit can be modelled as two Randles circuits in series with two resistors and capacitors in parallel.

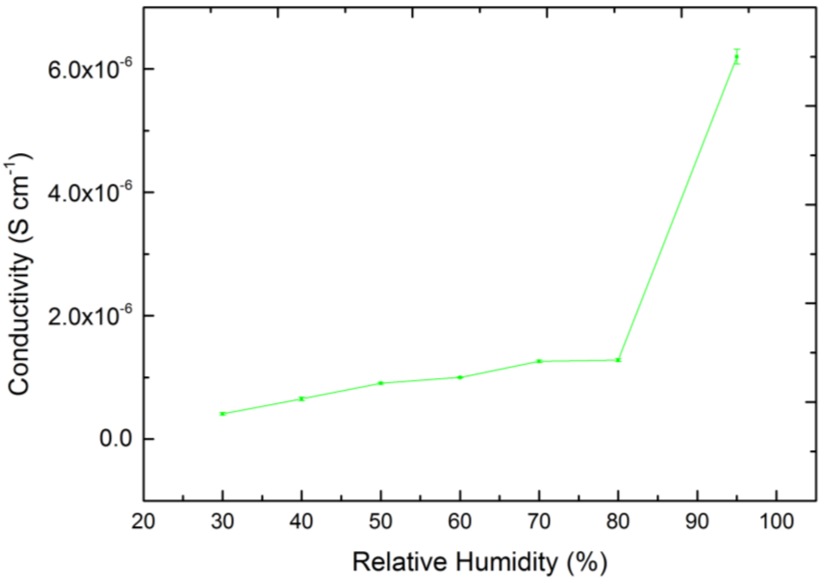

Supplementary Figure 2. Proton conductivity of **CC3** at 303 K subjected to variable relative humidity. The error bars shown represent the value of ±1 standard error of the mean of four consecutive samples that were tested, which are defined as $\sigma/\sqrt{n}$, where σ = standard deviation and n = number of samples tested.

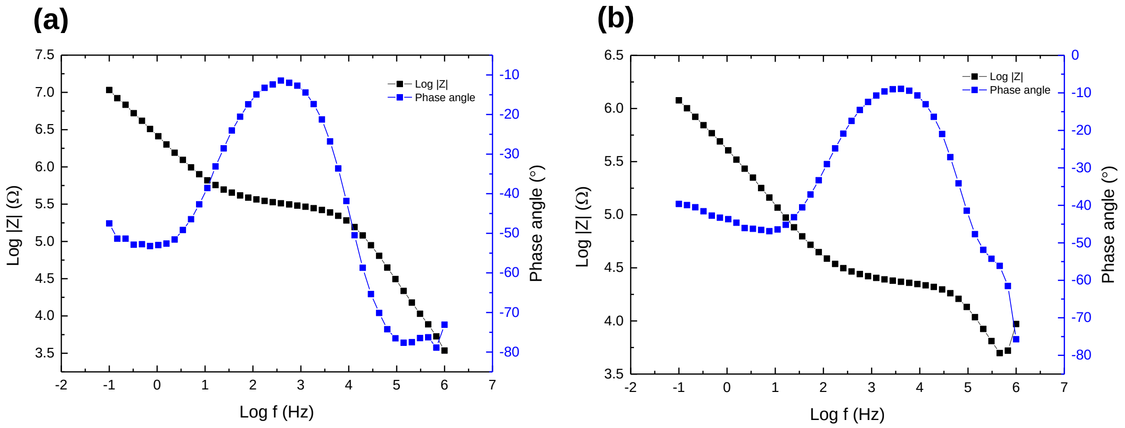

Supplementary Figure 3. Bode plots showing magnitude of impedance |Z| and phase angle as a function of frequency for **CC3** at 50 % RH (a) and 95 % RH (b). The total impedance plot is characteristic of resistive (slope=0) and capacitive (slope = −1), where the switching point is equal to 1/RC (RC being the time constant of the circuit). Where the phase angle approaches −45 ° this indicates predominately capacitive behaviour. Impedance was measured between 1 MHz and 0.1 Hz.

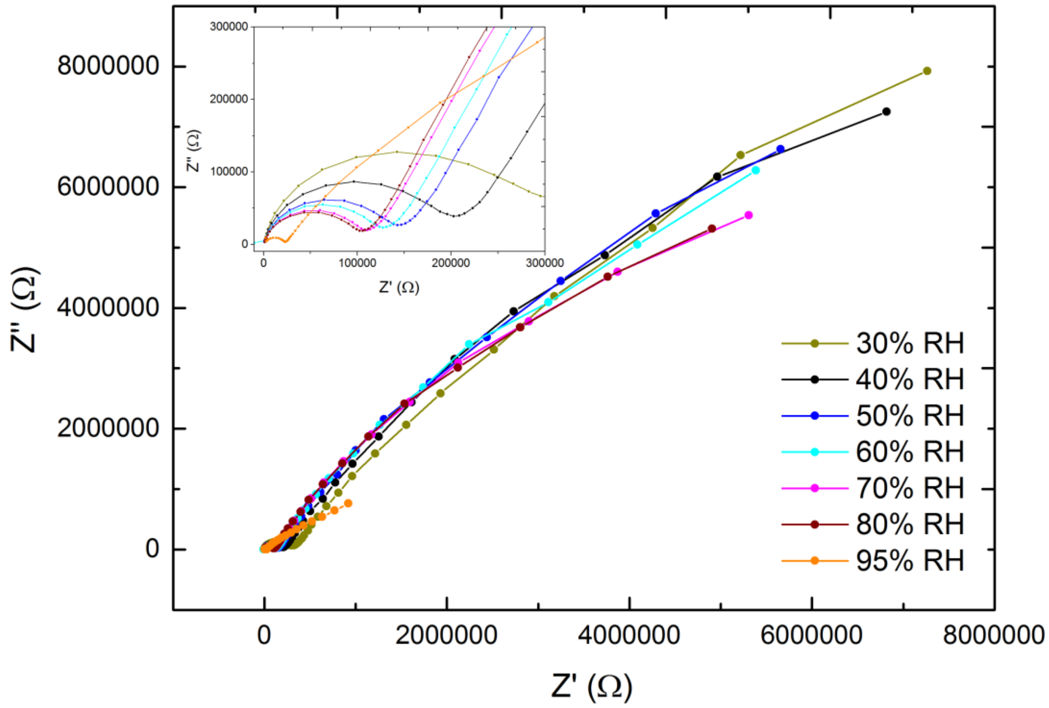

Supplementary Figure 4. Nyquist plots for **CC3** at 303 K with variable relative humidity between 1 MHz and 0.1 Hz.

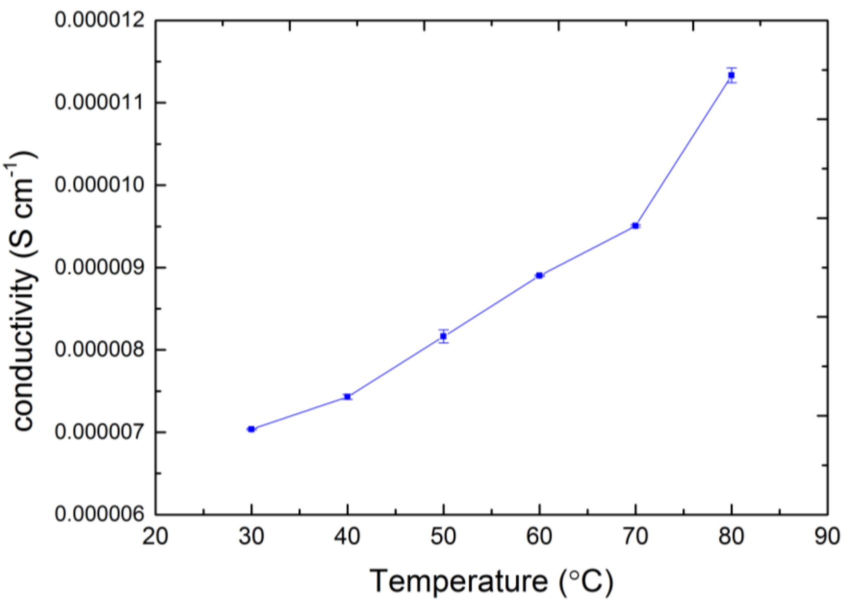

Supplementary Figure 5. Conductivity of **CC3** subjected to variable temperature, 95 % RH. The error bars shown represent the value of ±1 standard error of the mean of four consecutive samples that were tested, which are defined as $\sigma/\sqrt{n}$, where σ = standard deviation and n = number of samples tested.

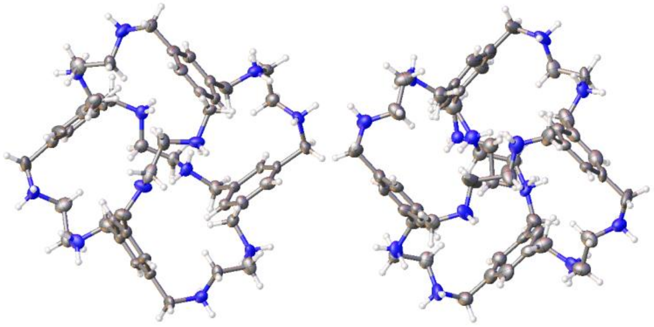

Supplementary Figure 6. Displacement elliposoid plot from the single crystal structure (H_{12}**RCC1**)$^{12+}$·12Cl^-·4(H_2O), chloride anions and H_2O molecules omitted for clarity. Ellipsoids displayed at 50 % probability level.

The asymmetric unit for (H_{12}**RCC1)**$^{12+}$·12Cl^-·4(H_2O) comprises two complete (H_{12}**RCC1)**$^{12+}$ molecules; 24 chloride anions modelled over 46 positions; and 8 H_2O molecules modelled over 14 positions. (H_{12}**RCC1)**$^{12+}$·12Cl^-·4(H_2O). The structure was refined with the twin law [010 100 00$\bar{1}$] and the BASF parameter refined to 0.496(2). One ethyl vertex was modelled over two positions, due to disorder both parts were refined isotropically (checkCIF PLAT201_ALERT_2_B alert), and bond length restraints were used during refinement (DFIX and SADI in SHELX). One of the disordered groups was refined with constrained displacement parameters (EADP). Two additional cage vertices were refined with restrained atomic displacement parameters (SIMU and DELU in SHELX). Disordered chloride anions, that were partially occupied, were refined isotropically. Disorder in the crystal structure resulted in low precision on C-C bonds (checkCIF PLAT340_ALERT_3_B). It was not possible to accurately locate H-atoms for the H_2O molecules in the crystal structure (checkCIF PLAT306_ALERT_2_B). H-atoms for these H_2O molecules were included in the unit cell atom count. For a displacement ellipsoid plot of the asymmetric unit from the single crystal structure, see Supplementary Fig. 7.

It was possible to accurately refine the single crystal structure, (H_{12}**RCC1**)$^{12+}$·12Cl^-, in enantiomorphic tetragonal space groups $P4_1$ and $P4_3$. Final refinements of (H_{12}**RCC1**)$^{12+}$·12Cl^-·4(H_2O) were carried out using the space group setting $P4_1$ with the twin law [010 100 00$\bar{1}$] and BASF parameter refined to 0.496(2).

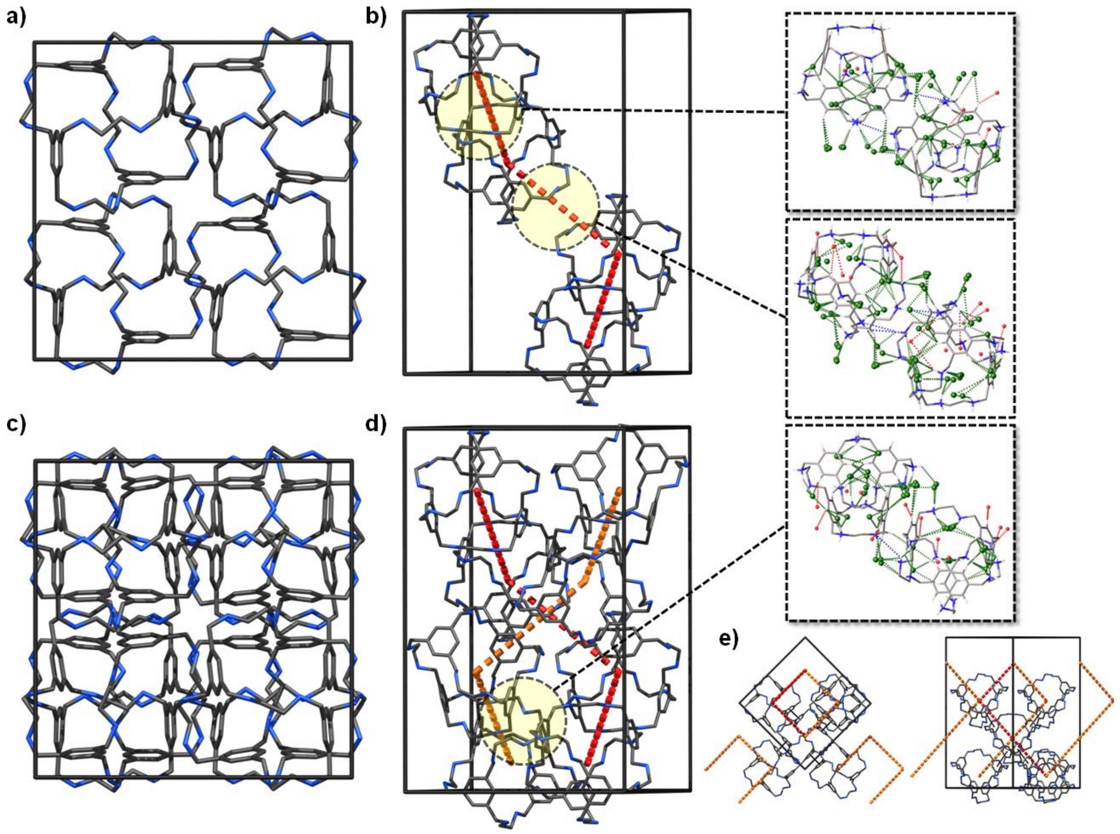

Supplementary Figure 7: Representation of the crystal structure $(H_{12}\mathbf{RCC1})^{12+}\cdot 12Cl^{-}\cdot 4(H_2O)$ (**1**)**:** Crystal packing of crystallographically equivalent $(H_{12}\mathbf{RCC1})^{12+}$ molecules related by fourfold screw axis parallel to *c*, perspective view [001] (a). Helically-arranged, crystallographically equivalent $(H_{12}\mathbf{RCC1})^{12+}$ molecules, related by fourfold screw axis, graphically linked with red dotted line, and are bound through a network of hydrogen bonding interactions (b). Crystal packing of crystallographically distinct $(H_{12}\mathbf{RCC1})^{12+}$ molecules, perspective view [001] (c). Crystallographically distinct $(H_{12}\mathbf{RCC1})^{12+}$ networks, graphically connected with red or orange dotted lines, are intertwined with one another along *c* and linked through a series of H-bonded interactions (d). π-π stacking interactions are evident between crystallographically distinct $(H_{12}\mathbf{RCC1})^{12+}$ networks, depicted with black dashed line, aromatic centroid separation distance 4.2–4.3 Å (e). Unit cell axes shown as thick black line.

It was possible to solve the single crystal structure of $(H_{12}\mathbf{RCC1})^{12+}\cdot 12Cl^{-}$ in the enantiomorphic space groups $P4_122$ and $P4_322$, however the structural model, which comprised one complete **RCC1** molecule, and twelve disordered chloride anions, was highly unstable during refinement.

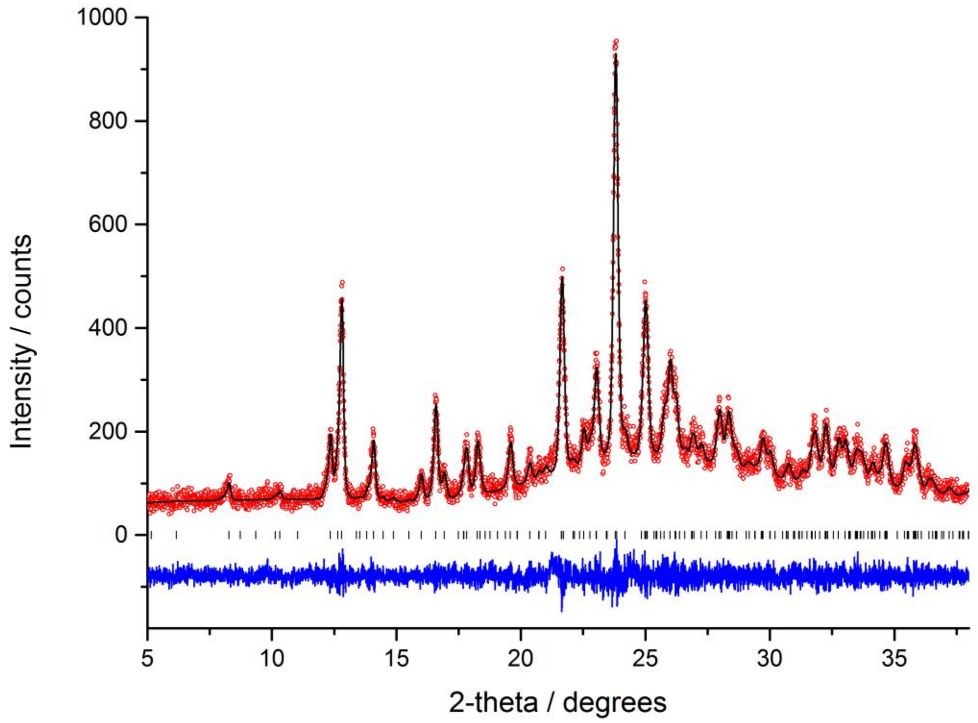

Supplementary Figure 8. Final observed (*red circles*), calculated (*black line*) and difference (*blue*) profiles for Le Bail refinement (R_{wp} = 9.13 %, R_p = 7.04 %, χ^2 = 1.06) of **1** as made (*a* = *b* = 20.265(1) Å, *c* = 32.082(3) Å, *V* = 13175(2) Å^3, $I4_1$). Reflection positions are also marked.

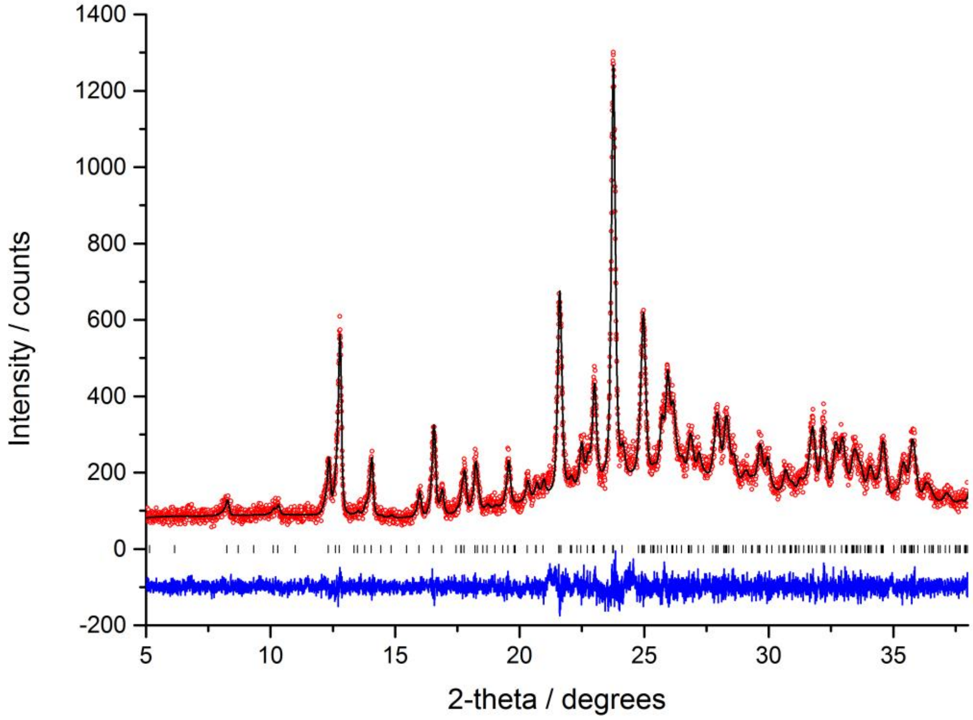

Supplementary Figure 9. Final observed (*red circles*), calculated (*black line*) and difference (*blue*) profiles for Le Bail refinement (R_{wp} = 8.11 %, R_p = 6.24 %, χ^2 = 1.10) of **1** after exposure to 95 % relative humidity (*a* = *b* = 20.3142(8) Å, *c* = 32.185(2) Å, *V* = 13282(1) Å^3, $I4_1$). Reflection positions are also marked.

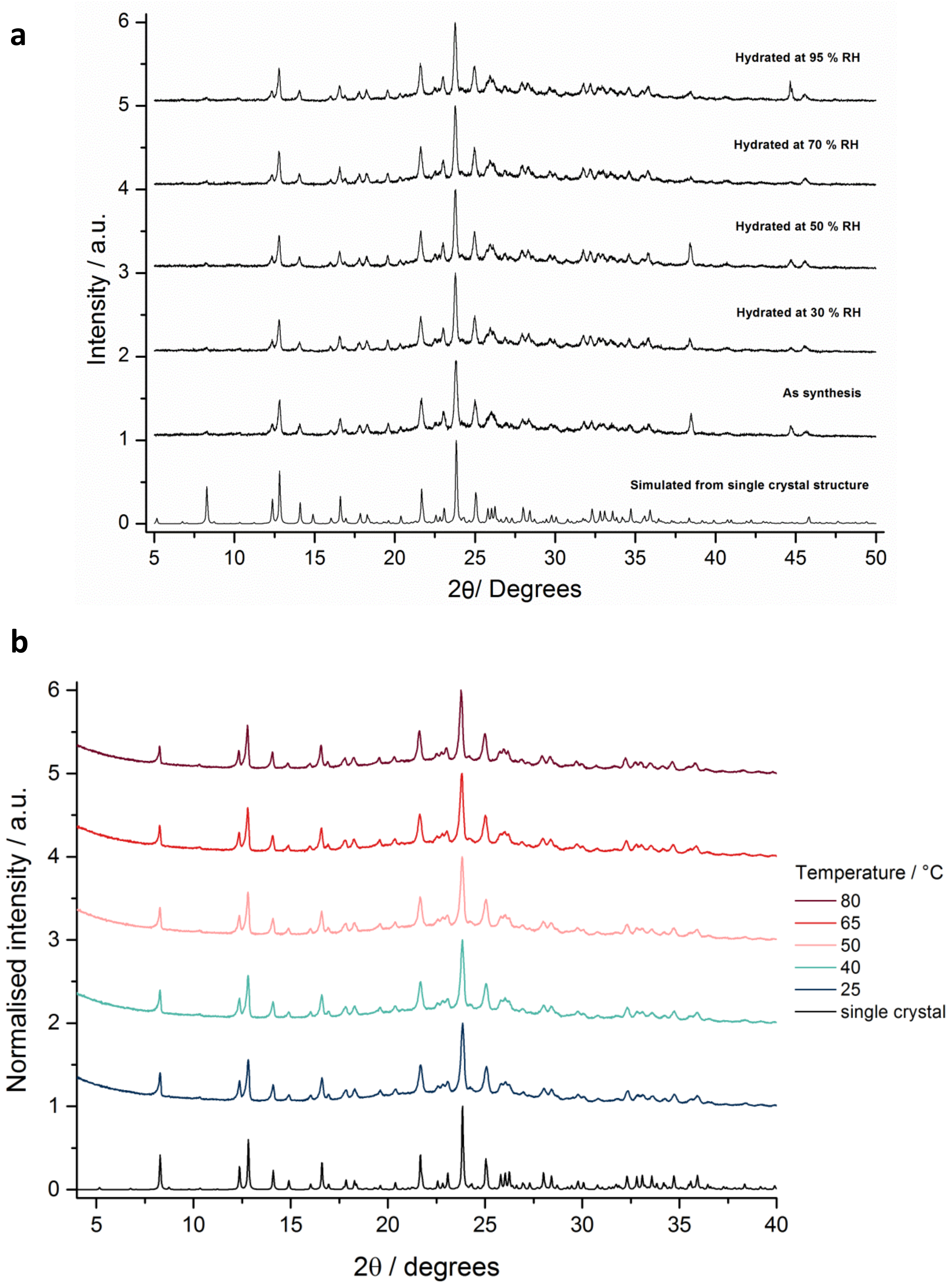

Supplementary Figure 10. Powder X-ray diffraction patterns for samples of **1** subjected to (a) variable humidity conditions at 303 K and (b) variable temperature after being humidified at 95 % RH overnight.

PXRD data were collected for samples exposed to various relative humidity conditions during proton conductivity measurements (Supplementary Fig. 10a - stack plot of patterns). The diffraction patterns

indicate no significant difference in structure of the materials exposed to different humidity. Le Bail refinement confirmed that the bulk synthesised material was consistent with the single crystal structure (Supplementary Fig. 9 - Le Bail fit of **1**) and that the material was unchanged after the measurement (Supplementary Fig. 10a - Le Bail fit of **1**). Lattice parameters for the samples were extracted for all samples (Supplementary Table 1), showing only a small change in cell dimensions with increased humidity. Variable temperature PXRD was also collected for a sample of **1** humidified for approximately 15 hrs under 95 % RH (Supplementary Fig. 10b). The sample was allowed to equilibrate at each temperature for 10 minutes before data collection *in situ*. The diffraction patterns show good agreement with the profile simulated from the single crystal structure is retained up to 80 ℃, with a limited increase in the unit cell size indicated by a small shift of peaks to lower 2θ with increasing temperature.

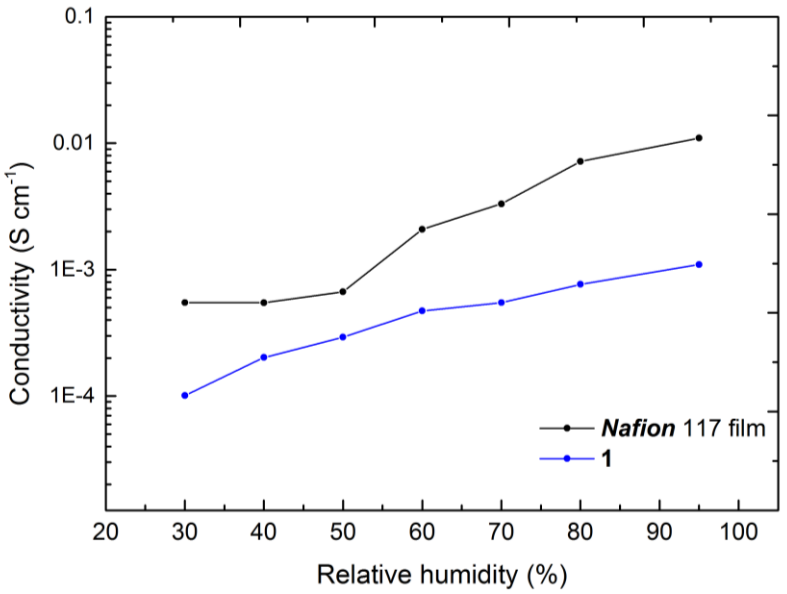

Supplementary Figure 11. Comparison of **1** and commercial *Nafion* film at 303 K with variable relative humidity.

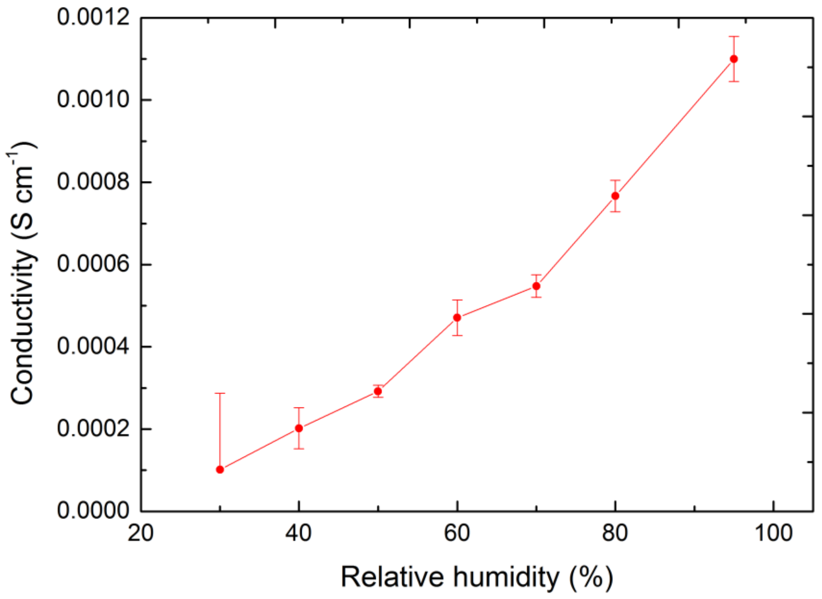

Supplementary Figure 12. Proton conduction of **1** at 303 K subjected to variable relative humidity. The error bars shown represent the value of ±1 standard error of the mean of four consecutive samples that were tested, which are defined as σ/√n, where σ = standard deviation and n = number of samples tested.

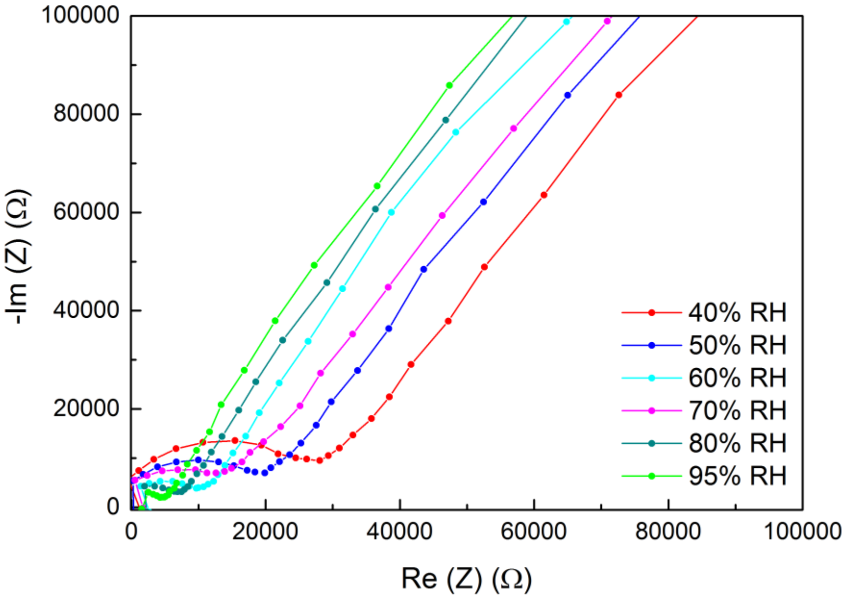

Supplementary Figure 13. Nyquist plots for **1** at 303 K subjected to variable relative humidity.

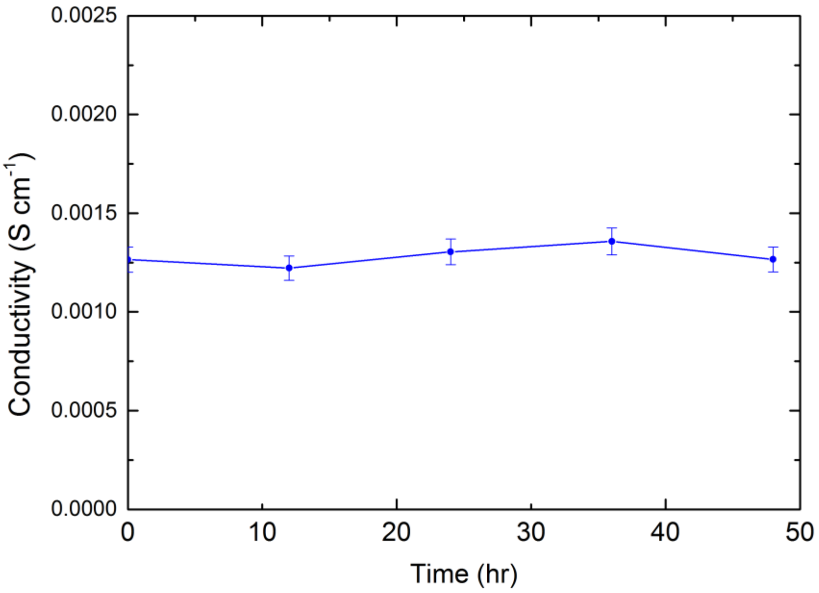

Supplementary Figure 14. Stability of **1** over 48 hours at 303 K and 95 % RH. The error bars shown represent the value of ±1 standard error of the mean of four consecutive samples that were tested, which are defined as $\sigma/\sqrt{n}$, where σ = standard deviation and n = number of samples tested.

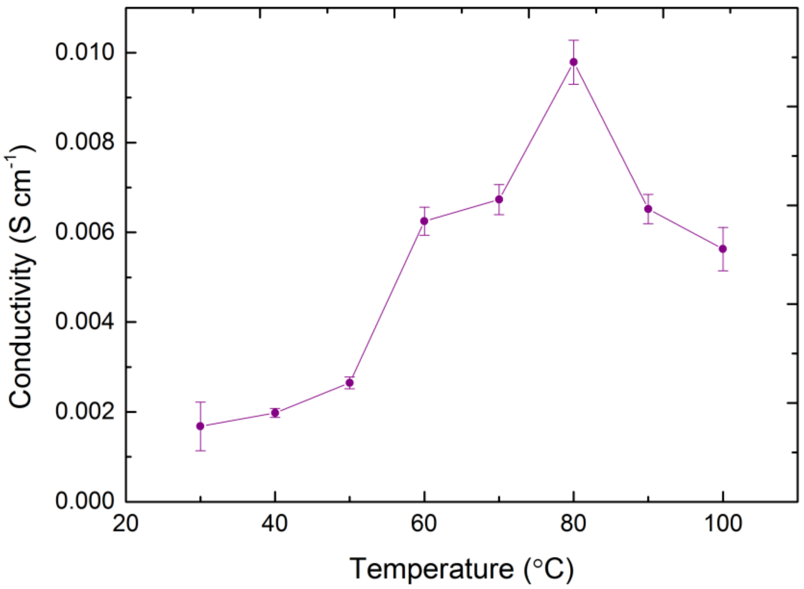

Supplementary Figure 15. Conductivity of **1** subjected to variable temperature under 95 % RH. The error bars shown represent the value of ±1 standard error of the mean of four consecutive samples that were tested, which are defined as $\sigma/\sqrt{n}$, where σ = standard deviation and n = number of samples tested.

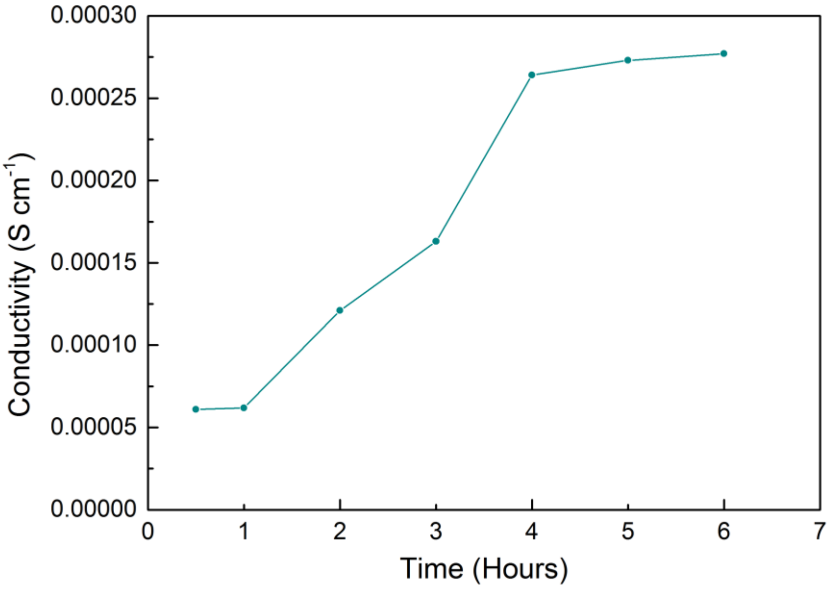

Supplementary Figure 16. Equilibration time data for **1** at 30 % RH and 303 K. Equilibration time is approximately 4 hours.

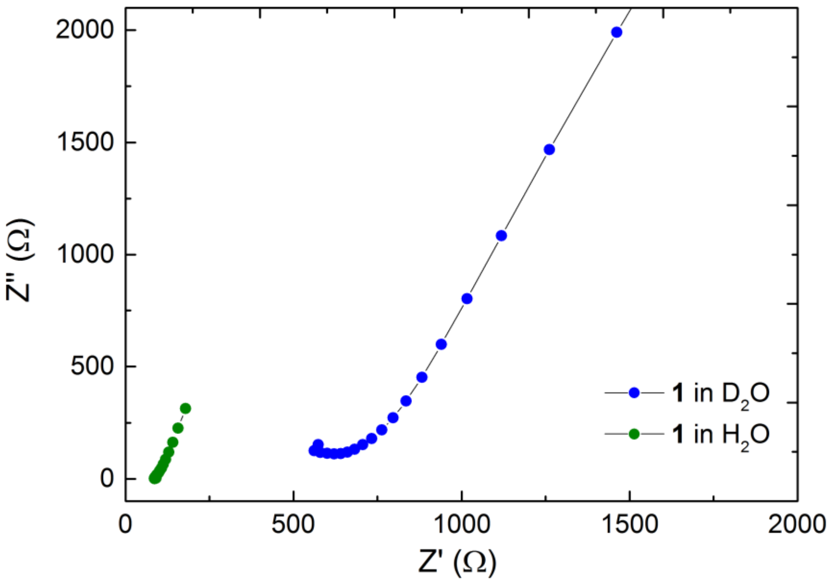

Supplementary Figure 17. Nyquist plots of **1** under H_2O and D_2O atmosphere at 95 % RH and 303 K.

In order to confirm the proton conductivity of **1**, conductivities were measured by humidifying samples in deuterium oxide (D_2O). An isotope effect is observed in the Nyquist plot (Supplementary Fig. 17) provides further evidence that measured conductivity is mainly due to proton carriers, due to the significant increase in resistance observed for the D_2O saturated sample.

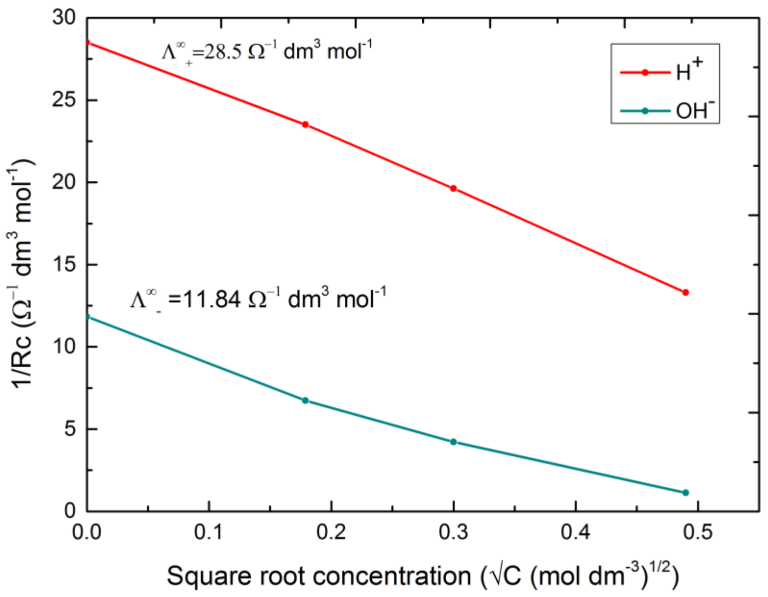

Supplementary Figure 18. Plot of limiting molar conductivities (at infinite dilution) of HCl and CsOH using **1**. The proton transference number, t^{+} was calculated to be 0.71 (71 %).

The conductivity of **1** chloride salt determined by electrochemical impedance spectroscopy is the total conductivity of the system. The cage structure contains chloride ions, which may be too large to be transported through the cage windows. However, under humid conditions it could in principle be possible for the chloride ions to be transported through grain boundaries. Impedance, while reliable, is not a selective technique because polarisation of the system results in the formation of oppositely charged electrodes, to which positive and negative ions can be attracted. To provide an estimation of the extent of proton conductivity, a method for determining the proton transference number, t^{+}, was followed. Pak and Xu[1] used a similar procedure to determine the proton transference of *Nafion* and XUS films.

The transference number of **1** was determined to be 0.71 (71%) indicating that a large proportion of the conductivity involves protons as charge carriers: this is comparable to *Nafion* which has a proton transference of 0.72 as determined by Pak and Xu[1]. Chloride ions show much lower mobility and thus play at most a minor role in the conductivity of **1**.

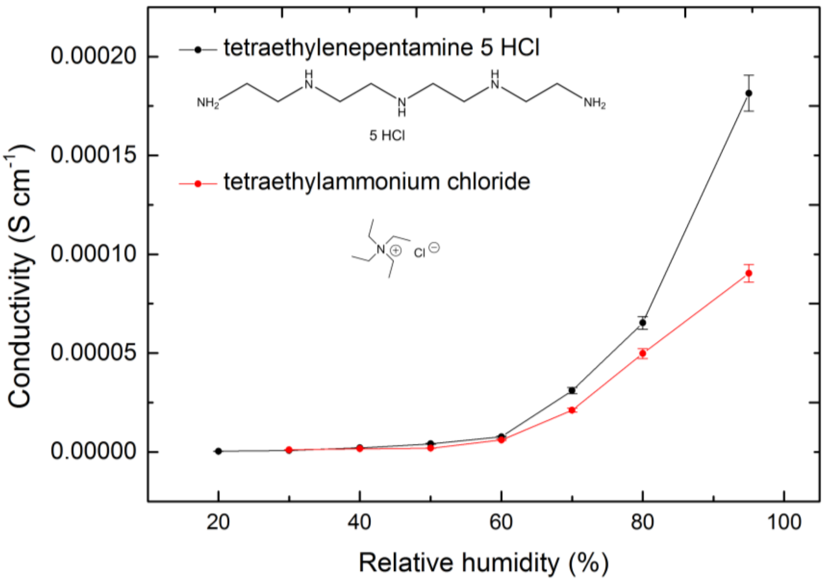

Supplementary Figure 19. Proton conductivity of structurally related amine salts as control materials. The error bars shown represent the value of ±1 standard error of the mean of four consecutive samples that were tested which are defined as σ/√n where σ = standard deviation and n = number of samples tested.

This figure shows the mean conductivity of tetraethylenepentamine pentahydrochloride and tetraethylammonium chloride at 293 K as a function of relative humidity. Tetraethylenepentamine pentahydrochloride shows a maximum conductivity of 1.82×10^{-4} S cm^{-1} at 95 % RH, owing to the five protonated amine groups. The tetraethylammonium chloride showed a conductivity of 9.04×10^{-5} S cm^{-1} at 95 % RH (it has no protonated amine groups). Samples were equilibrated for four hours between taking measurements. In both cases, the proton conductivities are much lower than observed for cage salt **1**.

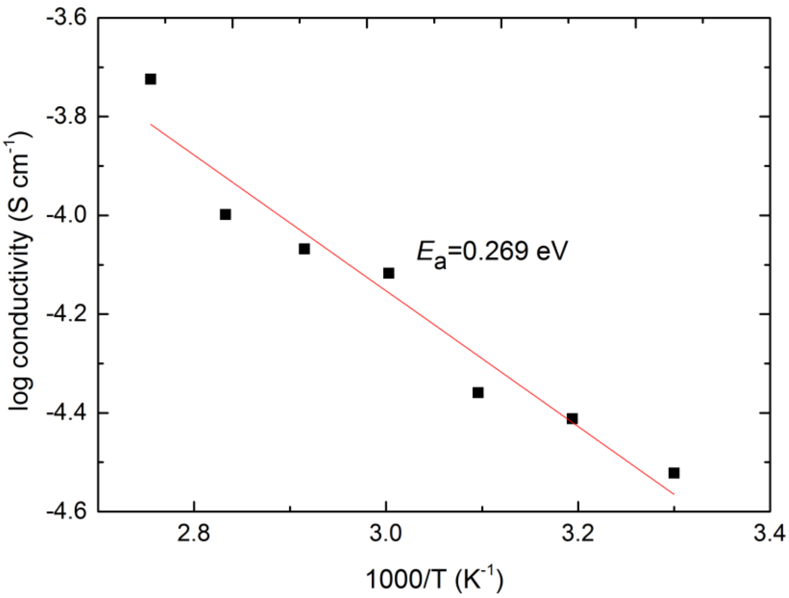

Supplementary Figure 20. Arrhenius plot for the tetraethylenepentamine pentahydrochloride conductivity data showing an activation energy of 0.269 eV. Temperature data were collected at 95 % relative humidity over the temperature range 293–363 K.

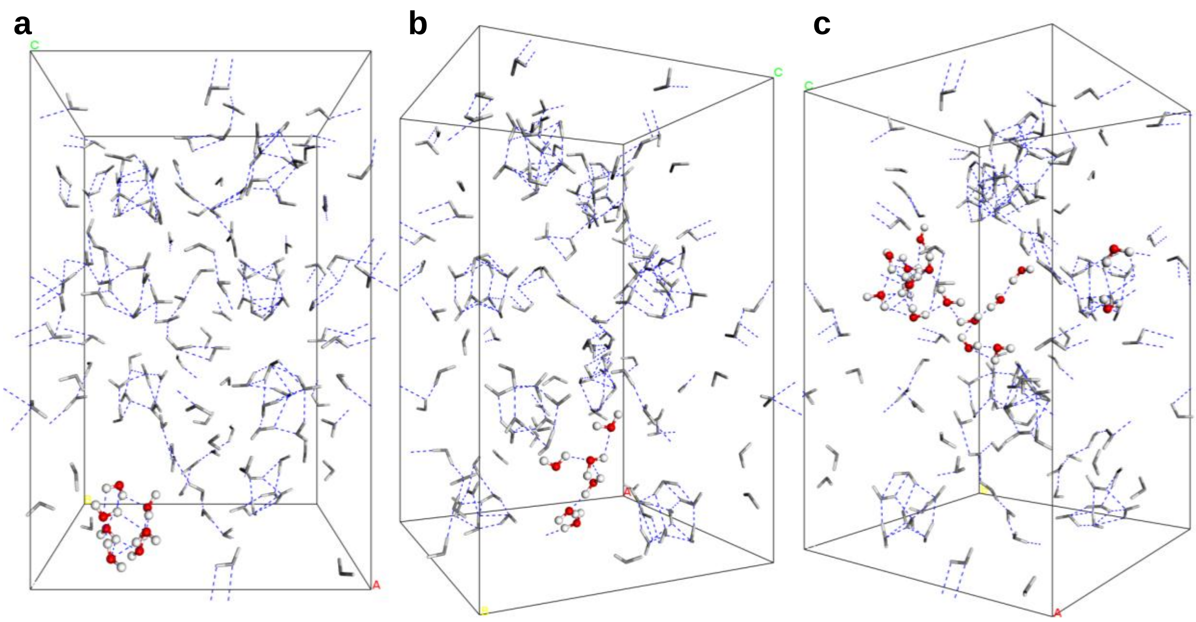

Supplementary Figure 21. Molecular configurations of water adsorbed in **1** at 95 % relative humidity at 298.16 K, taken from a continuous fractional component Monte Carlo (CFCMC) simulation. The cage molecules and chloride ions were omitted for clarity; *i.e.*, only the adsorbed water molecules are shown, with the hydrogen bonds between them indicated by dashed blue lines. The simulation snapshot is viewed from different angles in **a**, **b**, and **c**, where different water environments are highlighted by showing the water molecules in the ball-and-stick representation: (**a**) a water cluster formed inside the cavity of a cage molecule; (**b**) a hydrogen-bonded chain of water molecules in the extrinsic void space between the cage molecules; and (**c**) a water cluster, inside the cage cavity, connected to the hydrogen-bond network outside the cage (*i.e.*, in the extrinsic void space).

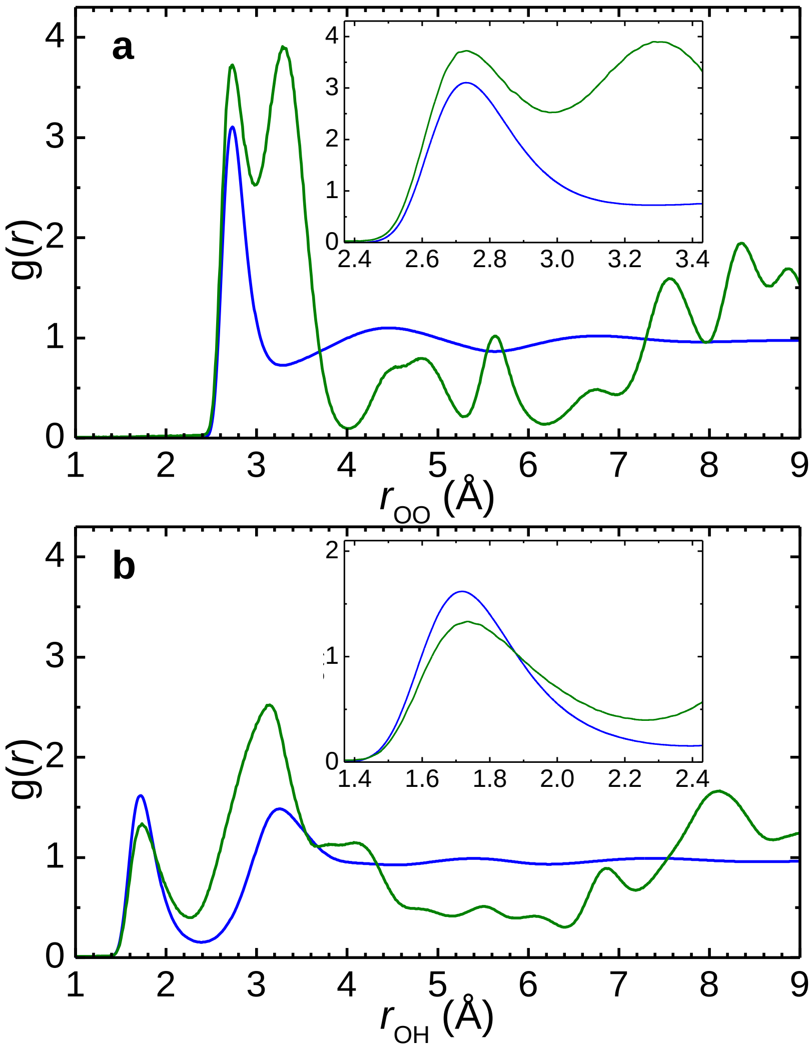

Supplementary Figure 22. The water adsorbed in the extrinsic pores of **1** is less confined than the water clusters in the cage cavities (see Main Text, Figure 3). Radial distribution functions (RDFs) for (**a**) oxygen–oxygen and (**b**) oxygen–hydrogen pairs between water molecules, obtained from a CFCMC simulation of water in **1** at 95 % relative humidity (green) or from an *NPT* MD simulation of water in bulk at 1 bar (blue), both at 298.16 K; the insets are a magnification of the first RDF peaks in the respective figures. In the CFCMC simulation of **1**, all of the cage cavities were blocked to water, *i.e.*, the RDFs shown here solely characterize the spatial distributions of water in the extrinsic void space in **1**.

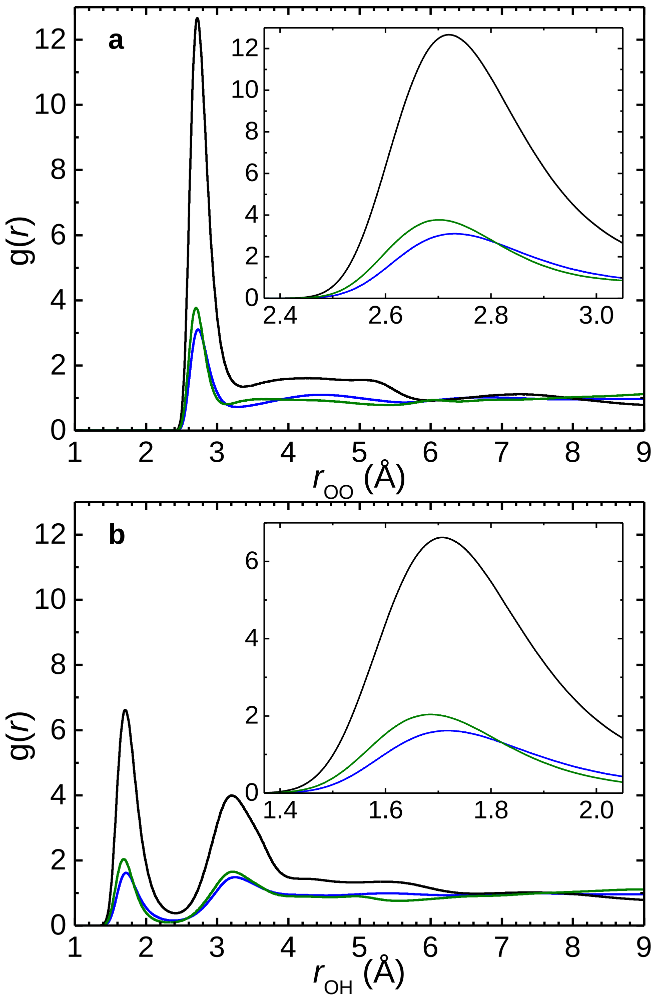

Supplementary Figure 23. Radial distribution functions of (**a**) oxygen–oxygen and (**b**) oxygen–hydrogen pairs between water molecules in **1** at 95 % relative humidity (green), **CC3** at 95 % relative humidity (black), and bulk water at 1 bar (blue), all obtained from *NPT* MD simulations at 298.16 K; the insets are a magnification of the first RDF peaks in the respective figures.

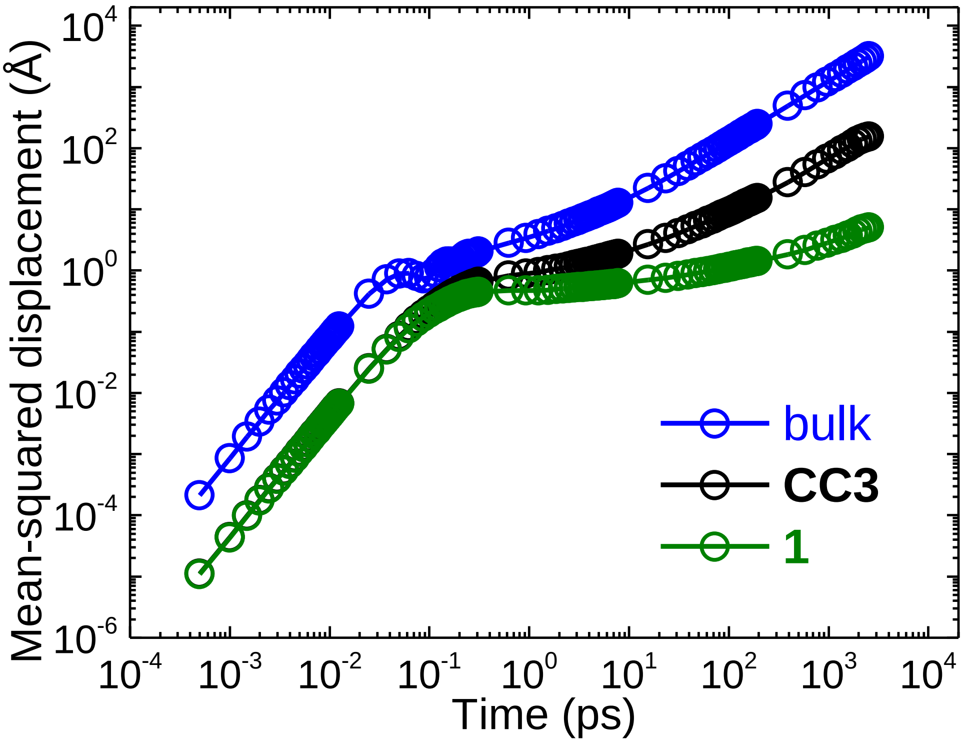

Supplementary Figure 24. Mean-squared displacement (MSD) of water at 298.16 K in bulk (1 bar), **CC3** (95 % relative humidity), and **1** [**1**; 95 % relative humidity], all obtained from *NPT* MD simulations. Higher displacement indicates greater average diffusion speed. The different slope of the MSD curves, in the (near) linear region between 10^1 and 10^4 ps, suggest that the translational diffusivity of water decreases in the order of bulk > **CC3** > **1**.

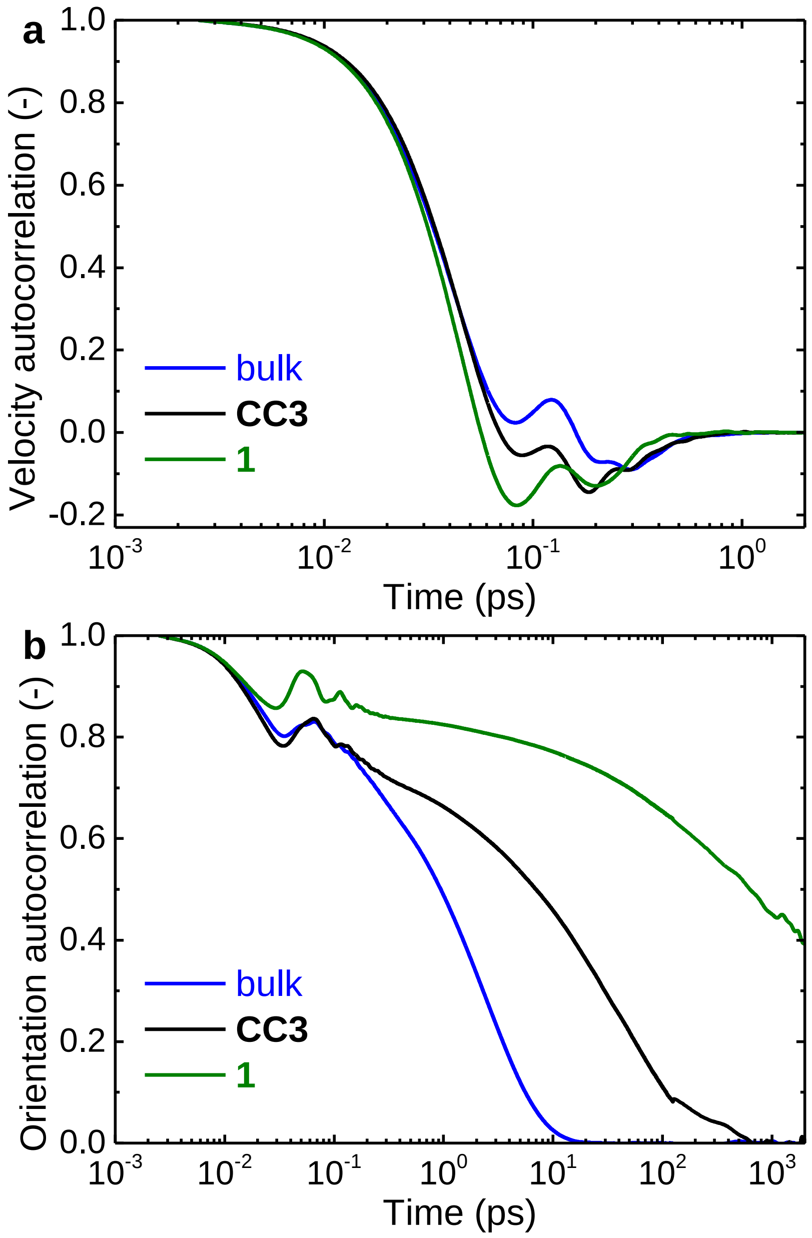

Supplementary Figure 25. (**a**) Velocity auto-correlation functions (VACFs) and (**b**) molecular orientation auto-correlation functions (MOACFs) for water at 298.16 K in bulk (1 bar), **CC3** (95 % relative humidity), and **1**; 95 % relative humidity, all obtained from *NPT* MD simulations.

Molecular dynamics simulations reveal that the translational diffusivity of water is significantly slowed down in **1** (Supplementary Fig. 25), suggesting that **1** might have a better water retention ability, which further helps to attain high water-mediated protonic conductivity.

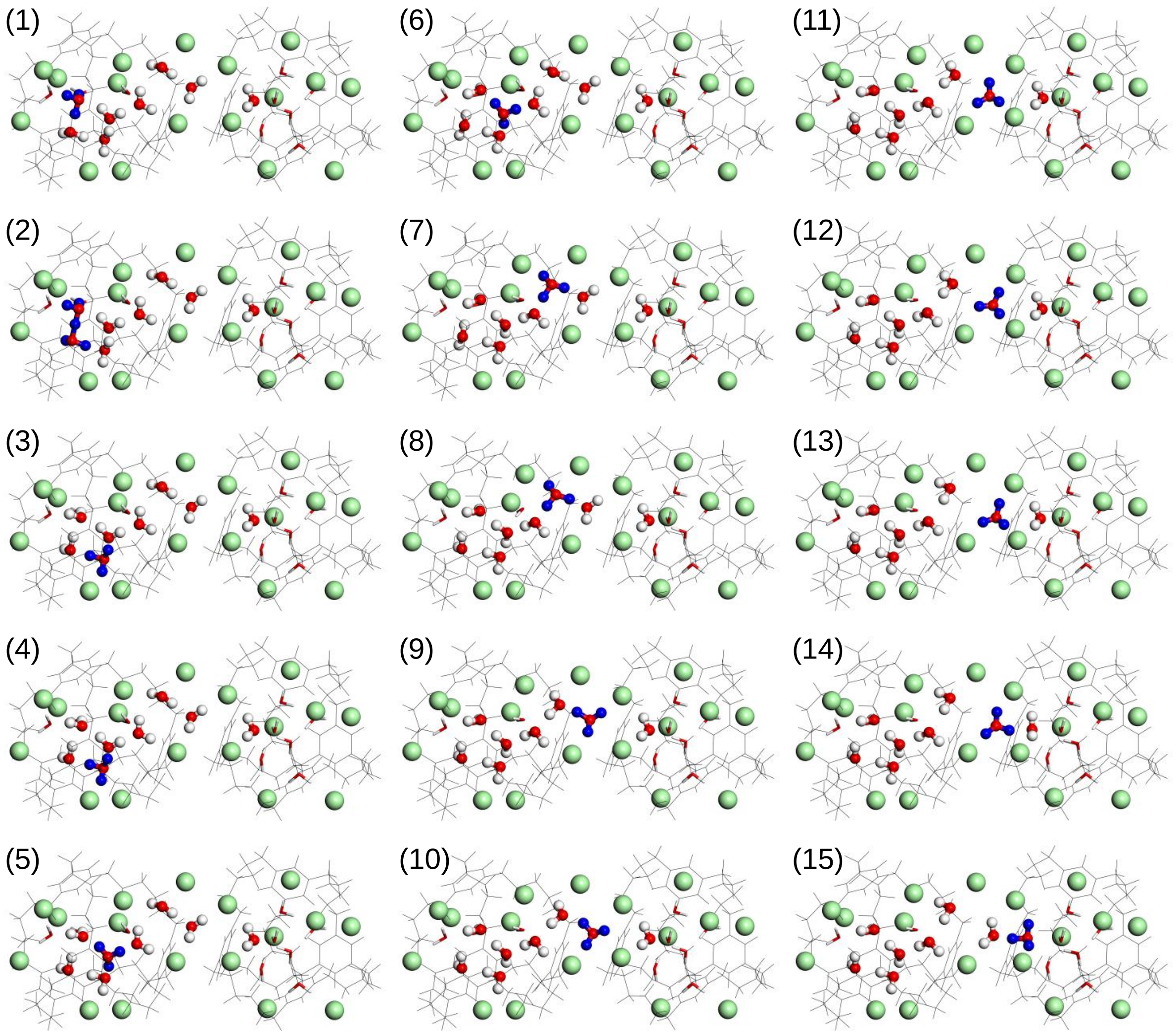

Supplementary Figure 26. Individual molecular configurations along the minimum-energy pathway for migration of an excess proton between two neighbouring cages in **1**, where the cages are in grey, chloride ions in green, oxygen in red, and hydrogen in white or blue (protons involved in the migration are coloured blue). For clarity, surrounding cages, chloride ions, and water molecules are not shown.

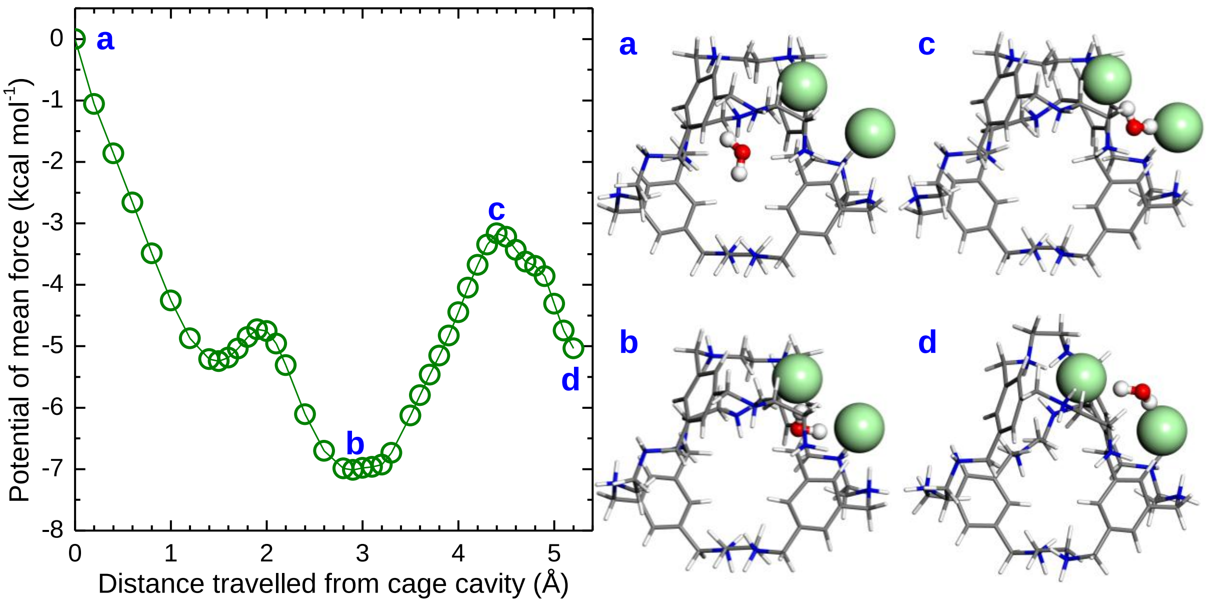

Supplementary Figure 27. Potential of mean force (PMF) for a water molecule travelling from the inside of a cage cavity to the extrinsic void space between the cages in **1**, plotted as a function of the reaction coordinate which is the distance between the centre of mass of the water molecule and the centre of mass of the cage. The water molecule started at the centre of a cage cavity (a), and then moved out of the cage and into the extrinsic void space through a cage window (b–d). The PMF at the starting position was arbitrarily set to zero. All the simulations were done with one water molecule in a single unit cell of the cage salt structure comprising 8 H_{12}**RCC1**$^{12+}$ cage molecules and 96 Cl^{-} ions, while only the most relevant entities, as taken from simulation snapshots, are shown here for clarity.

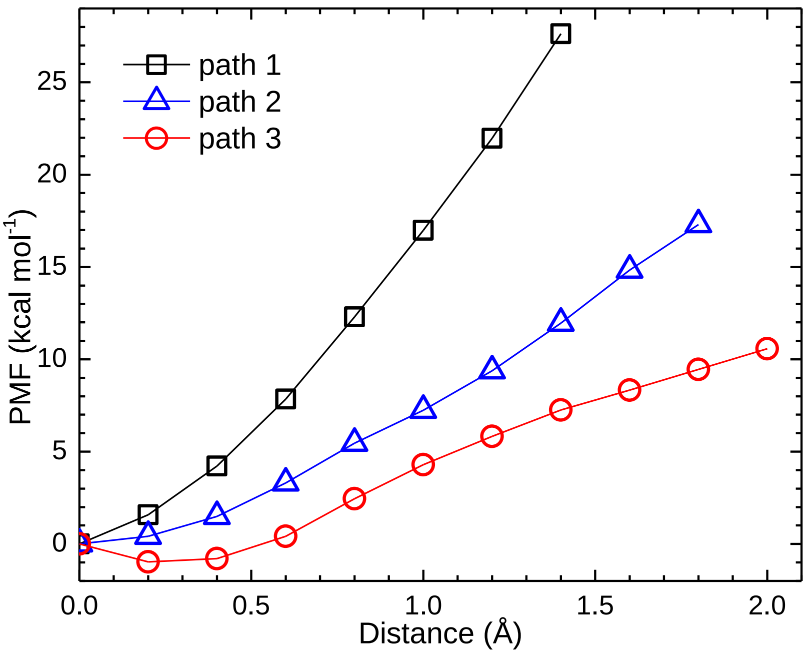

Supplementary Figure 28. Potential of mean force (PMF) profiles for three different paths of a Cl^- ion diffusing in **1**: (i) the Cl^- ion travelling in the extrinsic void space (path 1); (ii) travelling in the extrinsic void space with one neighbouring Cl^- ion removed, *a priori*, to create a vacant site on its pathway (path 2), and; (iii) travelling from the outside to the inside of a nearby cage molecule (path 3). The PMFs at the starting positions were arbitrarily set to zero. In the cases of paths 1 and 3, the simulations were done with a single unit cell of the cage salt structure comprising 8 H_{12}**RCC1**$^{12+}$ cage molecules and 96 Cl^- ions. The simulation box for path 2 consisted of 95 Cl^- ions, 7 H_{12}**RCC1**$^{12+}$ cage molecules, and 1 H_{11}**RCC1**$^{11+}$ cage molecule, maintaining an overall electrical neutrality of the whole system. The H_{11}**RCC1**$^{11+}$ cage molecule was created by deleting one hydrogen atom, bonded to an imine nitrogen atom, from the H_{12}**RCC1**$^{12+}$ cage molecule furthest away from the Cl^- ion of interest.

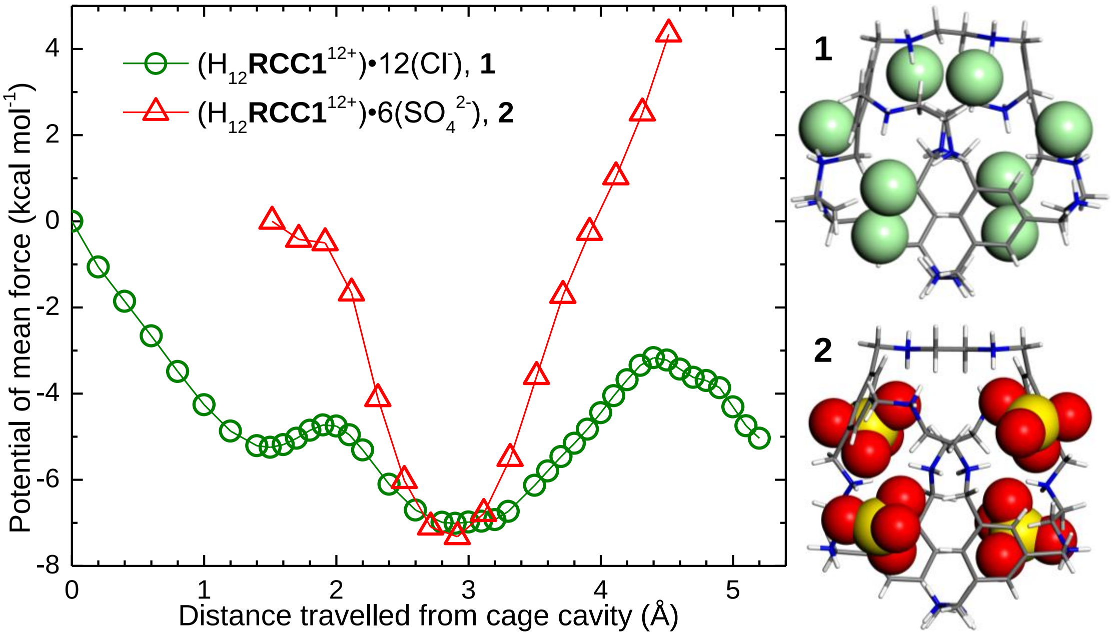

Supplementary Figure 29. Potential of mean force (PMF) profiles of a water molecule travelling from the inside of a cage cavity to the extrinsic void space between the cages, as calculated for **1** and for **2**; also shown are two H_{12}**RCC1**$^{12+}$ cages with their nearest ions in the two cage salt structures. Both PMF profiles were plotted as a function of the reaction coordinate which is the distance between the centre of mass of the water molecule and the centre of mass of the cage. The water molecule started at the centre of a cage cavity in **1** or at a position (inside the cage) 1.5 Å away from the cage centre in **2**, and then moved out of the cage and into the extrinsic void space through a cage window. In both cases, the PMF at the starting position was arbitrarily set to zero. All the simulations were done with one water molecule in a single unit cell of the cage salt structure comprising 8 H_{12}**RCC1**$^{12+}$ cage molecules and 96 Cl^{-} ions (in **1**) or 48 SO_4^{2-} ions (in **2**).

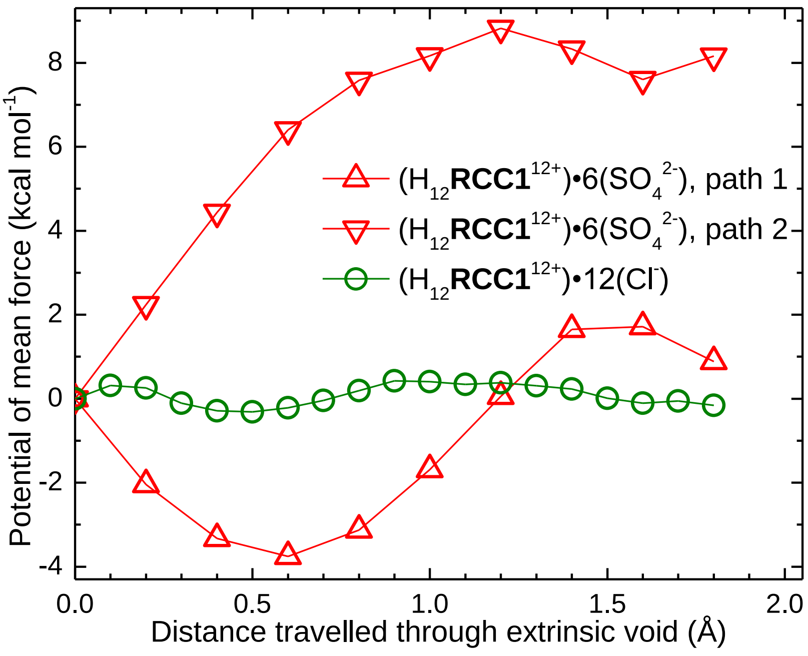

Supplementary Figure 30. Potential of mean force (PMF) profiles for a water molecule travelling in the extrinsic void space in **1** and **2**; for the latter, a water molecule was placed at two different positions in the extrinsic void space, followed by two separate sets of MD simulations to obtain the PMFs for the two different diffusion paths. The reaction coordinate was the distance between the centre of mass of the water molecule and the centre of a cage window (defined by the centre of mass of all non-hydrogen atoms on the window), and the PMF at the starting position was arbitrarily set to zero. All the simulations were done with one water molecule in a single unit cell of the cage salt structure comprising 8 H_{12}**RCC1**$^{12+}$ cage molecules and 96 Cl^{-} ions or 48 SO_4^{2-} ions.

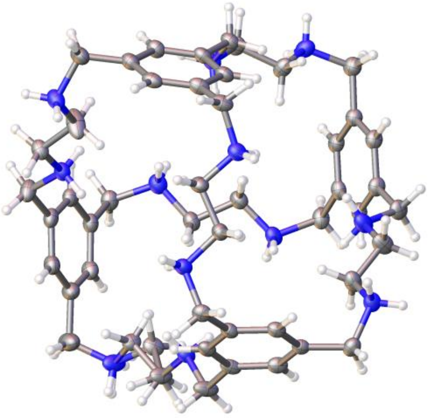

Supplementary Figure 31. Displacement elliposoid plot from the single crystal structure $(H_{12}\mathbf{RCC1})^{12+}\cdot 12(SO_4)^{2-})\cdot 27.25(H_2O)$ recorded at 100 K. Sulphate anions and H_2O molecules omitted for clarity. Ellipsoids displayed at 50 % probability level.

The asymmetric unit for $(H_{12}\mathbf{RCC1})^{12+}\cdot 6(SO_4)^{2-}\cdot 27.25(H_2O)$ comprises 1 complete $(H_{12}\mathbf{RCC1})^{12+}$ molecule; 7 $(SO_4)^{2-}$ anions; and 27.5 H_2O molecules refined over 44 positions. Two sulphate anions were refined with rigid bond restraints, as was part of a disordered ethyl cage vertex (RIGU in SHELX). H_2O molecules were disordered in the crystal structure, and it was not possible to accurately resolve H-atom positions (checkCIF PLAT306_ALERT_2_B alert). H-atoms for these H_2O molecules were added to the unit cell atom count (checkCIF PLAT043_ALERT_1_B alert). In the crystal structure hydrogen bonding interactions between H_2O molecules and the $(SO_4)^{2-}$ anions resulted in short intermolecular D-A distances (checkCIF PLAT430_ALERT_2_B alert). For a displacement ellipsoid plot of the asymmetric unit, see Supplementary Fig. 31.

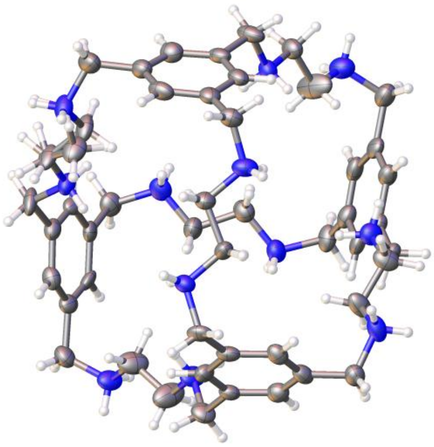

Supplementary Figure 32. Displacement elliposoid plot from the single crystal structure (H_{12}**RCC1**)$^{12+}$·6(SO4)$^{2-}$·27.25(H_2O) (**2**) recorded at 293 K. Sulphate anions and H_2O molecules omitted for clarity. Ellipsoids displayed at 50 % probability level.

A second single crystal of **2** was mounted onto a MiTeGen gripper directly from the crystallisation solvent and transferred into MiTeGen RT tube filled with H_2SO_4 (aq.). The crystal was kept immersed during the data collection that was recorded at 293 K. Crystal data for (H_{12}**RCC1**)$^{12+}$·6(SO_4)$^{2-}$ ·27.25(H_2O) (**2**) recorded at 293 K; CCDC entry: 1452673. Formula $C_{48}H_{138.50}N_{12}O_{51.25}S_6$; M = 1896.56 g·mol^{-1}; orthorhombic space group *F*dd2, colourless crystal; a = 32.848(6) Å, b = 34.298(6) Å, c = 31.413(5) Å; V = 35391(10) Å^3; ρ = 1.424 g·cm^{-3}; μ = 0.259 mm^{-3}; F (000) = 16264; crystal size = 0.44 x 0.33 x 0.32 mm; T = 293(2) K; 114198 reflections measured (1.717 < θ 25.681°), 15472 unique (R_{int} = 0.0653), 12588 (I > 2 (I)); R_1 = 0.0751 for observed and R_1 = 0.0915 for all reflections; wR_2 = 0.2012 for all reflections; max/min residual electron density = 0.667 and -0.298 e·Å^{-3}; data/restraints/parameters = 15472/108/1311; GOF = 1.096. Flack parameter 0.00(3). The asymmetric unit for **RCC1**·6(SO_4)·27.25(H_2O) comprises a complete **RCC1** molecule; 7 (SO_4)$^{2-}$ anions; and, in total, 27.5 H_2O molecules refined over 45 positions. Part of a disordered ethyl cage vertex, and three (SO_4)$^{2-}$ anions, were refined with rigid bond restraints (DELU and RIGU in SHELX). At 293 K disorder in the crystal structure resulted in low C-C bond precision (checkCIF PLAT340_ALERT_3_B alert). H_2O molecules were disordered in the crystal structure, and it was not possible to accurately resolve H-atom positions (checkCIF PLAT306_ALERT_2_B alert). H-atoms for these H_2O molecules were added to the unit cell atom count (checkCIF PLAT043_ALERT_1_B alert). In the crystal structure hydrogen bonding interactions between H_2O molecules and the (SO_4)$^{2-}$ anions resulted in short intermolecular D-A distances (checkCIF PLAT430_ALERT_2_B alert). For a displacement ellipsoid plot, see Supplementary Fig. 32.

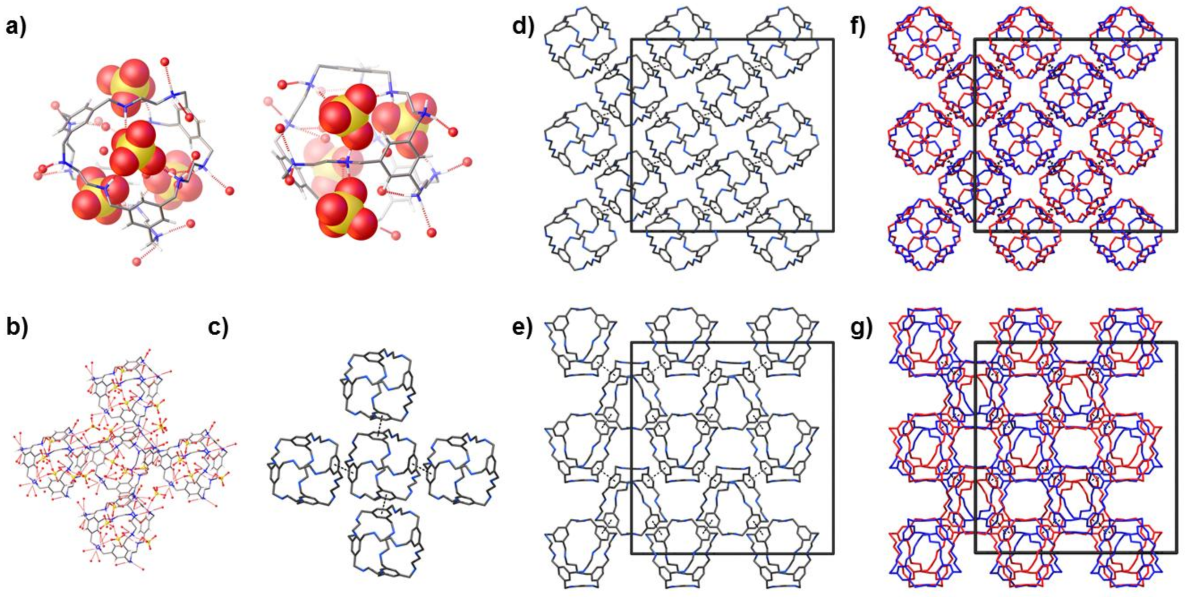

Supplementary Figure 33. (a) Hydrogen bonded $(SO_4)^{2-}$ anions are located in the cage windows of $(H_{12}\mathbf{RCC1})^{12+}$. (b) Extended window-to-window packing of $(H_{12}\mathbf{RCC1})^{12+}$ molecules, hydrogen bonding interactions between $R_2NH_2^+$ and aryl C-H to $(SO_4)^{2-}$ and H_2O molecules shown as dashed lines. (c) offset π-π stacking interactions evident between $(H_{12}\mathbf{RCC1})^{12+}$ molecules in the crystal lattice, aromatic centroid separation distance 3.5–3.6 Å. Extended π-π stacking network of $(H_{12}\mathbf{RCC1})^{12+}$ molecules; perspective view [010] (d) and [001] (e). Two π-π stacked networks of $(H_{12}\mathbf{RCC1})^{12+}$ molecules (highlighted red and blue) and interpenetrated with one another; perspective view [010] (f) and [001] (g). 1-D channels in the crystal structure are filled with $(SO_4)^{2-}$ anions and lattice solvent.

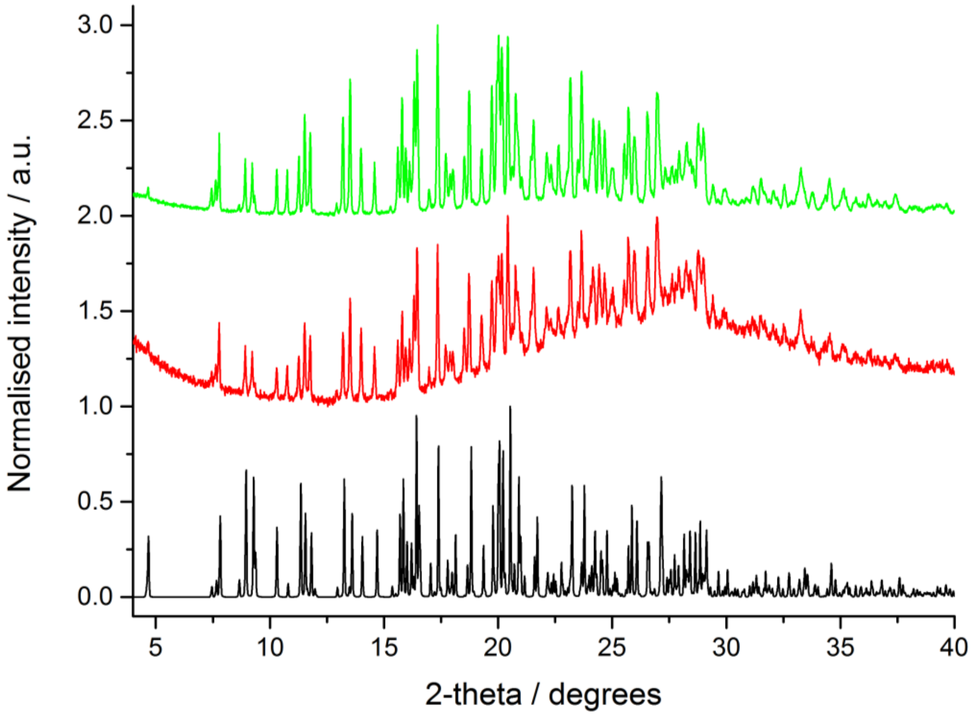

Supplementary Figure 34. Comparison of PXRD patterns for hydrated bulk **2** surface-dried (*green*) and slurried in aqueous crystallisation medium (*red*) with simulated pattern from single crystal (100 K, *black*).

A sample of **2** was ground in its aqueous mother liquor to produce a slurry which was loaded into a 0.7 mm diameter borosilicate glass capillary. The capillary was sealed and diffraction data were collected at ambient temperature to obtain a PXRD profile of the fully hydrated material (Supplementary Fig. 34). Le Bail fitting of this pattern (Supplementary Fig. 35) confirmed its purity and that the single crystal structure was representative of the bulk.

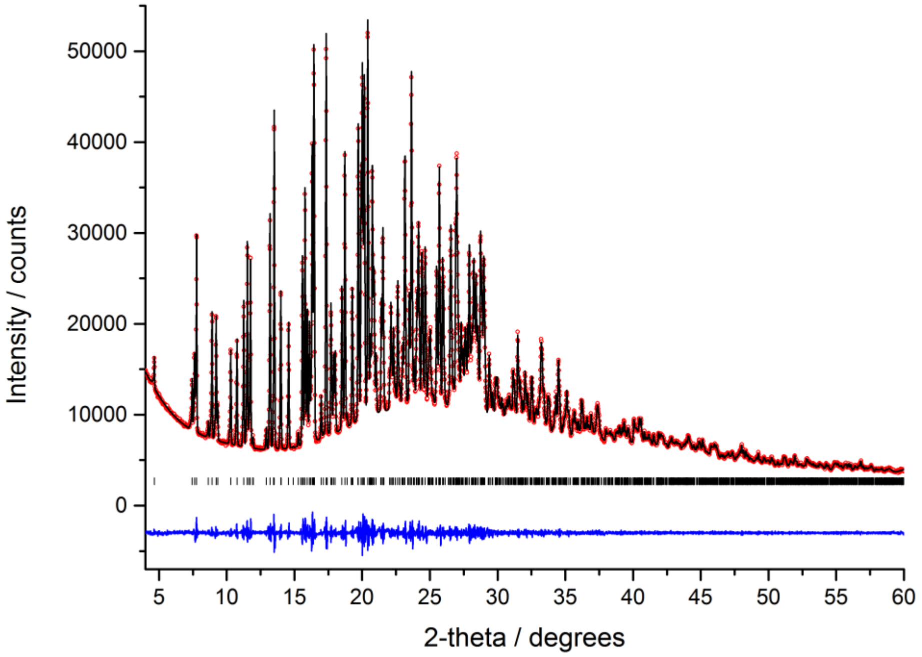

Supplementary Figure 35. Final observed (*red circles*), calculated (*black line*) and difference (*blue*) profiles for Le Bail refinement (R_{wp} = 2.20 %, R_p = 1.57 %, χ^2 = 2.22) of **2** slurried in aqueous mother liquor (a = 34.3337(2), b = 31.3791(2), c = 31.4205(2) Å, V = 35469.4(4) Å^3, *Fdd*2). Reflection positions are also marked.

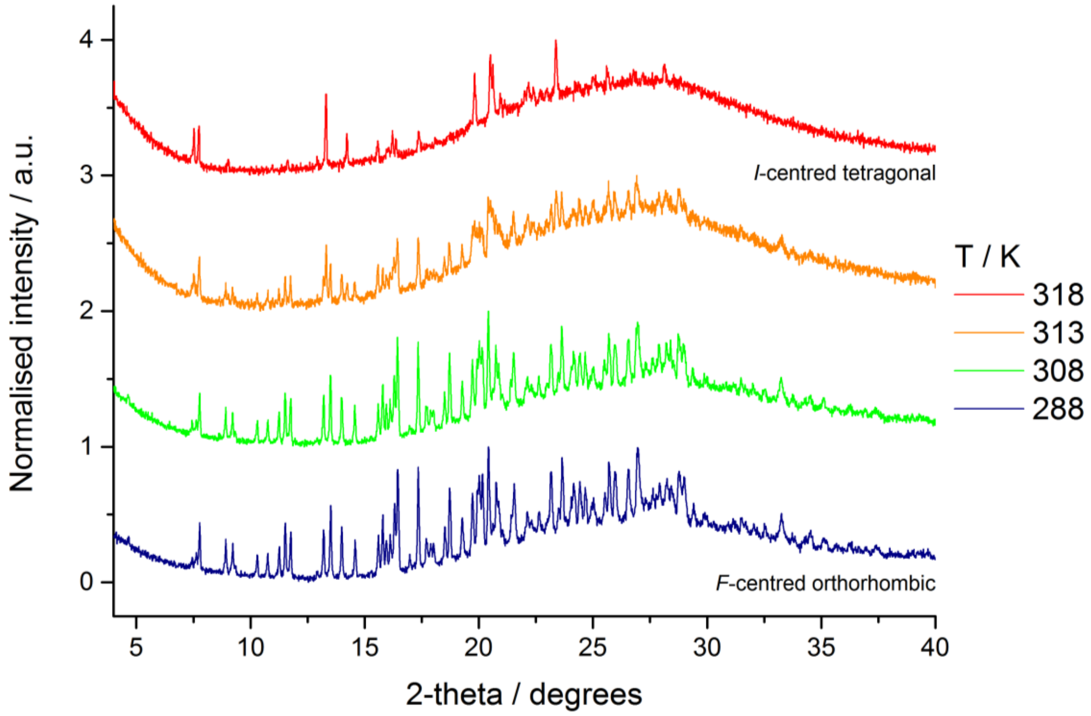

Supplementary Figure 36. VT-PXRD for **2** slurred in aqueous mother liquor shows a phase transition occurs above 313 K which is complete by 318 K. Dissolution in water becomes significant at this temperature, resulting in loss of diffraction.

The capillary was opened and variable temperature PXRD (VT-PXRD) data were subsequently collected between 288–318 K (Supplementary Fig. 36) to monitor the effect of temperature on **2**. A phase transition was observed above 313 K, which was complete by 318 K. The solubility of **2** was significant at this temperature, resulting in dissolution of the crystalline sample, and loss of Bragg diffraction intensity.

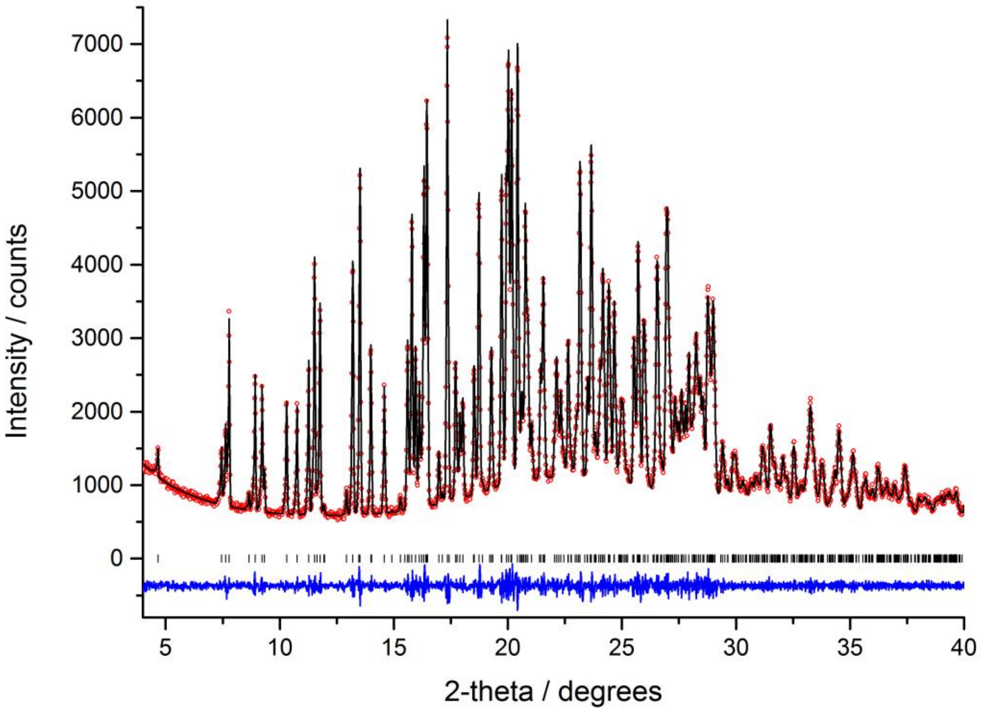

Supplementary Figure 37. Final observed (*red circles*), calculated (*black line*) and difference (*blue*) profiles for Le Bail refinement (R_{wp} = 3.48 %, R_p = 2.65 %, χ^2 = 1.35) of surface-dried **2** at 288 K (*a* = 34.3223(4), *b* = 32.8716(4), *c* = 31.3894(4) Å, *V* = 35414.3(7) Å^3, *Fdd*2). Reflection positions are also marked.

A second hydrated sample of **2** was prepared by decanting off the aqueous crystallisation medium and drying the surface of the crystals by briefly touching with a filter paper. The crystals were ground and loaded into a 1 mm diameter borosilicate glass capillary. The similarity of the PXRD patterns for the surface-dried and slurried samples at 288 K (Supplementary Fig. 34) and Le Bail fitting (Supplementary Fig. 37) indicated that the surface-dried sample remains representative of the fully hydrated **2** structure. VT-PXRD data were collected in the range 288–333 K (Supplementary Fig. 38). The onset of the phase transition was again observed at 313 K, with the original *F*-centred orthorhombic structure persisting as the dominant phase (Supplementary Fig. 39). At 318 K, a single *I*-centred tetragonal phase is present (Supplementary Fig. 40), consistent with the structure obtained on heating a single crystal to 293 K (Supplementary Fig. 41).

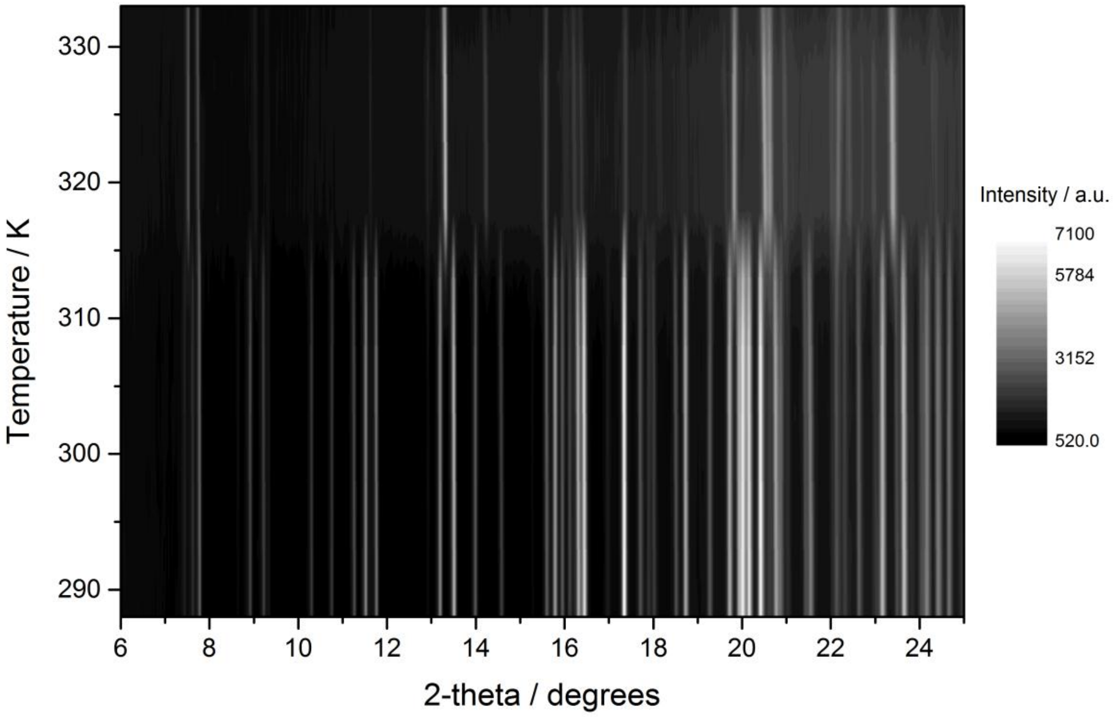

Supplementary Figure 38. VT-PXRD for surface-dried **2** sample shows a phase transition occurs above 313 K and is complete by 318 K.

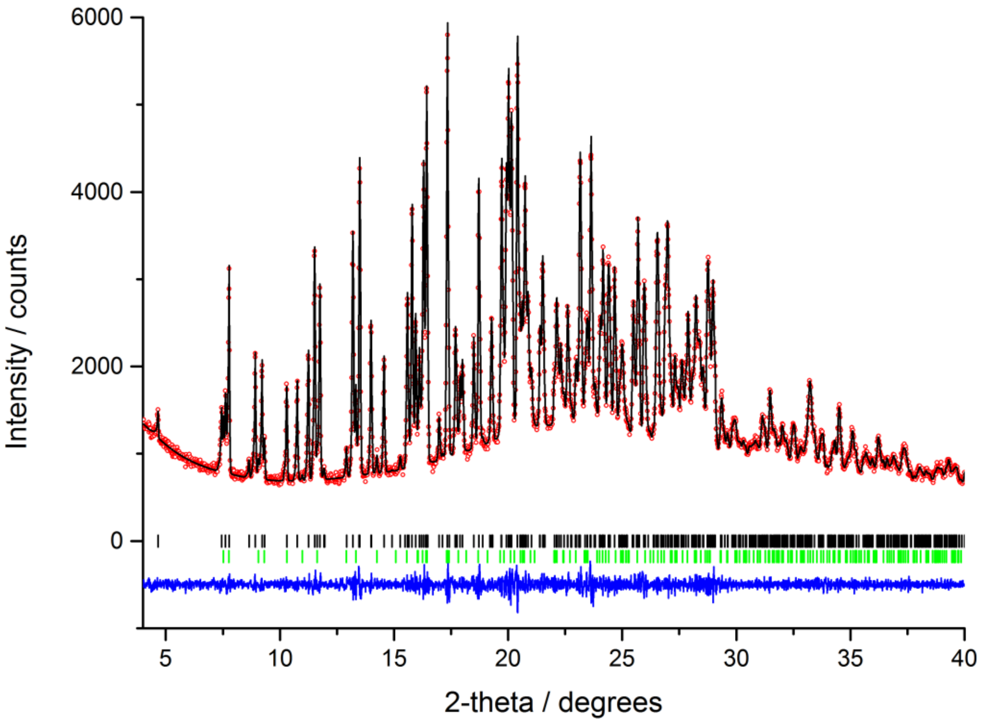

Supplementary Figure 39. Final observed (*red circles*), calculated (*black line*) and difference (*blue*) profiles for Le Bail refinement (R_{wp} = 3.48 %, R_p = 2.65 %, χ^2 = 1.35) of surface-dried **2** at 313 K. Two phases are present which can be fitted with the *F*-centred orthorhombic structure (*a* = 34.3223(4), *b* = 32.8716(4), *c* = 31.3894(4) Å, *V* = 35414.3(7) Å^3, *Fdd*2) as the dominant phase and an *I*-centred tetragonal (*a* = *b* = 22.7693(8), *c* = 34.295(2) Å, *V* = 17780(2) Å^3, $I4_1/acd$) phase growing in. Reflection positions for the two phases are marked.

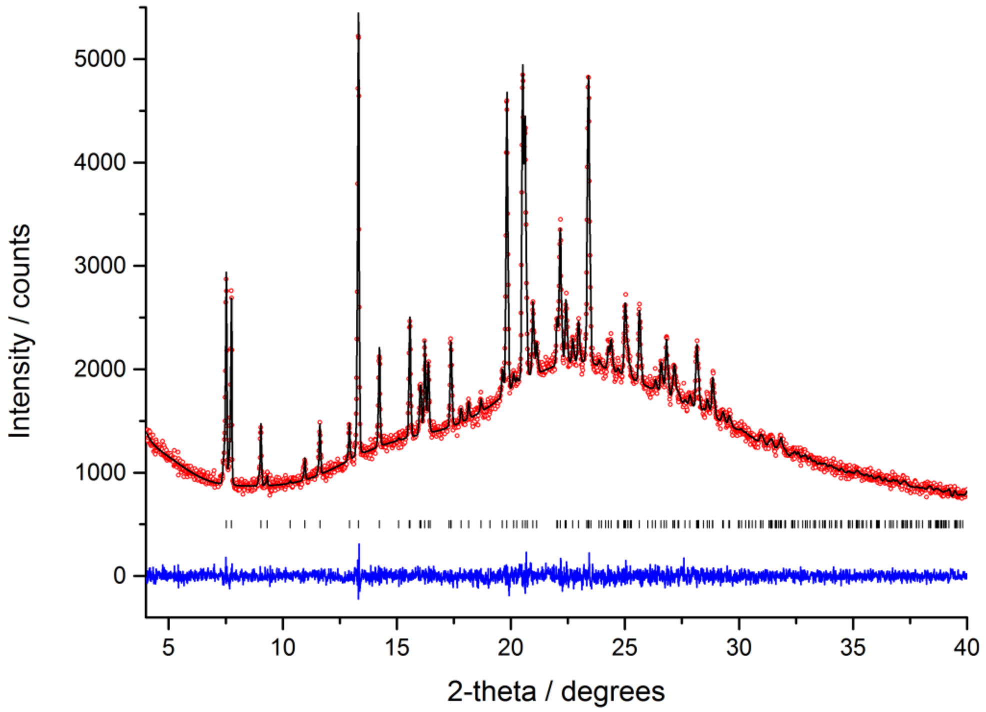

Supplementary Figure 40. Final observed (*red circles*), calculated (*black line*) and difference (*blue*) profiles for Le Bail refinement (R_{wp} = 2.82 %, R_p = 2.22 %, χ^2 = 1.07) of surface-dried **2** at 318 K (a = b = 22.7938(5), c = 34.255(1) Å, V = 17798(1) Å^3, $I4_1/acd$). Reflection positions are also marked.

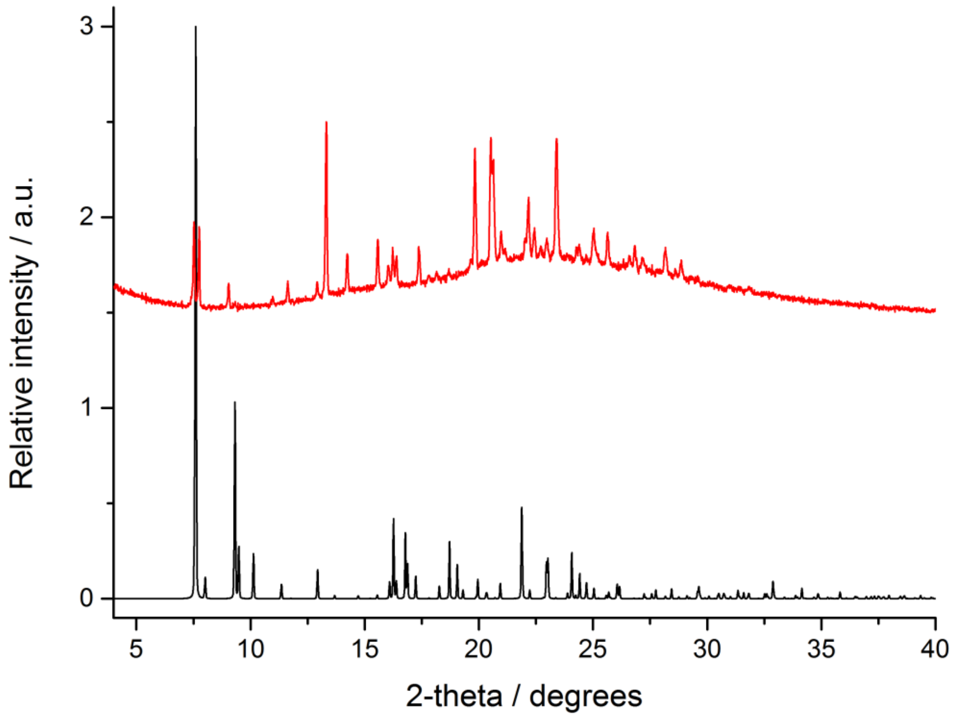

Supplementary Figure 41. Comparison of experimental PXRD pattern for surface-dried sample of **2** at 318 K and profile simulated for the $I4_1/acd$ single crystal phase at 293 K.

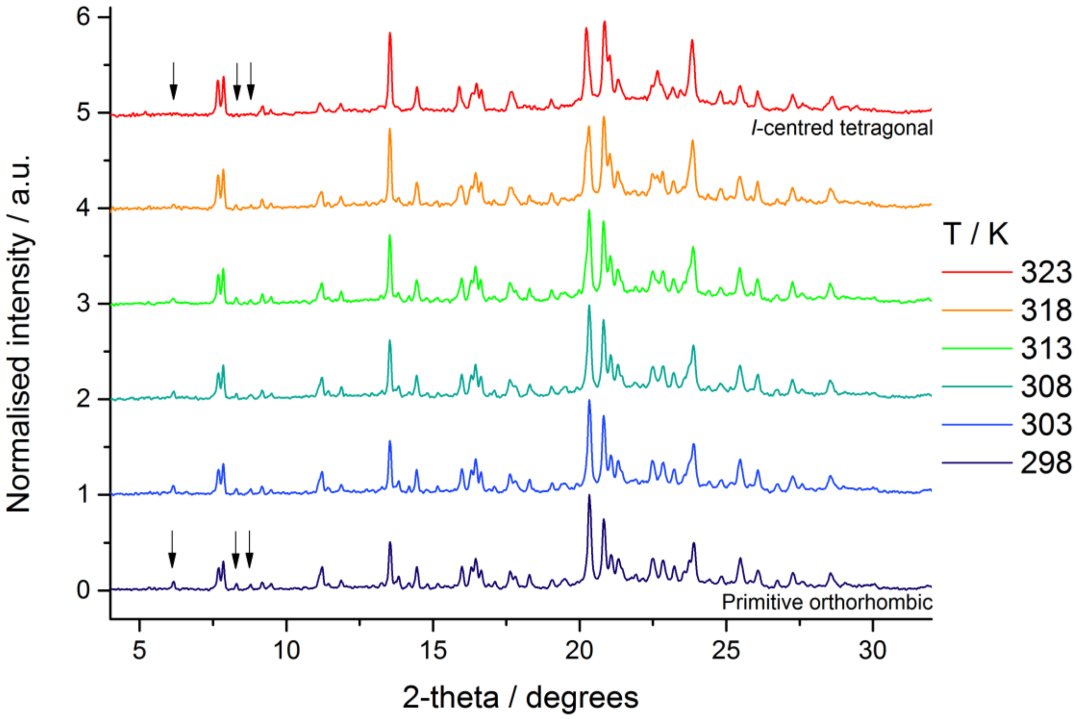

Supplementary Figure 42. VT-PXRD data for air-dried **2** showing a phase transition from a previously unknown primitive phase to the *I*-centred tetragonal structure above 318 K. Arrows indicate most prominent loss of diffraction peaks upon phase transition.

A sample of **2** was prepared by isolating crystals from the mother liquor and allowing them to dry in air on a watch glass overnight. This procedure was analogous to the preparation of material for pellet formation. The crystals were then ground and packed into a 0.7 mm diameter capillary. VT-PXRD data were collected between 298 and 323 K (Supplementary Fig. 42). The 298 K data shows that air-dried **2** adopts a structure not yet characterised by single crystal X-ray diffraction. The PXRD profile was indexed by a single phase; however the space group could not be unambiguously assigned, with a number of possible combinations of unit cell with primitive tetragonal and orthorhombic space groups. Le Bail fitting failed to distinguish between several plausible candidates, so a smaller unit cell was chosen and the space group was tentatively assigned as *Pbca* (a = 22.467(1), b = 33.164(1), c = 22.503(1) Å, V = 16767(1) Å^3, Supplementary Fig. 43) on the basis of a simple subgroup relation (*via Ibca*) with the $I4_1/acd$ structure, and the analogy with the observed $Fdd2 \rightarrow I4_1/acd$ transition. This phase also transforms to the *I*-centred tetragonal structure at 323 K (Supplementary Table 2, Supplementary Fig. 44) above the temperature at which variable humidity conductivity measurements are performed (303 K).

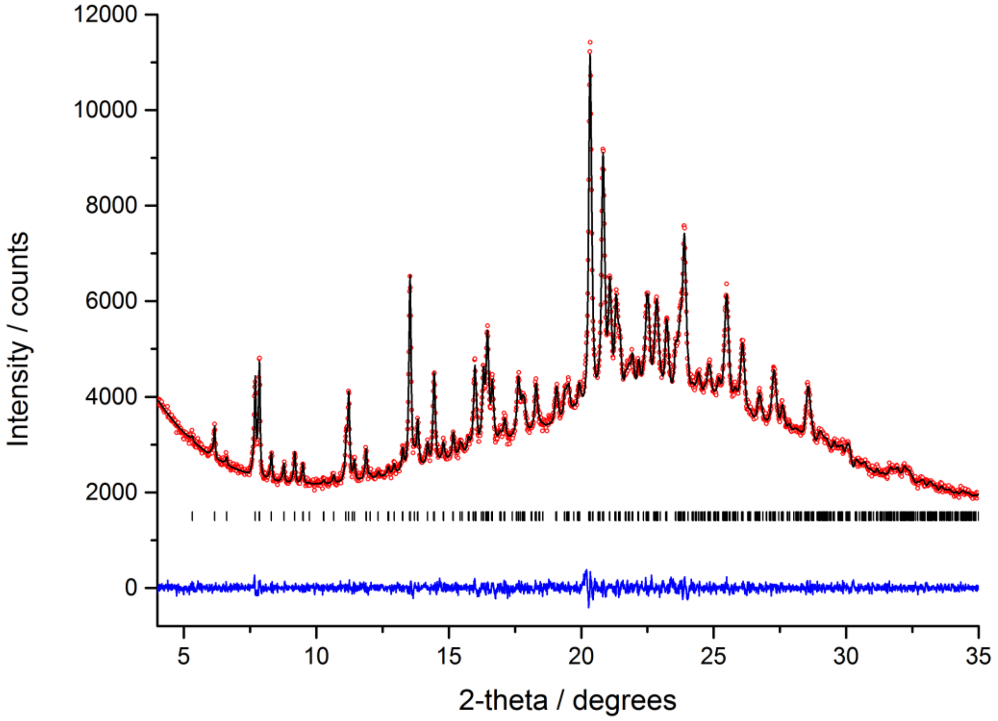

Supplementary Figure 43. Final observed (*red circles*), calculated (*black line*) and difference (*blue*) profiles for Le Bail refinement (R_{wp} = 1.93 %, R_p = 1.50 %, χ^2 = 1.12) of air-dried **2** at 298 K as primitive orthorhombic phase (*a* = 22.467(1), *b* = 33.164(1), *c* = 22.503(1) Å, *V* = 16767(1) Å^3, *Pbca*). Reflection positions are also marked.

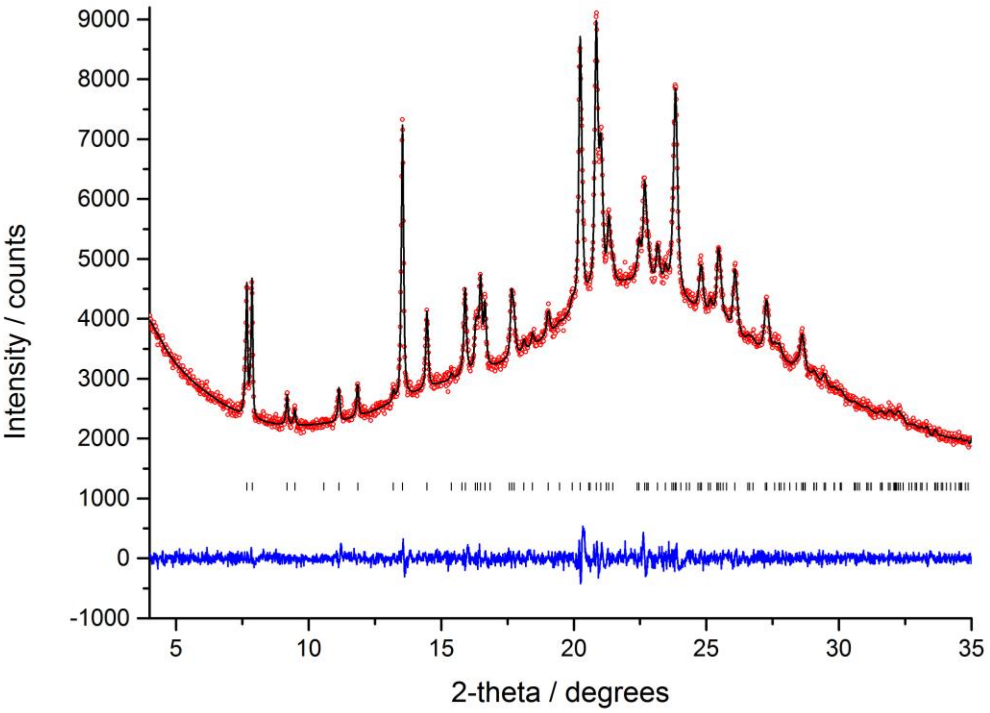

Supplementary Figure 44. Final observed (*red circles*), calculated (*black line*) and difference (*blue*) profiles for Le Bail refinement (R_{wp} = 2.82 %, R_p = 2.22 %, χ^2 = 1.07) of air-dried **2** at 323 K ($a = b$ = 22.4483(8), c = 33.454(2) Å, V = 16859(2) Å^3, $I4_1/acd$), indicating the bulk material is consistent with the single crystal $I4_1/acd$ structure at this temperature. Reflection positions are also marked.

PXRD were collected for a sample of **2** recovered from a pellet after proton conductivity measurement. Comparison of this pattern with the air-dried material (Supplementary Fig. 45) and Le Bail fitting (Supplementary Fig. 46) shows that **2** the original structure is retained after the pellet formation and conductivity measurements.

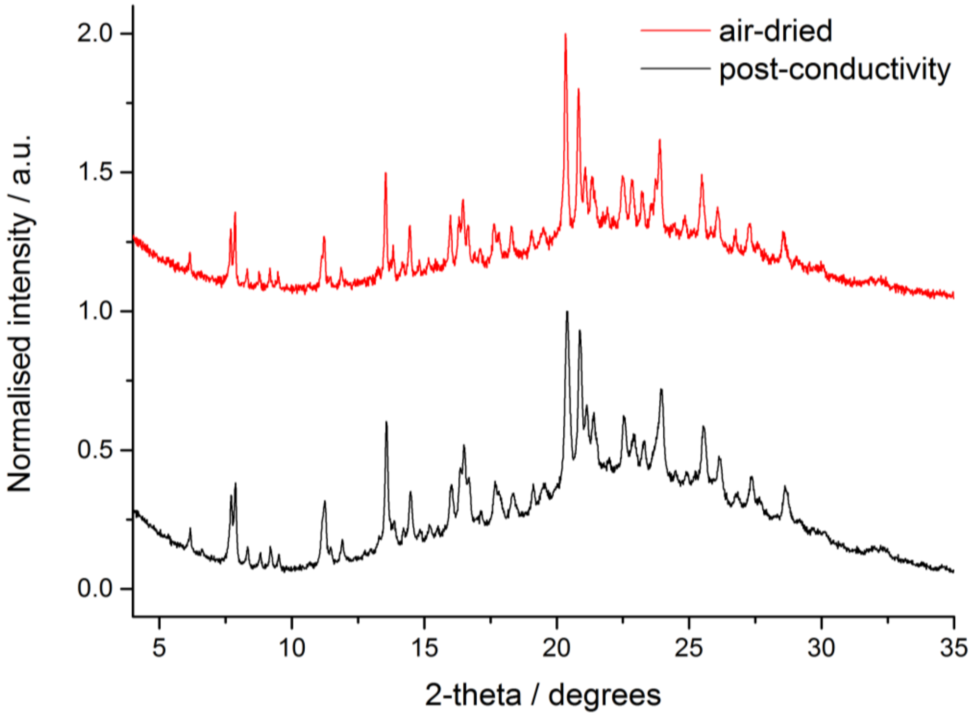

Supplementary Figure 45. PXRD patterns of air-dried **2**, air-dried as for pellet formation (*red*), and recovered from a pellet after conductivity measurement (*black*) show the structure is unchanged by pressing and conductivity testing.

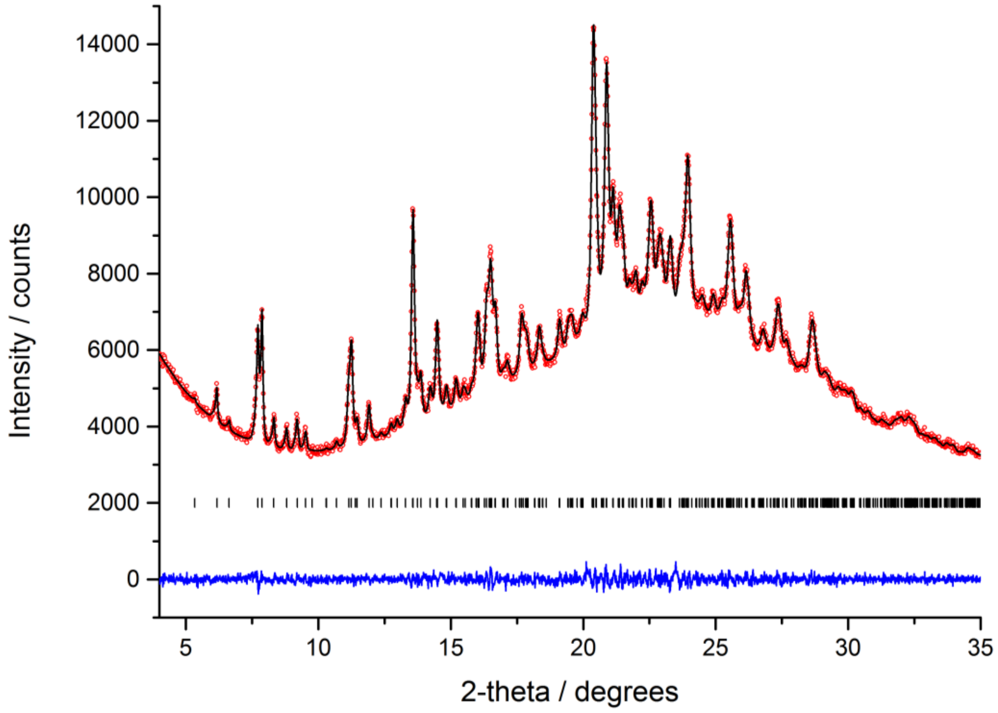

Supplementary Figure 46 Final observed (*red circles*), calculated (*black line*) and difference (*blue*) profiles for Le Bail refinement (R_{wp} = 1.55 %, R_p = 1.23 %, χ^2 = 1.16) of **2** at ambient temperature recovered from a pellet post-conductivity measurement (*a* = 22.420(2), *b* = 33.067(2), *c* = 22.439(2) Å, *V* = 16635(2) Å^3, *Pbca*). Reflection positions are also marked.

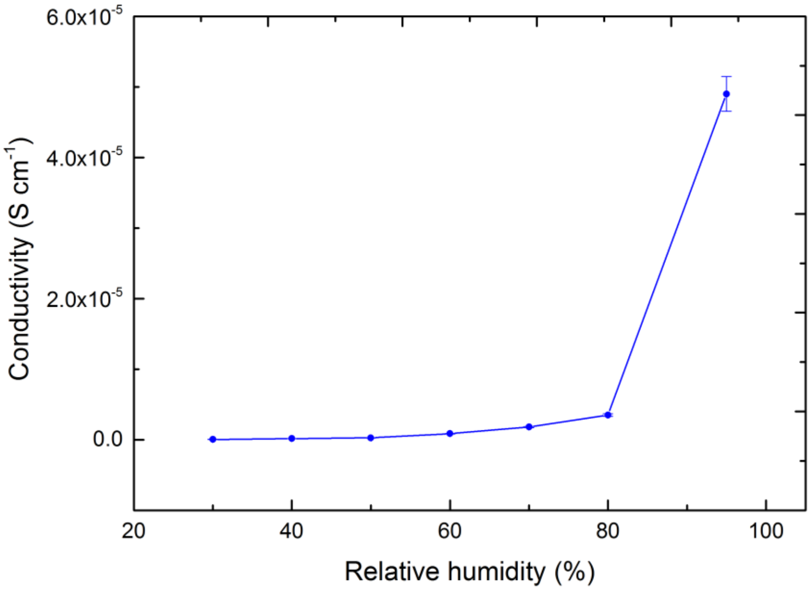

Supplementary Figure 47. Proton conductivity of **2** at 293 K subjected to variable relative humidity. The error bars shown represent the value of ±1 standard error of the mean of four consecutive samples that were tested, which are defined as $\sigma/\sqrt{n}$, where σ = standard deviation and n = number of samples tested.

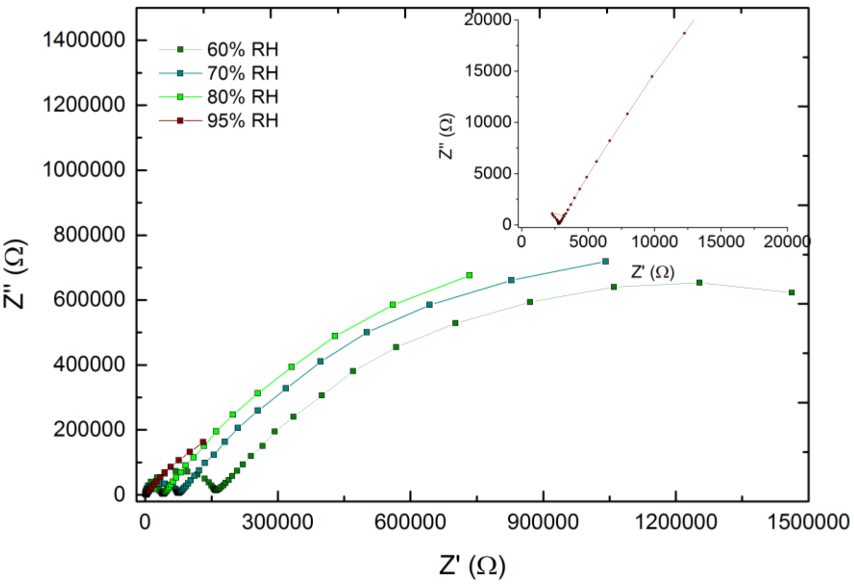

Supplementary Figure 48. Nyquist plots for **2** at 293 K subjected to variable relative humidity between 1 MHz and 0.1 Hz.

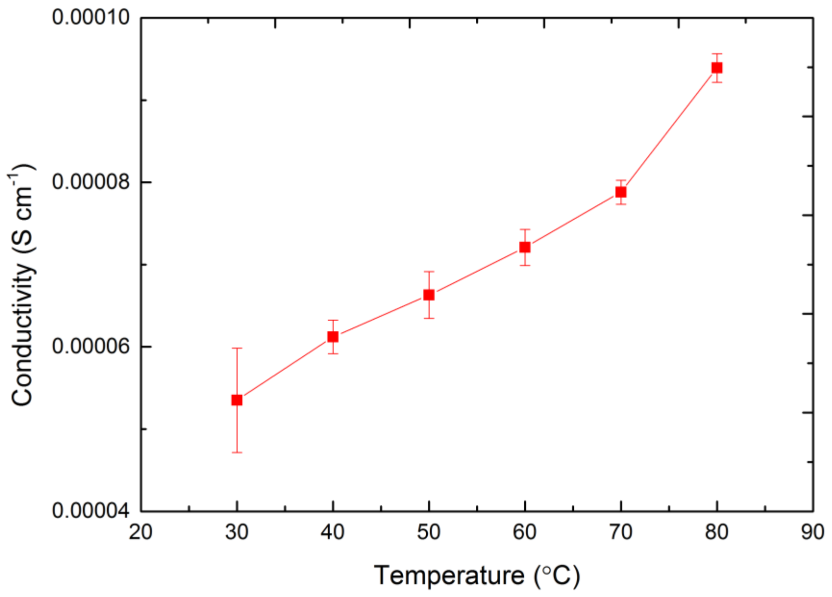

Supplementary Figure 49. Conductivity of **2** subjected to variable temperature under 95 % RH. The error bars shown represent the value of ±1 standard error of the mean of four consecutive samples that were tested, which are defined as σ/√n, where σ = standard deviation and n = number of samples tested.

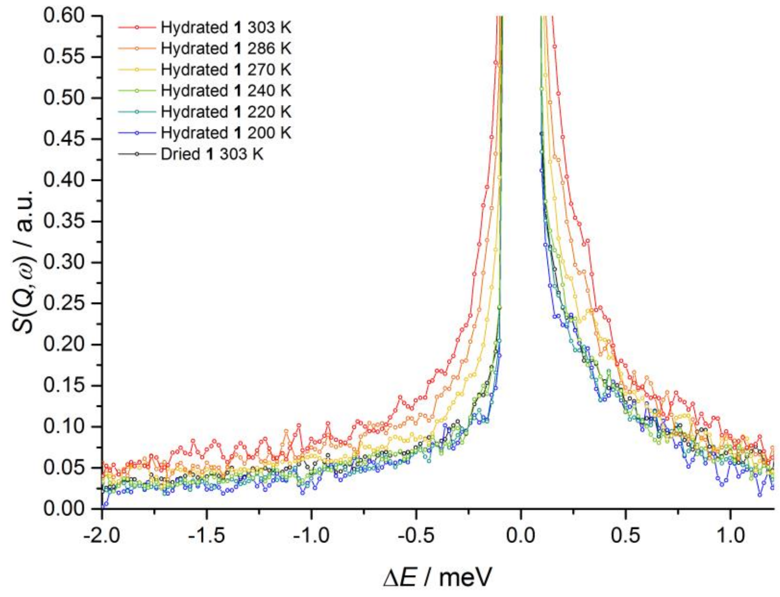

Supplementary Figure 50. QENS data for dried and hydrated **1** obtained by DCS at Q = 0.61 $Å^{-1}$. It is clearly shown that the hydrated samples have more obvious QENS signals (broadening) than the dry ones (black lines in the pictures). Also, the QENS signals of hydrated **1** increase with temperature.

Initial fits of the spectra for hydrated **1** were of poor quality at temperatures above 240 K, and the residual plot showed a distinct curvature. An additional Lorentzian component was introduced (Supplementary Fig. 51), which improved the overall fit. The broad width of this peak corresponds to a faster diffusion process. At low temperature and particularly for small Q values, the broader of the two Lorenzian components was weak and difficult to distinguish from background and the narrower QE peak. This results in refinement of the area to zero, in some cases, and large uncertainties for the extracted parameters.

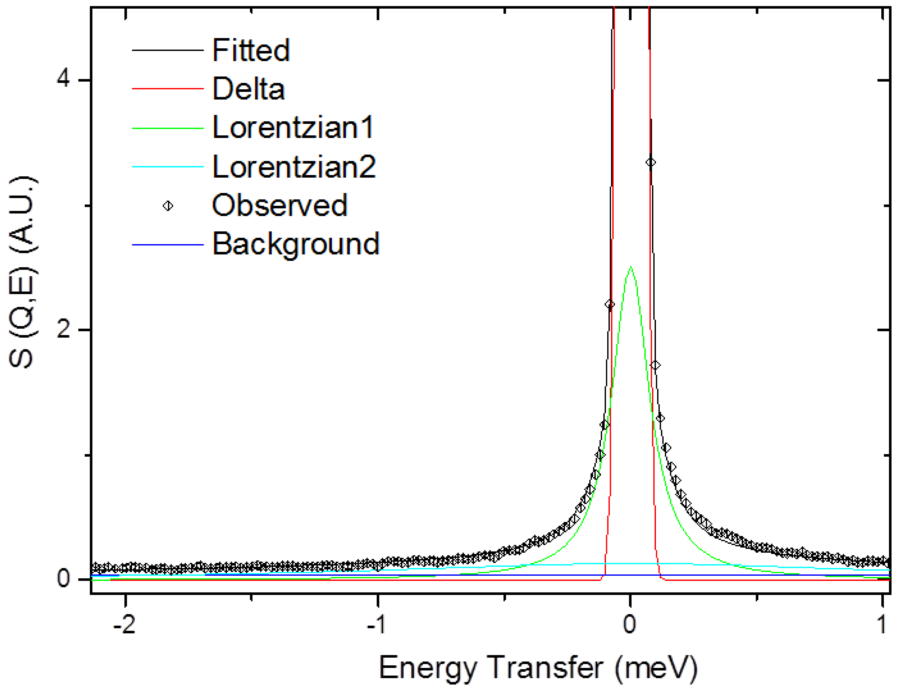

Supplementary Figure 51. QENS data for a Q-slice (1.22 Å^{-1}) of hydrated **1** at 303 K. Data (black line) are fit to a model comprising the resolution function (red line), a narrow Lorentzian (green line) and broad Lorentzian component (cyan line). Error bars indicate one standard deviation based on counting statistics.

The HWHM Γ of the narrow Lorentzian, arising from the slower of the two observed motions, is, within error, essentially independent of Q (Supplementary Fig. 52). A timescale for this reorientational motion at 303 K is estimated from $1/\Gamma$ as τ = 11.9(4) ps. The HWHM Γ of the broad component shows pronounced deviation from the linear DQ^2 at higher Q values, i.e. for shorter distances, and this is consistent with a jump-diffusion process (Supplementary Fig. 54). Fitting of the data to a number of jump-diffusion models was attempted, but this was complicated by the weak QE signal and the observed Q-dependent behaviour was not well-modelled. The diffusion coefficient was estimated as D = 1.2(6) $\times 10^{-11}$ m^2 s^{-1} at 303 K from the linear DQ^2 relationship in the low Q region, corresponding to longer-range diffusional motions.

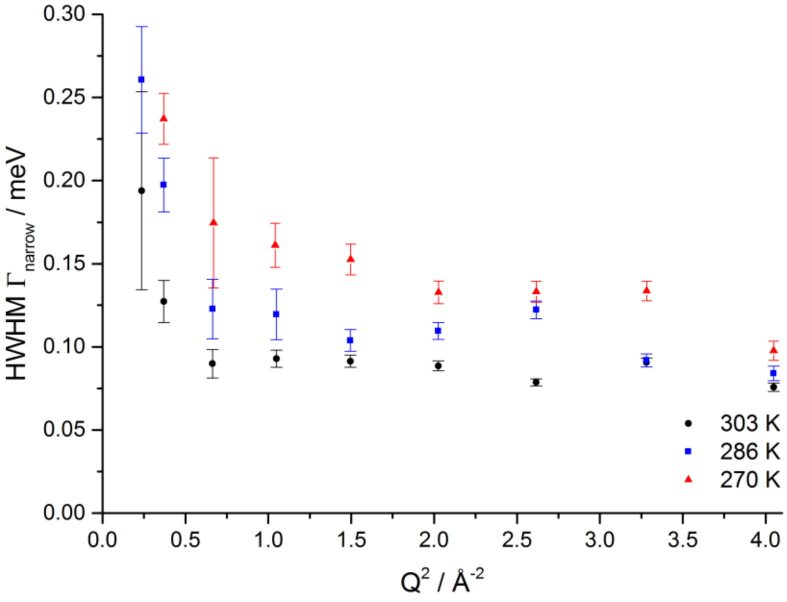

Supplementary Figure 52. Extracted half-width at half-maximum (HWHM) for the narrow Lorentzian of hydrated **1** plotted as a function of Q^2. Error bars represent a ±1 standard deviation.

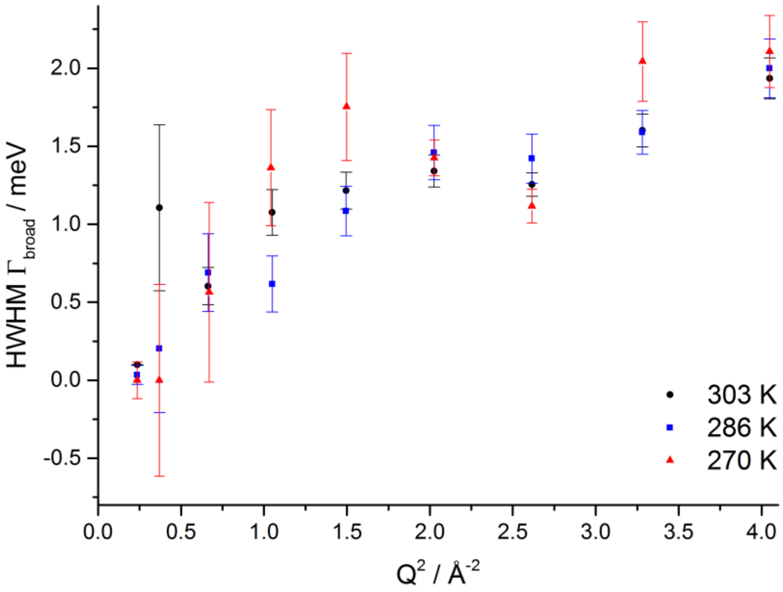

Supplementary Figure 53. Extracted half-width at half-maximum (HWHM) for the broad Lorentzian of hydrated **1** plotted as a function of Q^2. Error bars represent a ±1 standard deviation.

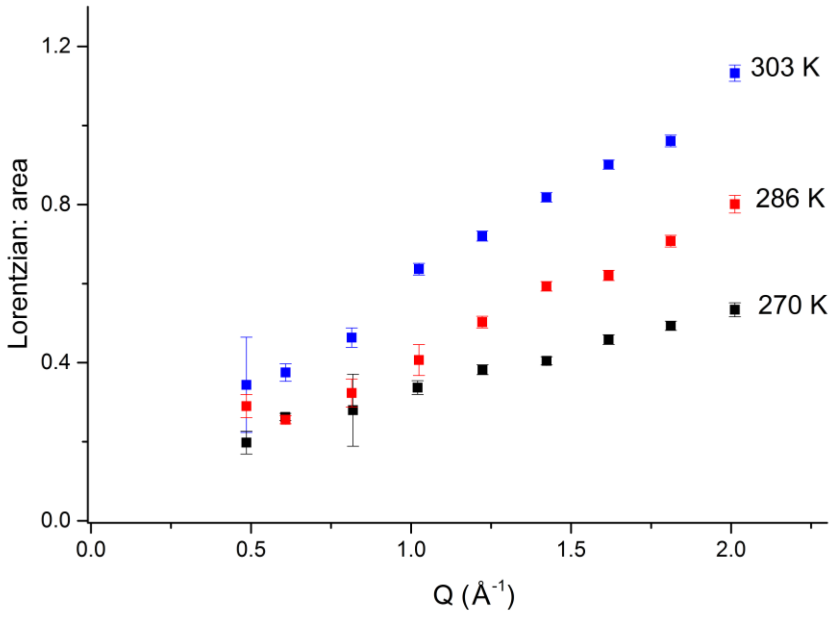

Supplementary Figure 54. Areas for the narrow Lorentzian component of the fit to the QENS peak of **1**. Error bars represent a ±1 standard deviation.

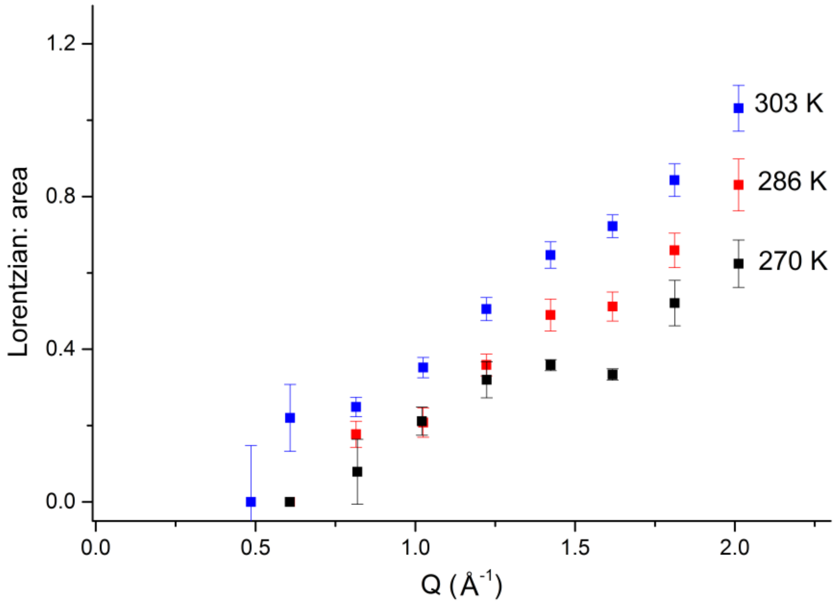

Supplementary Figure 55. Areas for the broad Lorentzian component of the fit to the QENS peak of **1**. Error bars represent a ±1 standard deviation.

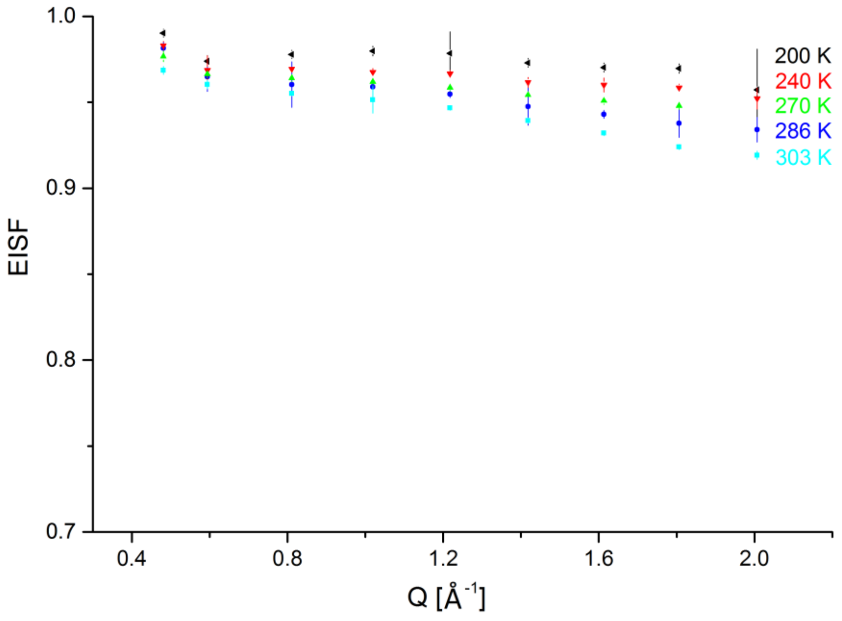

Supplementary Figure 56. Elastic incoherent structure factor (EISF) of hydrated **2** (200 K, 240 K, 270 K, 286 K and 303 K) from the data measured by Disk Chopper Spectrometer (DCS). Error bars represent a ±1 standard deviation.

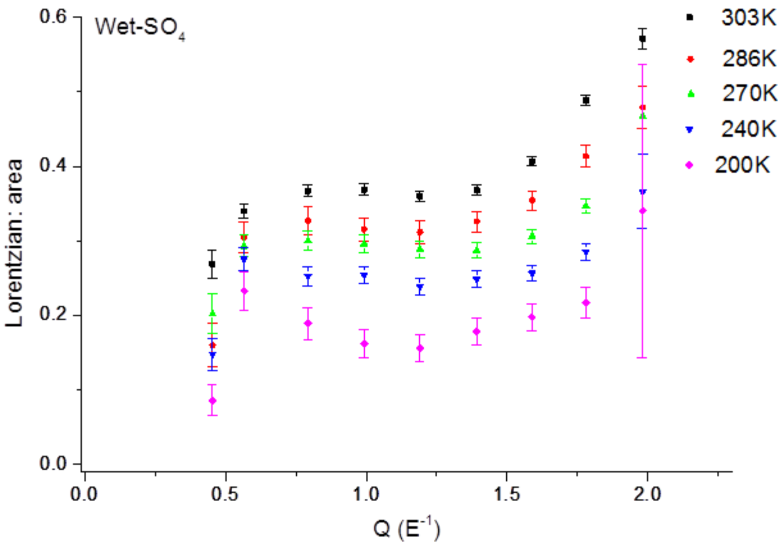

Supplementary Figure 57. Areas for the Lorentzian component of the fit to the QENS peak of **2**. Error bars represent a ±1 standard deviation.

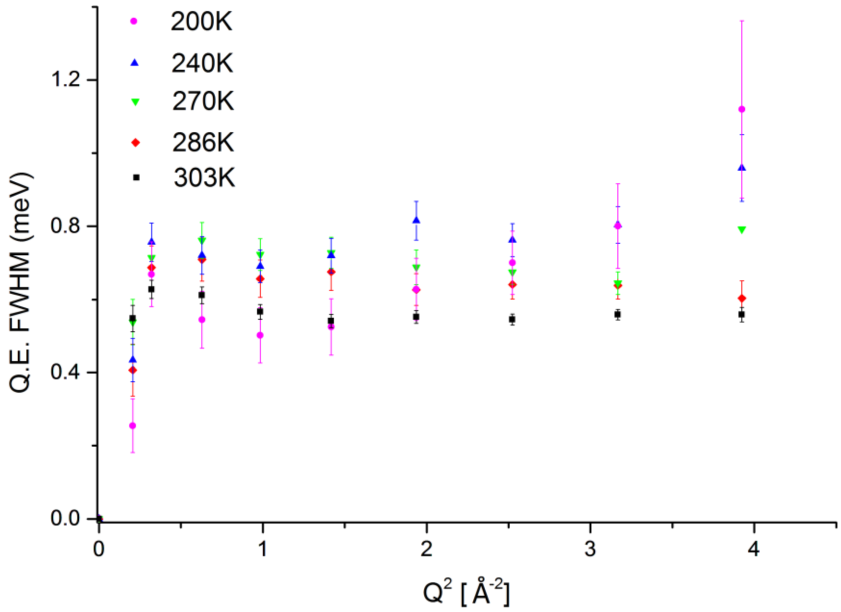

Supplementary Figure 58. Extracted full-width at half-maximum (FWHM) for the Lorentzian of **2** plotted as a function of Q^2. Error bars represent a ±1 standard deviation.

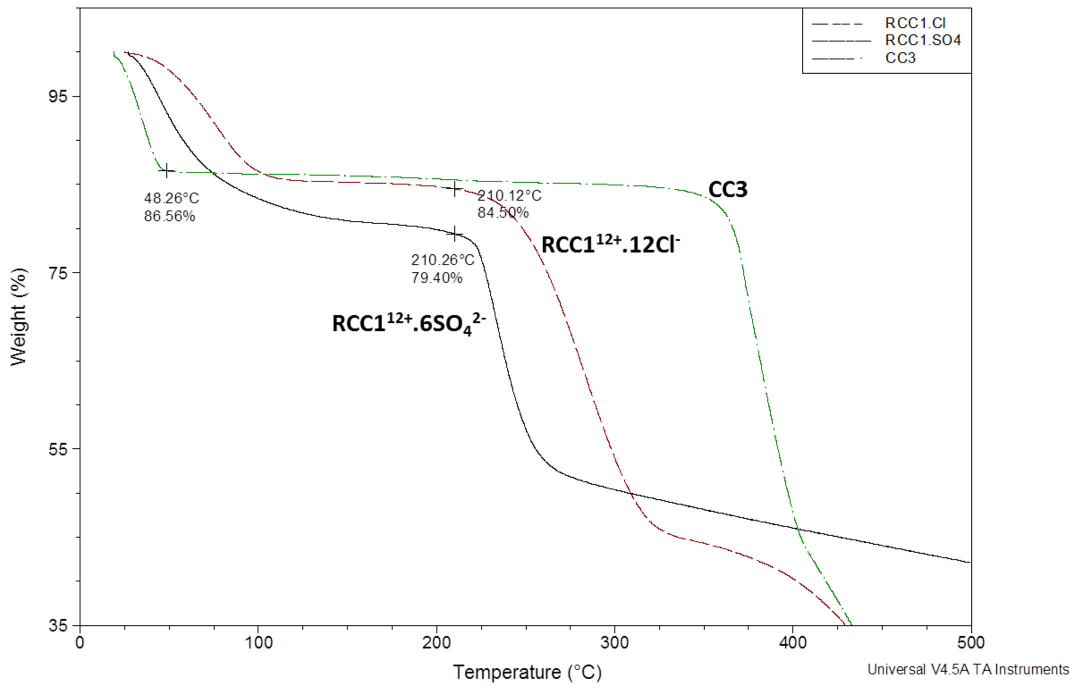

Supplementary Figure 59. TGA plots for **CC3**, **1** and **2** after being humidified at RH 95 % for 4 h.

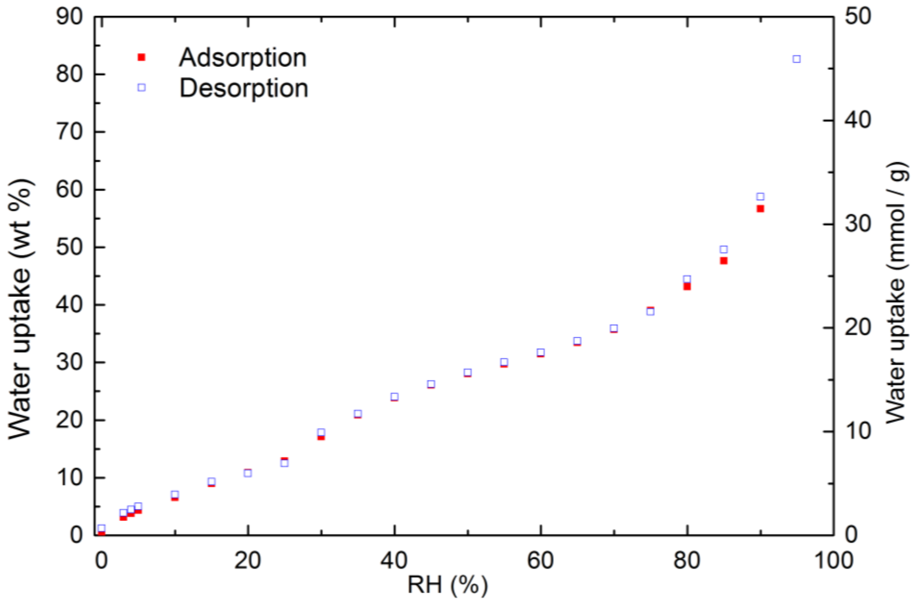

Supplementary Figure 60. Water sorption isotherm of **1** at 298 K.

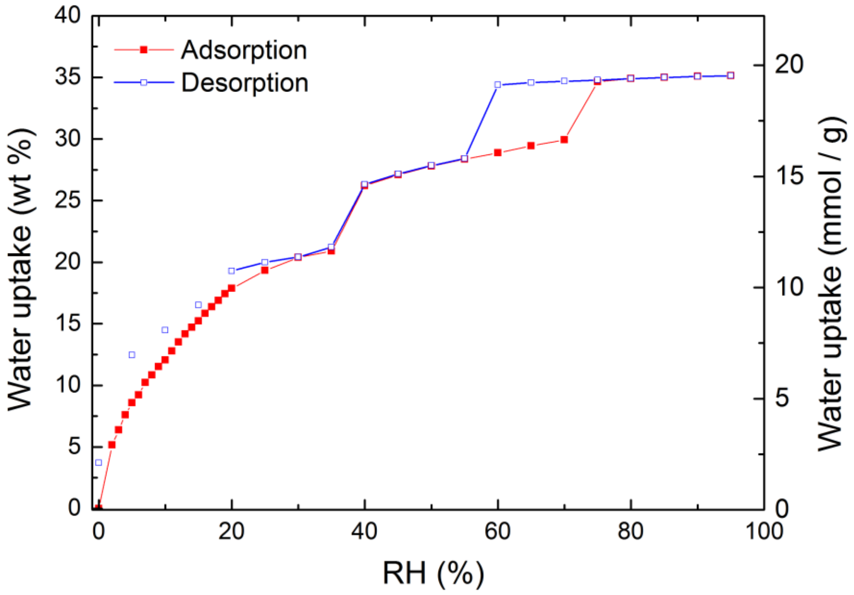

Supplementary Figure 61. Water sorption isotherm of **2** at 298 K.

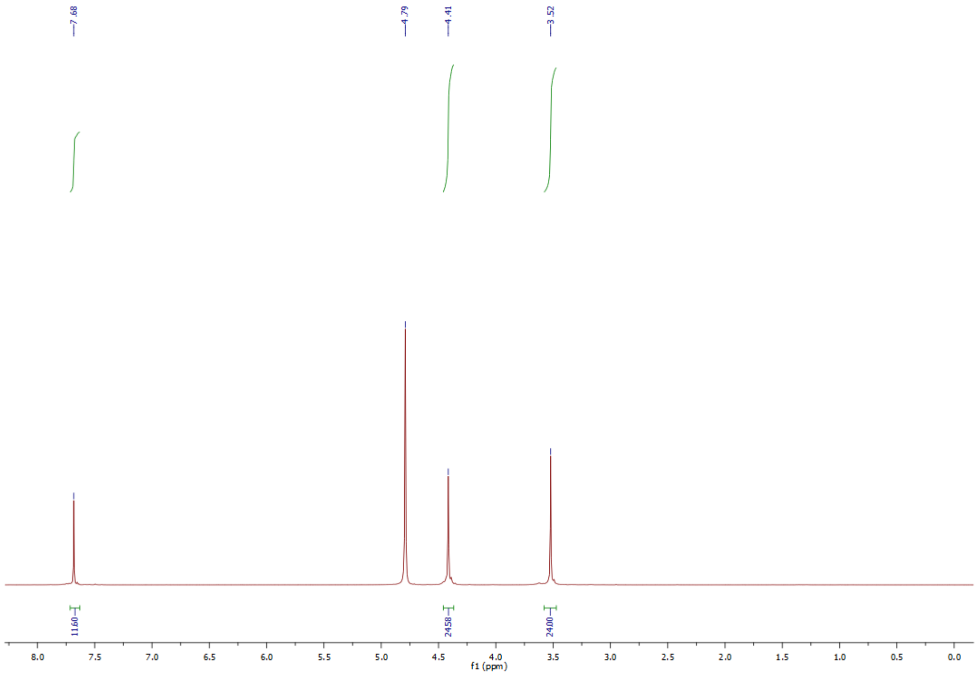

Supplementary Figure 62. ^{1}H NMR of **1** in D_2O at 25 ℃.

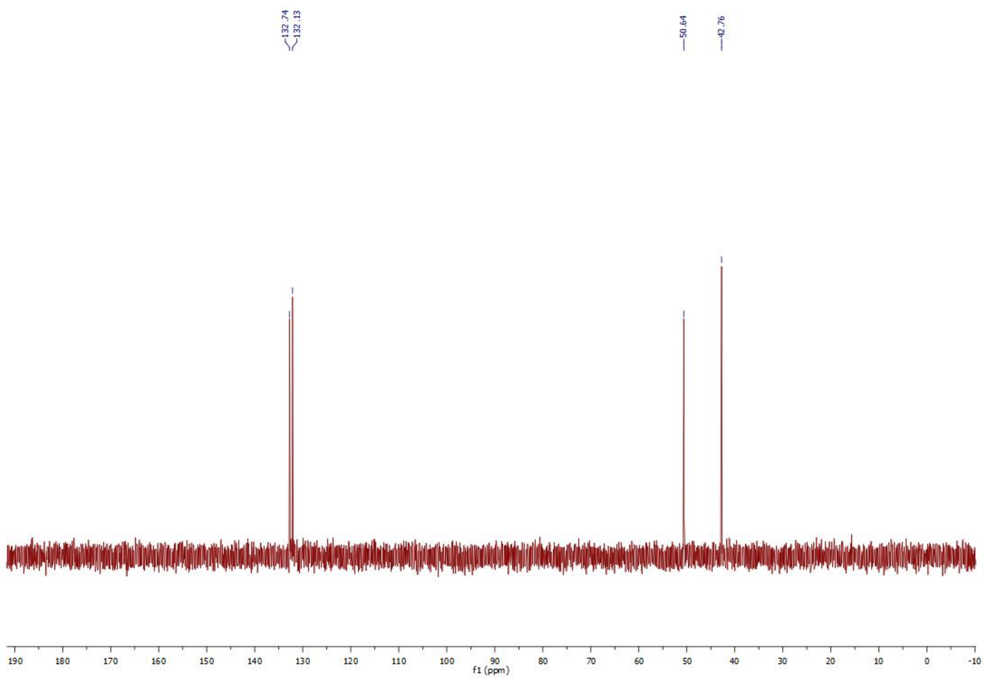

Supplementary Figure 63. ^{13}C NMR of **1** in D_2O at 25 ℃.

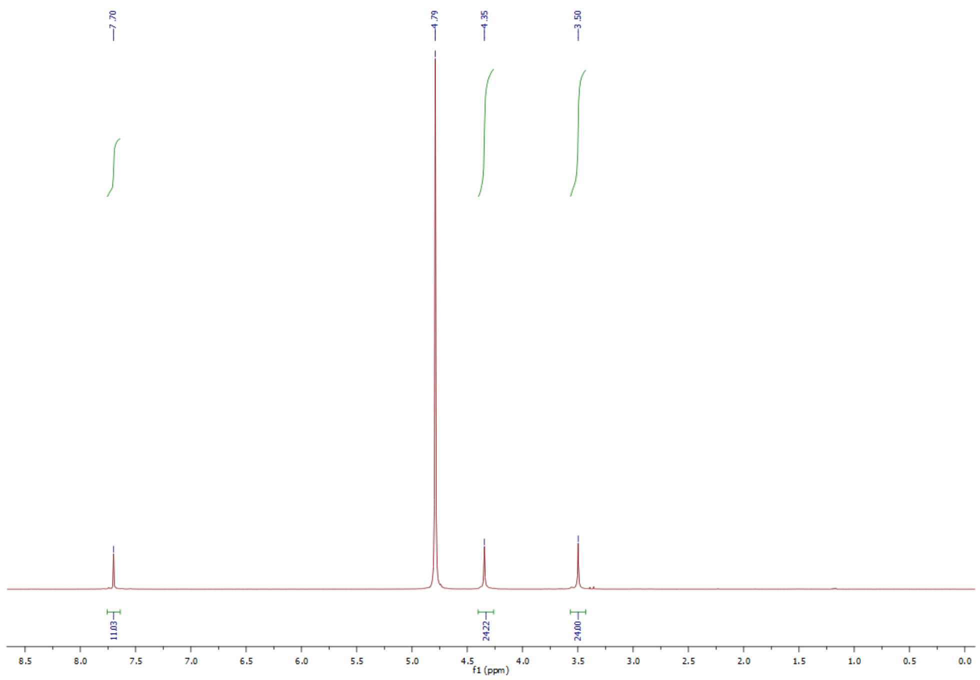

Supplementary Figure 64. ^{1}H NMR of **2** in D_2O at 25 ℃.

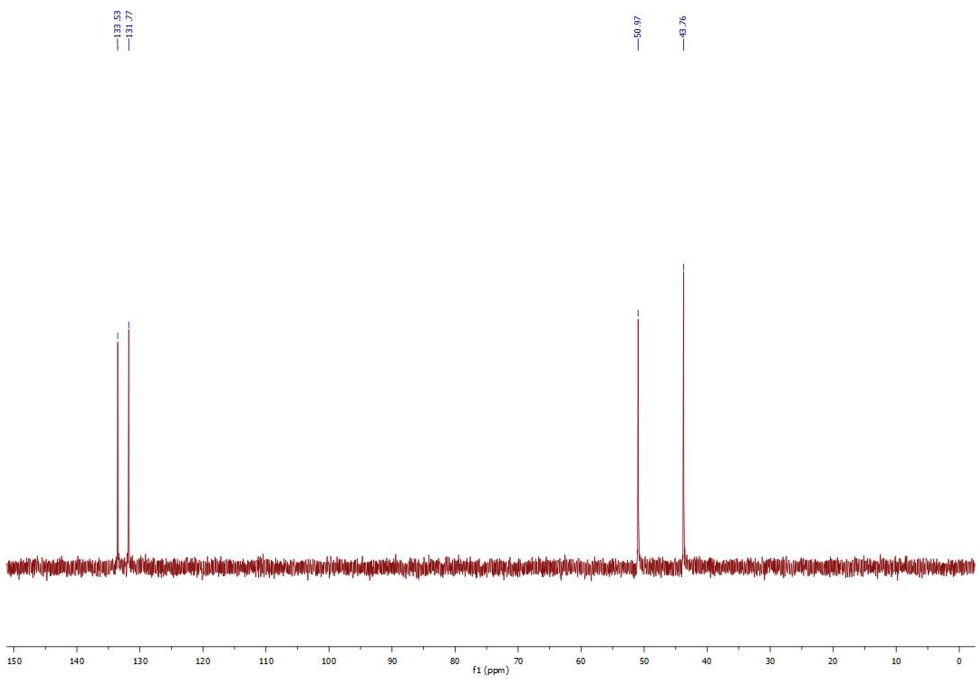

Supplementary Figure 65. ^{13}C NMR of **2** in D_2O at 25 ℃.

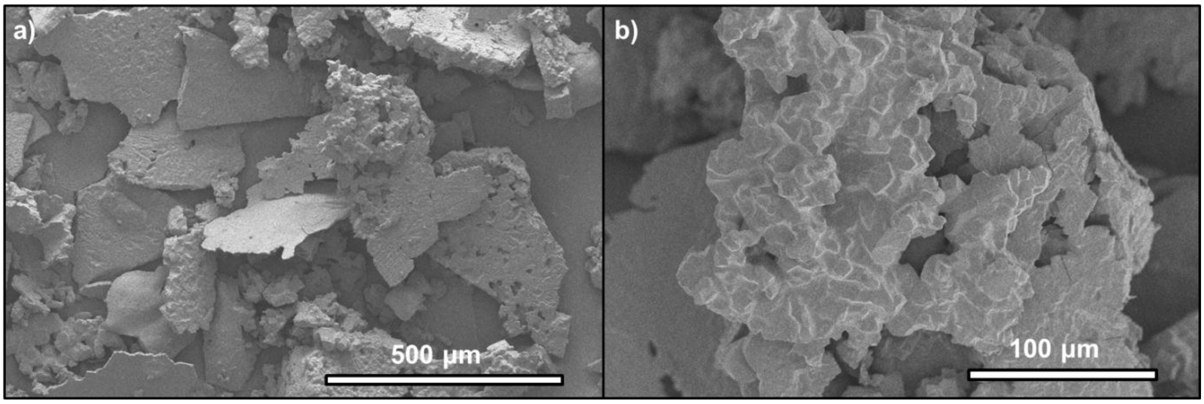

Supplementary Figure 66. SEM image of **1** as synthesized. The crystallites aggregate into connected conglomerates, a), but at higher magnification the geometric crystal habit can still be observed, b).

Supplementary Table 1. Cell parameters determined for **1** over relative humidity range 30–95 %.

**Relative humidity / %**	**atmosphere**	**30**	**50**	**70**	**95**
**Space group**	$I4_1$	$I4_1$	$I4_1$	$I4_1$	$I4_1$
a, b **/ Å**	20.265(1)	20.2982(8)	20.3078(8)	20.3032(8)	20.3142(8)
c **/ Å**	32.082(3)	32.146(2)	32.170(2)	32.167(2)	32.185(2)
V **/ Å^3**	13175(2)	13245(1)	13267(1)	13260(1)	13282(1)
R_{wp}, R_p **/ %**	9.13, 7.04	8.16, 6.39	8.03, 6.29	8.49, 7.66	8.11, 6.24
χ^2	1.06	1.14	1.12	1.11	1.10

Supplementary Table 2. Lattice parameters, variable-temperature PXRD for **2**.

**Temperature / K**	**288**	**293**	**298**	**303**	**308**	**313**		**318**	**323**	**328**	**333**
**Space group**	*Fdd*2	*Fdd*2	*Fdd*2	*Fdd*2	*Fdd*2	*Fdd*2	$I4_1/acd$	$I4_1/acd$	$I4_1/acd$	$I4_1/acd$	$I4_1/acd$
a **/ Å**	34.3223(4)	34.3248(4)	34.3243(4)	34.3265(4)	34.3289(4)	34.3294(5)	22.7693(8)	22.7938(5)	22.8097(4)	22.8300(4)	22.8491(5)
b **/ Å**	32.8716(4)	32.8717(4)	32.8697(4)	32.8674(4)	32.8661(4)	32.8644(4)					
c **/ Å**	31.3894(4)	31.4004(4)	31.4090(3)	31.4210(3)	31.4356(4)	31.4434(4)	34.295(2)	34.255(1)	34.236(1)	34.216(1)	34.198(1)
V **/ Å**^{3}	35414.3(7)	35429.5(7)	35436.5(6)	35449.8(7)	35467.4(7)	35474.9(8)	17780(2)	17798(1)	17812.2(8)	17833.4(8)	17854(1)
R_{wp}, R_p **/ %**	3.48, 2.65	3.44, 2.67	3.43, 2.66	3.49, 2.70	3.45, 2.67	3.18, 2.48		2.82, 2.22	2.77, 2.18	2.79, 2.18	3.20, 2.52
χ^2	1.31	1.30	1.29	1.32	1.30	1.20		1.07	1.05	1.07	1.15

Supplementary Note 1. Computational details.

Force-field-based, classical molecular simulations. Both Lennard–Jones (LJ) parameters and partial atomic charges for cage molecules were taken from the OPLS-AA force field[2]. Water molecules were modelled according to the so-called flexible simple point-charge water model (or SPC/Fw)[3]. In this model, the intramolecular degrees of freedom (including bond stretching and bond bending) of a water molecule are accounted for using harmonic potentials. Chloride ions were described by a simple additive, non-polarizable potential that was explicitly optimized for use with the SPC water model[4]. A recently-proposed fully flexible, classical force field was adopted for sulphate ions[5]. The Lorentz–Berthelot combining rules were used to calculate all the LJ cross-parameters except those between cage atoms, which were determined via geometric means (as adopted by OPLS-AA).

Calculation of potential of mean force (PMF) from constrained molecular dynamics simulations. PMF is basically the free energy profile of a pre-defined reaction path, along which a molecular process proceeds in the configuration space. The PMF, a function of reaction coordinate, specifies the free energy of the system corresponding to a thermal average over all degrees of freedom other than the reaction coordinate. With all the other degrees of freedom averaged out, the motion along the reaction coordinate identifies a highly probable path of diffusive motion which connects the initial state to the final state of the system. Here, we estimated the PMF for a respective molecular diffusion process from constrained molecular dynamics (MD) simulations. The reaction coordinate of such a process was defined as the distance between two molecular entities: *e.g.*, the distance between the centre of mass of a water molecule and the centre of mass of a cage molecule. A number of MD simulations were carried out with the system constrained to different points along the reaction coordinate (which was held fixed during the simulation). Particularly, the diffusion pathway was mapped out by performing constrained MD simulations sequentially—*i.e.*, the initial configuration of a simulation was taken as the final configuration of the proceeding one—with increments (in most cases) of 0.1 Å in the reaction coordinate. Integrating the mean constraint forces thereby obtained as a function of reaction coordinate thus yielded the changes in free energy along the reaction path.

All the constrained MD simulations were carried out in the isothermal–isobaric (*NPT*) ensemble at a temperature of 298.16 K and a pressure of 1 bar, using the DL_POLY molecular dynamics simulation package[S16]. The Martyna–Tuckerman–Klein variant of thermostat and barostat was used to maintain constant the temperature and pressure. The equations of motion were integrated, with a time step of 0.5 fs, using the velocity Verlet scheme, together with the RATTLE algorithm for solving bond constraints. A cutoff radius of 10 Å was applied to all LJ interactions, with energies and forces gradually shifted to zero when approaching the cutoff distance. The smoothed particle mesh Ewald method was used to handle electrostatic interactions. The simulation box consisted of one unit cell of the cage salt—*i.e.*, **1** or **2** —built from the crystallographic coordinates reported by single-crystal diffraction, with periodic boundary conditions exerted in three dimensions. Each MD simulation comprised a 7-ns equilibration run and an 8-ns production run.

Continuous fractional component Monte Carlo (CFCMC) simulations in the osmotic ensemble. CFCMC is an advanced MC technique devised to improve upon the deteriorating efficiency of insertion and deletion of molecules in expanded ensembles (*e.g.*, grand-canonical ensemble) when using, for example, conventional MC or configurational-bias MC for systems that are highly dense

and/or have slow ergodicity. Poor sampling in composition changes of the system, which is central to expanded ensembles, can also occur when there are specific interactions between species to insert/delete and the rest of the system. For example, the chance to successfully delete a strongly hydrogen-bonded molecule may be vanishingly small for the current conformation is energetically highly favourable. These considerations are of relevance here because the systems simulated have very limited empty spaces. Specifically, the experimental single-crystal **1** structure (with H_2O removed) was calculated to have a void fraction of *ca.* 5%, determined by a helium probe together with rigid cages and movable Cl^- ions (see simulation details, below). In other words, empty regions readily available to accommodate yet-to-insert H_2O molecules may not be abundant, especially in the extrinsic void space between the cages. Moreover, strong hydrogen bonds are expected to exist between the adsorbed H_2O molecules and between H_2O and host cage molecules and ions – deletions can be increasingly improbable as the H_2O loading goes up.

To overcome such difficulties, the CFCMC method expands the ensemble using a "fractional" (adsorbate) molecule that is associated with a coupling parameter λ. The parameter λ, which is confined between 0 and 1, gradually increases or decreases the intermolecular interaction between the fractional molecule and the rest of the system (*i.e.*, no interactions when $\lambda = 0$ and full interactions when $\lambda = 1$). Both Lennard–Jones and Coulomb interactions are scaled by a function of λ so that the fractional molecule can be forced into or out of the system. Briefly, the so-called λ moves successively change the value of λ by $\Delta\lambda$; when it exceeds 1 the current fractional molecule becomes fully present, whereas a λ value below 0 results in deletion of the fractional molecule. In both cases, a new fractional molecule is created with λ remaining between 0 and 1; that is, there is always a fractional molecule (and only one at a time) present in the system, which is not counted toward the adsorbed amount. The CFCMC approach allows for higher acceptance ratios of insertion and deletion, which is critical to achieve statistical reliability and accuracy of an expanded ensemble. For thermalization of the system (*i.e.*, the adjustment of surroundings to the new insertion/deletion), standard MC moves such as translation, rotation, and reinsertion can be used. Details on the CFCMC scheme and its implementation in RASPA can be found in the literature.

In this work, the CFCMC simulations were carried out in the osmotic ensemble. In this ensemble, the number of host atoms (*i.e.*, those of cages and ions), the total hydrostatic pressure, the temperature, and the fugacity of guest species are kept fixed, while the volume of the system and the number of guest molecules vary in accordance with the imposed pressure and fugacity. This hybrid MC–MD approach uses stochastic, open-system MC moves to change chemical composition of the system and uses deterministic MD trajectories to relax and help equilibrate the system. Since internal flexibility of the whole system are taken into account, such hybrid MC–MD methods, albeit computationally demanding, can largely remove most limitations introduced by the rigid-host assumption as adopted in grand-canonical MC simulations. This was found to be crucial to correctly simulating guest uptakes in the systems studied here. For example, a CFCMC simulation in the grand-canonical ensemble suggested a H_2O uptake of 6.8 % for the **1** crystal structure at 100 % relative humidity at 298 K. The prediction is well below the experimental value of 15.5 % (Supplementary Fig. 60), which was determined by TGA on a **1** sample that had been subjected to proton conduction measurements under similar conditions. By contrast, when the CFCMC method was used in conjunction with the osmotic ensemble a predicted H_2O uptake of 18.2 % was obtained, which is in better agreement with experiment.

A typical CFCMC simulation in the osmotic ensemble performed here started with an equilibration period of 1,000,000 MC cycles, each of which consisted of *n* MC steps, with *n* being equal to the number of adsorbate molecules (*i.e.*, *n* fluctuated during the simulation). The equilibration run was primarily aimed at calibrating, through the Wang–Landau sampling, the biasing factors for CFCMC, which were responsible for enforcing the system to visit all possible λ states equally probably. The simulation was continued for another 1,000,000 MC cycles for production of results. For both equilibration and production, system thermalization made use of translation, rotation, and reinsertion MC moves, together with a hybrid MC–MD move. In this hybrid move, an MD trajectory was developed in the isoenthalpic–isobaric ensemble for 2.5 ps, and the resulting configuration was then accepted or rejected with a probability (as with the other MC moves). Hybrid MC–MD moves were attempted for 10 % of the total MC steps, with the remaining steps equally split among the following moves: CFC insertion, CFC deletion, translation, rotation, and reinsertion (*i.e.*, 10:18:18:18:18:18). The Peng–Robinson equation of state was used to obtain fugacity of the adsorbing species (*i.e.*, H_2O) at given conditions. The starting configuration of the **1** salt (or **2** salt) was constructed from the experimental crystallographic data, which was gradually adapted to composition changes (and *vice versa*) during the simulation. A 1 × 1 × 1 unit-cell representation of the cage salt was used, together with periodic boundary conditions exerted in three dimensions. A real-space cutoff of 10.0 Å was applied to LJ interactions, and Coulomb interactions were computed using the Ewald summation technique with a relative precision of 10^{-6}. When the CFCMC simulation was carried out in the grand-canonical ensemble all cage atoms were kept fixed at their positions, whereas the ions were allowed for translational movements (rotations were also considered for SO_4^{2-}).

Molecular dynamics simulation of bulk water. The simulation box contained 216 water molecules, and periodic boundary conditions were used. Water molecules were placed into the box and then equilibrated using 10,000 MC cycles with translation, rotation, and reinsertion moves. Next, velocities were assigned according to the Maxwell–Boltzmann distribution and an MD equilibration was run for 2 ns, with a time step of 0.5 fs, in the *NPT* ensemble at 298.16 K and 1 atm. The *NPT* MD simulation was continued for 7 ns for production of results. A cut-off radius of 9.0 Å was used for all LJ interactions with standard tail corrections applied, while Coulomb interactions were computed using the Ewald summation technique with a relative precision of 10^{-6}. The flexible simple point-charge water model (SPC/Fw) was used to describe the water molecules. The simulations were performed with the RASPA code.

Supplementary Methods

Materials. 1,3,5-Triformylbenzene was purchased from Manchester Organics, UK. All other chemicals were purchased from Sigma-Aldrich and used as received. **CC1** (Cage 1) and **RCC1** (reduced Cage 1) was prepared as previously reported[6]. **CC3** (Cage 3) was synthesized as previously described[7]. Previous tests have shown **CC3** to be stable to boiling in water[8]. Certain commercial equipment, instruments, or materials are identified in this document. Such identification does not imply recommendation or endorsement by the National Institute of Standards and Technology nor does it imply that the products identified are necessarily the best available for the purpose.

Solution NMR. Solution ^{1}H NMR spectra were recorded at 400.13 MHz using a Bruker Avance 400 NMR spectrometer. ^{13}C NMR spectra were recorded at 100.6 MHz.

Fourier transform infrared spectroscopy (FTIR). IR spectra were collected on a Bruker Tensor 27 spectrometer. Samples were analyzed as KBr disks for 16 scans with a resolution of 4 cm^{-1}. Spectra were recorded in transmission mode.

Thermogravimetric analysis. TGA analysis was carried out using a Q5000IR analyzer (TA instruments) with an automated vertical overhead thermobalance. The samples were heated at the rate of 5 ℃ /min.

Electron microscopy. Imaging of the crystal morphology was achieved using a Hitachi S-4800 cold field emission scanning electron microscope (FE-SEM) operating in both scanning and transmission modes. Scanning-mode samples were prepared by depositing dry crystals on 15 mm Hitachi M4 aluminum stubs using an adhesive high-purity carbon tab before coating with a 2 nm layer of gold using an Emitech K550X automated sputter coater. Imaging was conducted at a working distance of 8 mm and a working voltage of 3 kV using a mix of upper and lower secondary electron detectors. Transmission-mode samples were prepared by dispersing the cage particles in a methanol suspension and depositing onto carbon-coated copper grids (300 mesh), imaging at 30 kV working voltage and 7 mm distance.

Gas sorption analysis. Gases of the following purities were used: hydrogen (99.9995% - BOC gases) and carbon dioxide (SCF grade – BOC gases). Surface areas and pore size distributions were measured by nitrogen adsorption and desorption at 77.3 K using a Micromeritics ASAP 2020 volumetric adsorption analyzer. Samples were degassed at offline at 80 ℃ for 15 h under vacuum (10-5 bar) before analysis, followed by degassing on the analysis port under vacuum, also at 80 ℃. Carbon dioxide isotherms were measured at 289 K using a Micromeritics 2420 volumetric adsorption analyzer using the same degassing procedure.

Water isotherm. Water adsorption isotherms, at 25 ℃, were determined gravimetrically using a DVS Advantage 2 (Surface Measurement Systems Ltd). In order to ensure the sample was fully de-solvated before beginning analysis, the sample was heated to 100 ℃ in a flow of nitrogen until a constant weight was achieved. During the isotherm determination, equilibrium was defined as a weight change of <0.001 % / min, and it had to stay below that criterion for a period of 10 minutes before moving on

to the next humidity. The experiment was performed at atmospheric pressure in a flow of nitrogen, with the humidity being generated / controlled by mass flow controllers / bubblers.

High resolution mass spectrometry. High resolution mass spectrometry (HRMS) was carried out using an Agilent Technologies 6530B accurate-mass QTOF mixed ESI/APCI mass spectrometer (capillary voltage 4000 V, fragmentor 225 V) in positive-ion detection mode. The mobile phase was 1:1 methanol : water containing 0.1 % formic acid at a flow rate of 0.5 mL/min.

Supplementary References

1. Pak, Y.S. & Xu, G. Proton transference number of PFSA ionomer membranes, *Solid State Ionics*, **60**, 347–350 (1993).
2. Jorgensen, W. L., Maxwell, D. S. & Tirado-Rives, J. Development and testing of the OPLS all-atom force field on conformational energetics and properties of organic liquids. *J. Am. Chem. Soc*. **118**, 11225–11236 (1996).
3. Wu, Y., Tepper, H. L. & Voth, G. A. Flexible simple point-charge water model with improved liquid-state properties. *J. Chem. Phys*. **124**, 024503, (2006).
4. Joung, I. S. & Cheatham, T. E. Determination of Alkali and Halide Monovalent Ion Parameters for Use in Explicitly Solvated Biomolecular Simulations. *J. Phys. Chem. B* **112**, 9020–9041 (2008).
5. Williams, C. D., Burton, N. A., Travis, K. P. & Harding, J. H. The Development of a Classical Force Field To Determine the Selectivity of an Aqueous Fe^{3+}–EDA Complex for TcO_4^- and SO_4^{2-}. *J. Chem. Theory Comput.* **10**, 3345–3353 (2014).
6. Swamy, S. *et al*. A metal-organic framework with a covalently prefabricated porous organic linker. *J. Am. Chem. Soc.* **132**, 12773–12775 (2010).
7. Tozawa, T. *et al*. Porous organic cages. *Nature Mater*. **8**, 973–978 (2009).
8. Hasell, T. *et al*. Reversible water uptake by a stable imine-based porous organic cage. *Chem. Commun.* **48**, 4689–4691 (2012).
